# A Rac-specific competitive inhibitor of guanine nucleotide binding reduces metastasis in triple-negative breast cancer

**DOI:** 10.1016/j.xcrm.2025.102233

**Published:** 2025-07-08

**Authors:** Florian Dilasser, Lindsay Rose, Agnès Quemener, Yann Ferrandez, Dorian Hassoun, Morgane Rousselle, Hugo Bergereau, Séverine Marionneau Lambot, Luciano E. Anselmino, Camille Trouillet, Gwennan Andre, Mike Maillasson, Mikael Croyal, Matthieu Riviere, Didier Dubreuil, Sylvain Collet, Frédérique Souaze, Mario Campone, Anne Patsouris, Erwan Mortier, Mauricio Menacho Marquez, Philippe Juin, Jacques Lebreton, Arnaud Tessier, Jacqueline Cherfils, Gervaise Loirand, Vincent Sauzeau

**Affiliations:** 1 Nantes Université, CHU Nantes, CNRS, INSERM, l’institut du thorax, Nantes F-44000, France; 2 Nantes Université, Inserm, CNRS, CRCI^2^NA, Nantes F-44000, France; 3 Laboratory of biology and applied pharmacology, CNRS, ENS Paris-Saclay, Paris, France; 4 Nantes Université, CNRS, CEISAM, UMR 6230, Nantes F-44000, France; 5 Nantes Université, CHU Nantes, CNRS, Inserm, BioCore, US16, SFR Bonamy, Nantes, France; 6 Instituto de Inmunología Clínica y Experimental de Rosario (IDICER CONICET-UNR), Centro de Investigación del Cáncer de Rosario. Facultad de Ciencias Médicas, Rosario, Santa Fe 3100, Argentina

**Keywords:** RAC1, drug discovery, invasive cancer, triple-negative breast cancer

## Abstract

The dysregulation of RAC1 activity is associated with neoplastic transformation, metastasis, and poor prognosis in several cancers. Here, we discover *in silico* a series of RAC1 inhibitors. The most potent of them, A41, specifically inhibits RAC1 with an original mechanism of action. We characterize A41 as a reversible inhibitor that competes with guanine nucleotide binding specifically on RAC proteins. A41 efficiently blocks RAC1 activity and RAC1-dependent cell functions including cell adhesion and migration. Chronic administration of A41 exhibits anti-metastatic effects in mouse models of triple-negative breast cancer, leading to an increase in the survival rate. Our findings suggest that this molecule, A41, could be a promising and powerful therapeutic agent for limiting invasive cancers in patients.

## Highlights

• A41 is a specific and reversible inhibitor of RAC proteins
• A41 competes with guanine nucleotide binding
• A41 blocks RAC1 activity and RAC1-dependent cell functions in cancer cells
• A41 exhibits anti-metastatic effects and increase of the survival rate

## Graphical abstract

**Figure:**
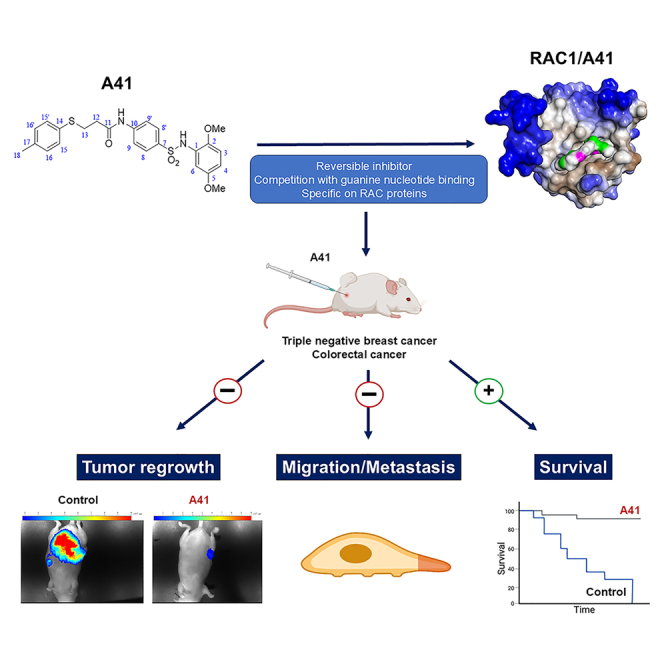

## Teaser

Dilasser et al. identified A41 as a specific RAC inhibitor. Chronic administration of A41 exhibits anti-metastatic effects in experimental models of invasive cancers (breast and colorectal cancers), leading to an increase in the survival rate.

## Introduction

The RAS-related small G protein member of the RHO family, RAC1, is a finely regulated molecular switch cycling between an inactive GDP-bound state and an active GTP-bound state to control essential cellular functions such as actin cytoskeleton organization, cell adhesion, cell movement, vesicle transport, oxidative stress, cell cycle, and gene expression.^1^^,^^2^ RAC1 is expressed in a wide variety of cells and tissues in which it acts as a downstream effector of numerous receptors, acting as a hub integrating upstream signals to coordinate appropriate cell responses.^3^ Deregulated expression or activation of RAC1 signaling leads to the generation of anarchic cellular responses that contribute to pathological processes, in particular cancers, by promoting neoplastic transformation, progression, invasion, and metastatic dissemination.^3^^,^^4^ Indeed, overexpression of RAC1 has been reported in colorectal,^5^ pancreatic,^6^ breast,^7^^,^^8^ and testicular cancers^9^ and various types of leukemia.^10^^,^^11^^,^^12^ Furthermore, increased expression of RAC1 is associated with poor differentiation, high pathological stage, and lymph node metastasis and correlated with poor clinical prognosis in various cancers, including upper urinary tract and primary gallbladder cancers,^13^^,^^14^ renal cell and hepatocellular carcinomas,^15^^,^^16^ gastric tumor,^17^ lung cancer,^18^ epithelial ovarian cancer,^19^ and breast cancer.^8^^,^^20^

The misregulation of RAC1 in cancer is also frequently related to the deregulation of molecular mechanisms involved in the control of RAC1 activity, degradation, or localization.^3^ In particular, some of the guanine nucleotide exchange factors (GEFs) that activate RAC1 by exchanging GDP for GTP, such as TIAM1, ECT2, PREX, and VAV family members, have been found to be overexpressed or mutated in cancer.^3^ These data suggest a causal role of the overactivation of RAC1 in tumorigenesis, although this has never been directly demonstrated.

In addition to the numerous pre-clinical studies demonstrating the involvement of RAC1 in tumorigenesis, proliferation, and metastatic events, recent works have described the role of RAC1 in the development of treatment resistance,^21^^,^^22^^,^^23^ thus strengthening the potential therapeutic value of RAC1 blockade to suppress tumor progression and metastasis. This also suggests that drugs targeting RAC1 may be useful in combination with classic chemo- and radiotherapies for the treatment of numerous aggressive cancers.

However, there are currently no clinically available drugs targeting RAC1. Like other members of the RAS protein superfamily, RAC1 has been considered too smooth, too floppy, and lacking pockets to which small molecules can bind tightly to be druggable.^24^ Several strategies have nevertheless been developed to inhibit RAC1^20^ by targeting the guanine nucleotide binding site of the protein (compounds EHT 1864 and GYS32661)^25^^,^^26^ or the RAC1/GEF interaction (NSC23766 and EHop-016).^27^^,^^28^^,^^29^ Unfortunately, the high IC_50_ (10–50 μM) and/or the poor specificity of these molecules make them unsuitable for therapeutical use in humans^30^ and limit their application, albeit very useful, for experimental purposes, as pharmacological tools for studying RAC1 functions and their regulation. More recently, it has been demonstrated that the MBQ-167 molecule displays a promising marked antitumor and anti-metastatic effect on triple-negative breast cancer (TNBC) cells MDA-MB-231 and MDA-MB-468 xenograft tumor in mice.^31^^,^^32^ Additionally, N,N′-disubstituted guanidines have been identified as effective Rac1 inhibitors with *in vivo* efficacy.^33^^,^^34^ For example, the compound 1A-116 has been shown to exhibit efficacy in an orthotopic IDH-wild-type glioblastoma model in mice.^34^ However, most of these molecules have the disadvantage of being a dual CDC42/RAC1 inhibitor, and this promiscuity may be responsible for potential adverse effects and could make it difficult to develop these molecules to clinical level.^20^

In this study, we carried out a pharmacophore and docking-based virtual screening, and we identified the compound A41 as a specific and efficient RAC1 inhibitor able to reduce RAC activity *in vitro* and *in vivo*. We also showed that A41 interferes with the binding of guanine nucleotides on RAC1 and provided evidence for the pharmacological/therapeutic potential of this RAC inhibitor in invasive human cancers such as TNBC.

## Results

### RAC1 activity is a poor prognostic factor of TNBC

**Figure 1:**
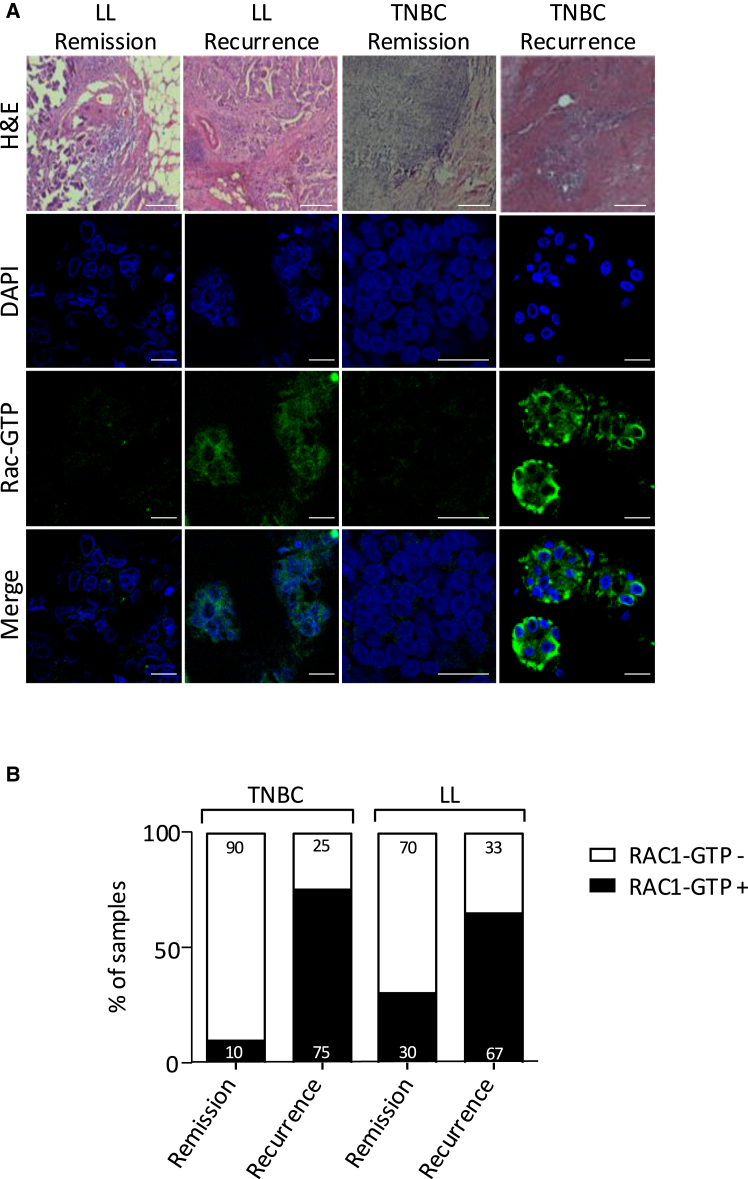
Analysis of RAC activity in breast cancer biopsies (A) RAC-GTP was detected by immunofluorescence in biopsies from patients with luminal B-like (LL, *N* = 20) and triple-negative (TNBC, *N* = 18) breast cancer. In each group, biopsies were divided into two subgroups: patients who have developed metastases (recurrence) and patients showing complete remission within the 5 years following the biopsy sampling (remission). H&E, hematoxylin-eosin staining (scale bars: 200 μm); DAPI, nucleus labeling; merge, fusion of DAPI and RAC-GTP images (scale bars: 16 μm). (B) Percentage of RAC-GTP-positive (white) and negative (black) biopsies in different patient groups.

**Figure 2:**
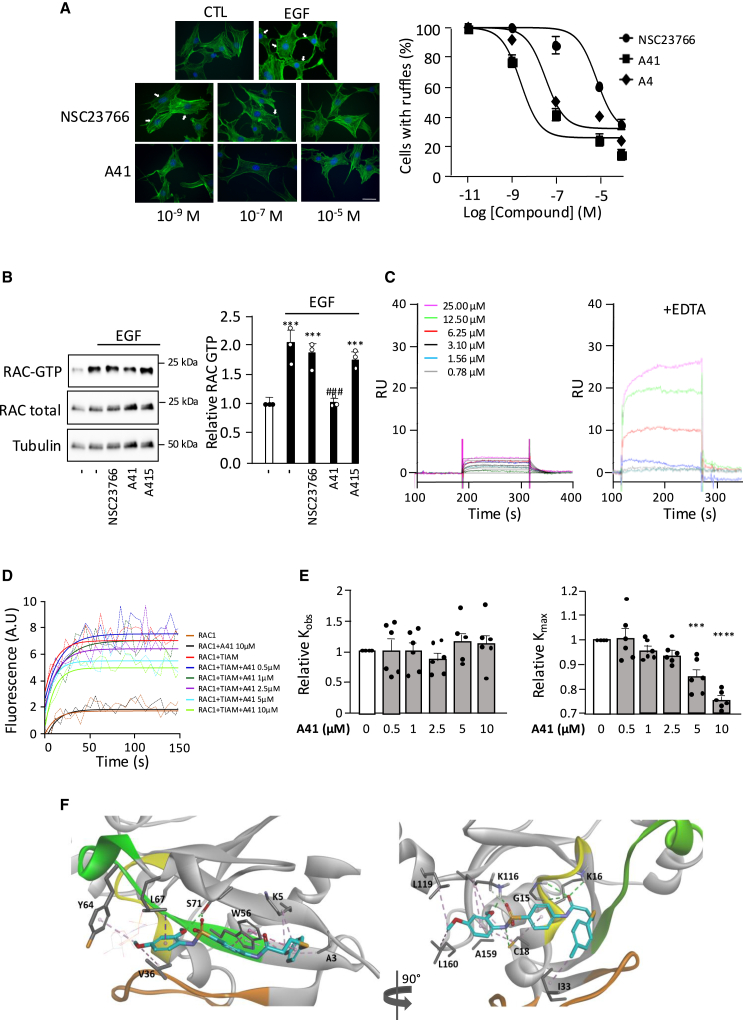
Identification of a new RAC1 inhibitor (A) Effect of A41 and NSC23766 on ruffle formation (white arrows) induced by EGF (10 ng/mL) in NIH 3T3 cells in serum-free culture medium (CTL). Inhibitors were added at indicated concentration 1 h prior to EGF stimulation. To assess cytoskeleton organization, cells were incubated with Alexa Fluor 488 phalloidin to visualize F-actin. Results shown are representative of 3 independent experiments. Corresponding concentration-response curves have been established by counting the percentages of cells with ruffles (scale bars: 10 μm). (B) Immunoblot analysis and associated quantification of RAC-GTP level and total RAC expression in NIH 3T3 cells stimulated by EGF (10 ng/mL, 5 min) and pre-incubated or not (−) with NSC23766, A41, or A415 at 10^−5^ M for 1 h (∗∗∗*p* < 0.001 vs. controls without EGF stimulation, ^###^*p* < 0.001 vs. controls with EGF stimulation) (*N* > 3). (C) Representative surface plasmon resonance (SPR) sensograms of binding of immobilized RAC1 with increasing concentrations of A41 with or without EDTA (20 mM) (*N* > 3). (D) Representative *in vitro* nucleotide exchange catalyzed by the DH-PH of TIAM (10 nM) measured by the binding of fluorescent mant-GTP (1 μM) to RAC1 (0.5 μM, preloaded with GDP) in the presence of indicated concentrations of A41 (out of *N* = 6). Dotted lines correspond to the experimental curves, and continuous lines represent the fit of experimental points. (E) K_obs_ and K_max_ were determined in each condition (*N* = 6). (F) Predicted binding modes of A4 to the NSC23766-binding site (left) or the GDP-binding pocket of RAC1 (right). In the NSC23766-binding site of RAC1, the compound A4 (carbon atoms in blue) makes 2 hydrogen bonds (green dotted line) with Ser71 (S71) and 10 hydrophobic interactions (pink dotted line) with Ala3 (A3), Lys5 (K5), Val36 (V36), Trp56 (W56), Tyr64 (Y64), and Leu67 (L67) leading to a docking score of −6.03 kcal/mol. The GDP molecule is shown with fine line. The P loop is shown in yellow, the switch 1 in orange, and the switch 2 in green. In the GDP-binding pocket, A4 establishes 6 hydrogen bonds with Gly15 (G15), Lys16 (K16), Cys18 (C18), and Lys116 (K116), 1 carbon hydrogen bond with Lys116, and 9 hydrophobic interactions with Cys18, Ile33 (I33), Lys116, Leu119 (L119), and Leu160 (L160), resulting in a docking score of −10.32 kcal/mol.

Previous studies identified RAC1 as a key player in various aspects of carcinogenesis and metastasis, and we confirmed that high levels of *RAC1* mRNA expression in cancer tissues from the Cancer Genome Atlas correlated with a high mortality rate (Figure S1). However, whether this high expression reflects a high level of activated RAC1 (RAC1-GTP) has not been assessed. We therefore analyzed the level of RAC1-GTP in a panel of human breast cancer samples from patients who had been followed for several years, including luminal B-like tumors (positive for either estrogen or progesterone receptor expression, HER2-negative status, and mitotic grade >1) and triple-negative (TNBC) tumors (lack of HER2 (Human Epidermal growth factor Receptor 2), estrogen, and progesterone receptors) (Table S1). Patients were divided into complete remission and recurrence (metastatic relapse) within 5 years after sampling to evaluate a possible association between RAC1 activity and breast cancer aggressiveness. The level of RAC1 activity was evaluated by the detection of RAC-GTP by immunofluorescence (Figure 1A). First, we verified under our experimental conditions that our RAC-GTP labeling did not correspond to vimentin labeling as described by Baker et al.^35^ (Figure 2). In both breast cancer subtypes, the level of active RAC1 was significantly higher in tumors from the recurrence groups than in those from the remission groups, the level of RAC1-GTP being low or undetectable in the latter (Figure 1A). Moreover, not only was RAC1 activity higher in the recurrence groups but also the proportion of biopsies positive for RAC1-GTP was considerably higher in the recurrence group than in the remission group for both cancer types (Figure 1B). This analysis suggests that the detection of RAC1 activity level in primary tumors could be predictive of metastatic relapse in breast cancers, with a high specificity and sensitivity in the case of TNBC. These results support the hypothesis that excessive activation of RAC1 is involved in the initiation of metastases and that inhibitors targeting RAC1 activation could have therapeutic value.

### Virtual screening identifies a new RAC1 inhibitor

**Figure 3:**
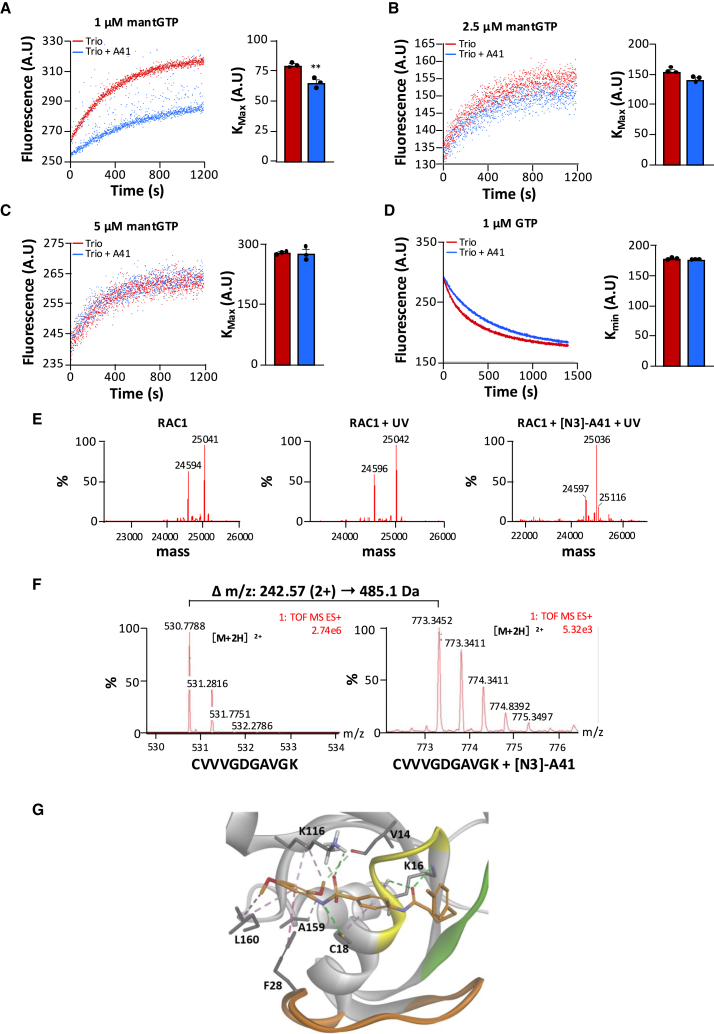
Inhibitory mechanism of A41 (A–D) RAC1 activity was monitored by the change in mant-GTP/GDP fluorescence induced by its binding to or releasing from RAC1 (A–D). GDP-bound RAC1 (0.5 μM) was incubated with TRIO (10 nM) and the indicated concentrations of mant-GTP without and with A41 (5 μM) (A–C). Mant-GDP-bound RAC1 (0.5 μM) was incubated with TRIO (10 nM) and GTP (1 μM) without and with A41 (5 μM) (D). AU: arbitrary fluorescence units. All experiments were done in triplicate. K_max_ and K_min_ are expressed as mean ± SEM. ∗∗*p* < 0.01 and ∗∗∗*p* < 0.001 vs. TRIO. (E) Deconvoluted mass spectra generated from crude mass spectrum analyses to evaluate RAC1 photolabeling with [N_3_]-A41 by LC-HRMS on native protein. (F) Representative mass spectra of both labeled and unlabeled CVVVGDGAVGK peptide during LC-HRMS analysis of RAC1 photolabeling with [N_3_]-A41 after trypsin digestion. (G) Predicted binding mode of A41 to the GDP-binding pocket of RAC1. Compound A41 (orange) makes 4 hydrogen bonds (green dotted line) with Lys16 (K16), Cys18 (C18), and Lys116 (K116), 2 carbon hydrogen bond (light green dotted line) with Val14 (V14) and Lys116 (K116), and 7 hydrophobic interactions (pink dotted line) with Cys18 (C18), Phe28 (F28), Lys116 (K116), Ala159 (A159), and Leu160 (L160). The P loop is shown in yellow, the switch 1 in orange, and the switch 2 in green.

The activation of RAC1 by GEFs, which induce the exchange of GDP for GTP and subsequently its interaction with its effectors, is characterized by the remodeling of two nucleotide-binding regions, called switch 1 and switch 2. Thus, molecules that bind these switch regions are expected to impair RAC1 interactions that drive its activity. We therefore generated a pharmacophore model on the basis of NSC23766, which has been proposed to bind at the back of the switch 2 according to the RAC1-GDP-NSC23766 crystal structure,^36^ which we used for virtual docking of 114,400 molecules (Figure 3A).

The 100 top-scoring chemicals thus identified were purchased to test their ability to inhibit RAC1 activity. All the molecules were effective at inhibiting processes dependent on RAC1 activities (membrane ruffle formation, migration, and adhesion) and at reducing the increased level of RAC1-GTP induced by epidermal growth factor (EGF) (Figure 3B). Molecule A4 was the most efficient at reducing the level of RAC1-GTP (Figure 3B) and has the lowest IC_50_ to inhibit membrane ruffle formation (Table S2). Analogs of A4 carrying various substitutions were then synthesized and tested for their ability to inhibit RAC1 activation (Table S2). The A41 analog, bearing a change in the methoxy group position on the benzene ring, was a more potent inhibitor of RAC1-dependent membrane ruffle formation than A4 (IC_50_ = 34.2 nM for A4 and 2.5 nM for A41) (Table S2; Figure 2A). On the contrary, removal of the aromatic ring (A420) or replacement of the sulfonamide group by an amide (A415) resulted in the loss of the inhibitory effect on RAC1, suggesting that these functional groups have an essential role in the inhibitory activity of these molecules. The inhibitory activity of A41 (10^−5^M) was further assessed on cell migration and adhesion, both processes known to depend on RAC1 activity. A41 efficiently inhibited both functions and was more active than NSC23766 (10^−5^M) (Figures S4A and S3B). Thus, A41 was chosen as the lead molecule of this chemical series, and A415 was used as a negative control. In order to further characterize the inhibitory properties of A41, we next directly assessed its effects on RAC1 activation in EGF-stimulated NIH/3T3 fibroblasts. Measurement of the amount of active GTP-bound RAC1 by pull-down assay showed that A41 at 10^−5^M blocked RAC1 activation, while NSC23766 and A415 at the same concentration had no significant effect (Figure 2B). Together, these observations show that A41 efficiently blocks RAC1 activity and RAC1-dependent functions in cells.

### Compound A41 inhibits RAC1 by competition with guanine nucleotides

Based on the crystal structure of complexes of RAC-family proteins with their GEFs,^37^ binding of A41 on RAC1 next to switch 2 is expected to impair its interactions with GEFs and thus inhibit GEF-stimulated nucleotide exchange. To confirm this model, we first checked that A41 directly bound to purified RAC1-GDP using surface plasmon resonance (SPR). Results show that A41 directly interacted with RAC1 (K_D_ = 30 nM), and interestingly, this binding was strongly increased in the presence of 20 mM of EDTA, which favors the dissociation of nucleotides from RHO protein in the absence of a GEF^38^ (Figure 2C). We next analyzed whether the dose-response curve of A41 affects *in vitro* GEF-induced activation of RAC1 by using the catalytic Dbl homology/ Pleckstrin homology (DH-PH) domain of the RAC-GEF TIAM and measuring the binding of fluorescent mant-GTP on RAC1 (Figure 2D). A41 decreased the plateau (K_max_) but not the kinetics (K_obs_) of the nucleotide exchange in a concentration-dependent manner without affecting the spontaneous activity of RAC1 (Figures 2D and 2E). This pattern of the inhibitory effect of A41, which only affects the plateau of the nucleotide exchange reaction, combined with the increased binding of A41 to RAC1 in the presence of EDTA, suggests that A41 could interact with the nucleotide-binding pocket of RAC1. Indeed, prediction of A41 binding modes by docking studies suggested that A41 interaction with RAC1 mainly involves hydrophobic interactions when docked to the NSC23766-binding site of RAC1 (the one used for the virtual screening), while it consists of more hydrogen bonds when the nucleotide-binding site was explored, resulting in a more favorable docking score for the latter site (Figure 2F). We therefore hypothesized that the inhibitory action of A41 on nucleotide exchange might result from competitive binding of A41 to the nucleotide-binding site rather than binding to the NCS23766-binding site. In that case, increasing the concentration of GTP in the exchange assay should reduce the inhibitory effect of A41. To test this hypothesis, we measured the effect of a fixed concentration of A41 (5 μm) on the kinetics of nucleotide exchange induced by TRIO on RAC1 in the presence of increasing mant-GTP concentration (Figures 3A–3C). The inhibitory effect of A41 was strongly decreased by increasing the mant-GTP concentration from 1 to 2.5 μM and was abolished at 5 μM. By contrast, A41 had no effect on TRIO-induced GDP dissociation (Figure 3D). These results suggest that during TRIO-induced nucleotide exchange, A41 competes similarly with GTP at the nucleotide-binding site. It also indicates that the affinity of A41 and guanine nucleotides for the nucleotide-binding site of RAC1 is of the same order. These observations are consistent with a competition mechanism between A41 and GTP binding on RAC1. Moreover, the inhibitory action of A41 on GTP loading on RAC1 is similar for both TIAM- (Figure 2D) and TRIO- (Figure 3A) induced nucleotide exchange reactions, suggesting that the effects of A41 are not RAC-GEF dependent.

To prove that A41 binds to the nucleotide-binding site, we performed photolabeling coupled with liquid chromatography-high resolution mass spectrometry (LC-HRMS) using a phenylazide derivative of A41 ([N_3_]-A41). [N_3_]-A41 displayed a docking score similar to that of the parent compound A41 and also retained its ability to inhibit RAC1 activation (Table S3). LC-HRMS chromatograms show that irradiation of RAC1 did not alter its protein profile, while in the presence of [N_3_]-A41, slight modifications of both chromatographic profile and mass spectrum associated with an increased noise level suggest that RAC1 was modified (Figure 3E). Deconvoluted mass spectra clearly identified the two typical states of RAC1 corresponding to the protein with (∼25.0 kDa) and without (∼24.5 kDa) bound nucleotide (Figure 3E). In the presence of [N_3_]-A41, the peak of the smallest RAC1 isoform was reduced, and a third state with a molecular weight of 25.1 kDa corresponding to the theoretical labeling of nucleotide-free RAC1 with a single molecule [N_3_]-A41 appeared (Figure 3E). By LC-HRMS and liquid chromatography-tandem mass spectrometry analyses, we observed that trypsin digestion led to the formation of 14 detectable peptides corresponding to 88% sequence coverage of RAC1. The spectrum of peptides from the [N_3_]-A41-treated RAC1 sample shows that it contained an additional doubly charged ion peptide corresponding to CVVVGDGAVGK (position 6–16) with a mass-to-charge (*m/z*) shift of 242.57 (i.e., 485.1 Da) and a retention time of 8.5 min (Figure 3F). Tandem mass spectrometry fragmentation of both labeled and unlabeled ^6^CVVVGDGAVGK^16^ peptides revealed that the residue lysine 16 (K16), a key residue in the P loop that binds to nucleotide phosphates, is the reactive amino acid linking the inhibitor (Table S4). In light of these results, we re-examined the docking of A41 in the nucleotide-binding site of RAC1. According to this refined model, A41 occupies most of the nucleotide-binding site, making contacts with the switch 1 and P loop regions in a manner that is incompatible with the binding of a nucleotide, in accordance with the competitive inhibition mechanism. Strikingly, assessment of the docking parameters of all A4 analogs in this pharmacophore revealed a very good correlation between the docking score and the potency to inhibit RAC1-dependent membrane ruffle formation (Table S2). These data provide a structural model for the binding of A41 into the nucleotide-binding site that fits with the experimental observations (Figure 3G).

Together, this set of results supports our hypothesis that A41 binds to the guanine nucleotide-binding pocket of RAC1 and validates a mechanism of action whereby A41 inhibits RAC1 activity by competing with GTP.

## A41 is a highly specific inhibitor of RAC proteins

**Figure 4:**
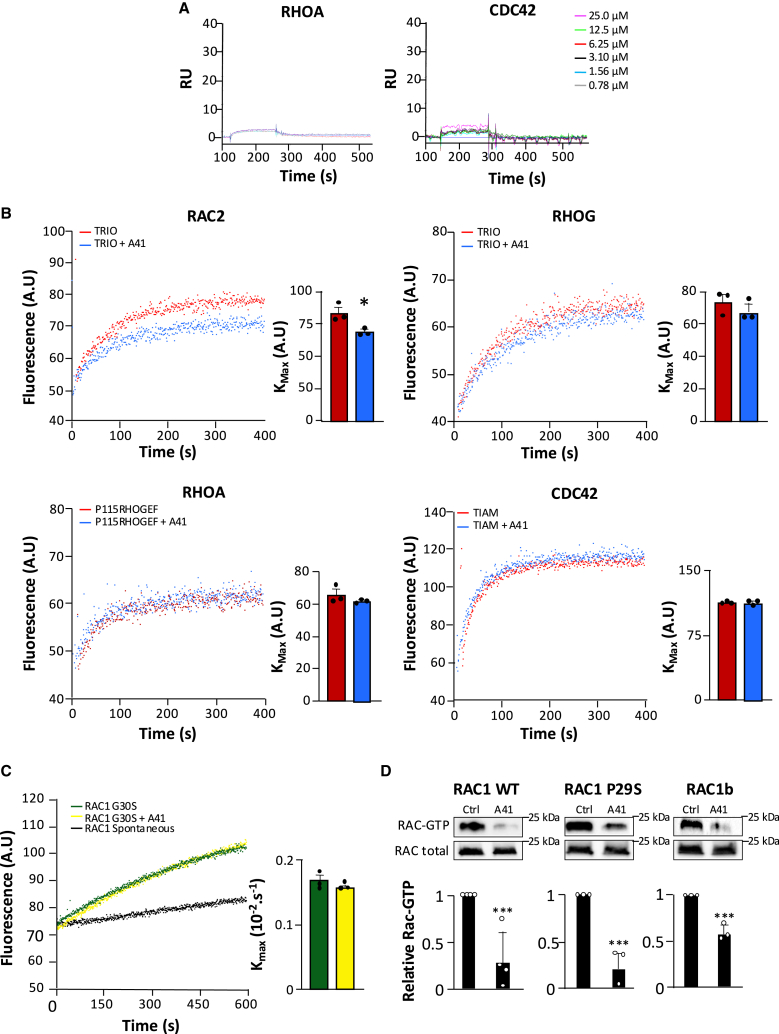
A41 selectively impairs RAC protein activation (A) Representative surface plasmon resonance (SPR) sensograms of binding of immobilized RHOA or CDC42 with indicated increasing concentrations of A41 (*N* > 3). (B) Effect of A41 on GEF-stimulated RAC2, RHOG, RHOA, and CDC42 nucleotide exchange. Purified small G proteins were pre-loaded with GDP and then nucleotide exchange was monitored by the increase in fluorescence following mant-GTP binding in the absence and presence of A41 (5 μM) (*N* = 3). K_obs_ is expressed as mean ± SEM. ∗*p* < 0.001 vs. control. (C) Effect of A41 on RAC1 G30S activation. RAC1 G30S activation was monitored by the increase in fluorescence following mant-GTP binding in the absence and presence of A41 (5 μM). The GEF TRIO was used to induce nucleotide exchange (*N* = 3). Data are expressed as mean ± SEM. (D) Immunoblot analysis and associated quantification of RAC-GTP level and total RAC expression in NIH-3T3 fibroblasts expressing RAC wild-type (RAC1 WT) or RAC1 oncomutants (RAC1 P29S and RAC1b) in the absence (Ctrl) and presence of A41 (10 μM). RAC activation was measured as the ratio of RAC-GTP to total RAC and expressed relative to Ctrl condition. Data are presented as mean ± SEM. ∗∗∗*p* < 0.001 vs. controls.

The nucleotide-binding site is highly conserved across the small G protein family^24^ and is thus considered as an unusable target for specific inhibition. The binding of A41 to the nucleotide-binding pocket of RAC1 thus prompted us to address its specificity for RAC proteins compared to other main members of the RHO protein subfamily. First, we assessed binding of A41 to these purified RHO proteins by SPR. No binding was measured for RHOA and CDC42 (Figure 4A), under conditions where binding to RAC1 was observed (Figure 2C). Next, we analyzed whether A41 affects the nucleotide exchange reaction using purified RHO family members and catalytic domains of their GEFs. A41 (10^−5^M) reduced TRIO-induced activation of RAC2, which is closely related to RAC1, but had no effect on TRIO-induced RHOG activation (Figure 4B). A41 (10^−5^M) did not change the activation of RHOA and CDC42 induced by the catalytic DH-PH domains of their GEFs p115RhoGEF and TIAM, respectively (Figure 4B). Thus, A41 shows specificity for RAC proteins, while the nucleotide-binding site is highly conserved within the RHO protein subfamily. Sequence analysis of RAC proteins reveals the unique Gly30 in switch 1 compared to other RHO proteins (Figure S5). The Pro29/Gly30 tandem was previously proposed to endow the switch 1 of RAC1 with a unique flexibility,^39^ suggesting that it may underlie A41 specificity. To test this hypothesis, we substituted Gly30 of RAC1 with a Ser residue (RAC1 G30S), which mimics the sequence of CDC42. A41 (10^−5^M) was not able to inhibit TRIO-induced nucleotide exchange on the RAC1 G30S mutant (Figure 4C), indicating that the point mutation of Gly30 on RAC is sufficient to make it resistant to A41. This result suggests that the absence of Gly30 on CDC42 does not allow the switch 1 region to form a wide angle with the nucleotide pocket required for the A41 molecule to bind Lys16 (Figures 4A–4C). All these results further confirm the location of the binding site of A41 and identify this sequence in the switch 1 of RAC1 as a major determinant of A41 specificity.

### A41 inhibits the activity of oncogenic RAC1 variants in cells

Several RAC1 mutants have been involved in oncogenesis including RAC1 P29S, predominantly found in melanoma, which shows increased flexibility of switch 1,^40^ and RAC1b, a splice variant that carries an insertion downstream of switch 2.^41^ Both RAC1 P29S and RAC1b have been shown to spontaneously catalyze GDP/GTP exchange in the absence of GEF, which leads to uncontrolled RAC1 activation. In the case of RAC1b, crystal structures also suggested that the insert increases the flexibility of the switch 1.^42^ We thus analyzed the efficiency of A41 on these two oncomutants by pull-down assay in transfected cells. At 10^−5^M, A41 reduced the activity of both RAC P29S and RAC1b, to an extent similar to that of wild-type RAC1 (Figure 4D). Thus, A41 is efficient in limiting excess RAC1 activity, whether due to overactivation of wild-type RAC1 or to RAC1 mutations that increase its spontaneous activity. Overall, our results thus indicate that A41 possesses the pharmacological properties suitable for inhibiting RAC1 in the context of tumors.

### A41 inhibits RAC1 activity and oncogenic properties of TNBC cell line

**Figure 5:**
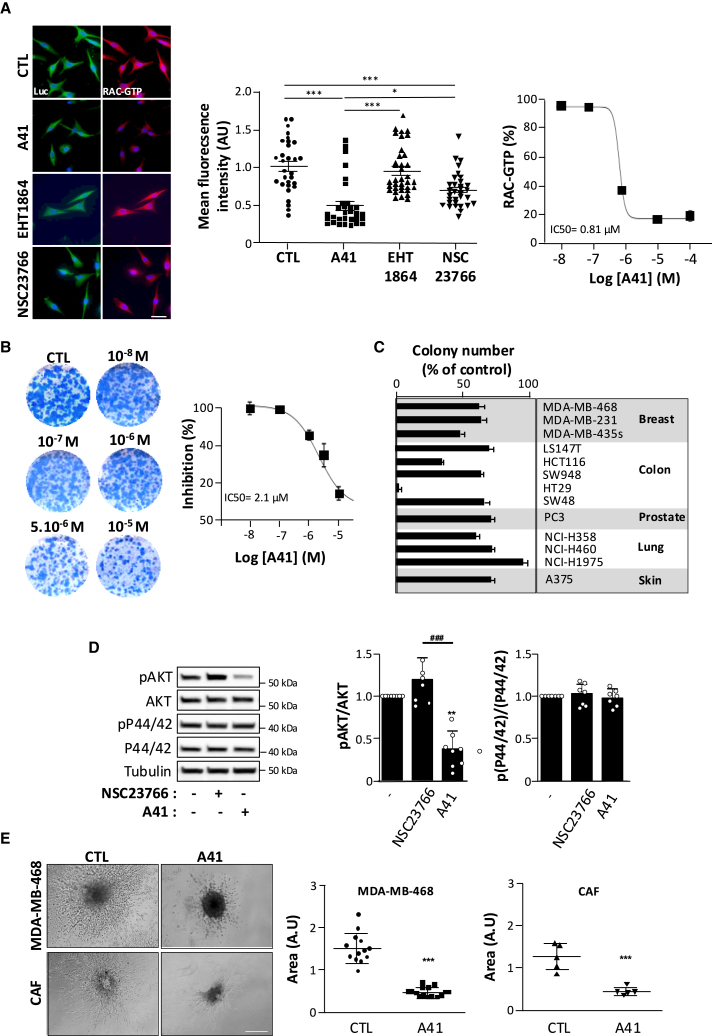
A41 decreases growth and invasiveness of TNBC cells *in vitro* (A) Representative images of RAC-GTP levels (red) in MDA-MB-468 Luc cells in the absence (CTL) and presence of EHT1864, NSC23766, or A41 (1 h at 10 μM) (left). Luciferase (Luc, green) was used to detect the cells. RAC-GTP mean fluorescence intensity was quantified inside the cell area (right) (*n* = 31–37 cells from 3 independent experiments; ∗*p* < 0.05, ∗∗∗*p* < 0.001) (scale bars: 10 μm). Dose-response curve for the inhibitory effect of A41 on RAC-GTP levels in MDA-MB-468 cells (*N* = 5 independent experiments). (B) Representative focus formation assay of MDA-MB-468 Luc cells in the absence (CTL) and presence of indicated concentration of A41 (left) and corresponding dose-response curve for the inhibitory effect of A41 on colony formation (right; *N* = 6 independent experiments). (C) Quantification of colony number of indicated cancer cell lines in the presence of A41 (10 μM) expressed as the percentage of control in the absence of A41. Results shown are representative of *N* > 3 independent experiments. (D) Immunoblot analysis and corresponding quantification of AKT and P44/42 expression and phosphorylation (pAKT, p44/42) in MDA-MB-468 Luc cells in the absence (−) and presence of NSC23766 or A41 (1 h at 10^−5^ M). Results shown are representative of 3 independent experiments. ∗∗*p* < 0.01 vs. untreated cells, ^###^*p* < 0.001 vs. NSC23766-treated cells. (E) 3D invasion assay of MDA-MB-468 Luc and cancer-associated fibroblasts (CAFs) and corresponding quantification (scale bars: 200 μm). Matrigel was polymerized in the absence (CTL) and in presence of A41 (10^−5^ M) (*N* = 3 independent experiments; ∗∗∗*p* < 0.001 vs. controls).

We next sought to characterize *in vitro* the biological effect of A41 in cancer cells by using the TNBC MDA-MB-468 luciferase (Luc) cell line (Figure 5). A41 (10^−5^M) decreased the active RAC1-GTP levels in MDA-MB-468 Luc cells by more than 50%, while at the same concentration, EHT1864 and NSC23766 have no or little effect on RAC1 activity, respectively (Figure 5A). Clonogenic assay revealed that incubation of MDA-MB-468 Luc cells with increasing concentrations of A41 significantly inhibited colony formation in a dose-dependent manner (Figure 5B). Inhibitory effect of A41 was also obtained on colony formation in other breast cancer cell lines and other types of cancer cell lines regardless of their oncogenic mutations (Figure 5C; Table S5). This suggests that intracellular downstream transducers of these oncogenic mutations are, at least in part, RAC-dependent processes. Indeed, hyperactivation of AKT, known to play a central role in the oncogenicity of a variety of mutations including *KRAS*, *PTEN*, and *PI3K*, is reduced by A41, while p44/42 activation is unchanged (Figure 5D). We next assessed the potential effect of A41 on MDA-MB-468 invasion in 3D collagen gel (Figure 5E). A41 (10^−5^M) reduced the migration area of MDA-MB-468 spheroids (Figure 5E). In the presence of A41, the size of the spheroids was not affected but appeared to be darker compared to untreated spheroids. These results suggest that A41 would not have an effect on cell survival but only on cell migration. In addition to the migratory capacity of cancer cells, tumor progression, invasion, and metastasis are critically dependent on cancer-associated fibroblasts (CAFs).^43^ Interestingly, the anti-invasion property of A41 seen on cancer cells was also observed in human breast CAF spheroids (Figure 5E). These results therefore suggest that A41 may have anti-metastatic properties.

### Compound A41 has suitable properties for *in vivo* testing

In order to assess the anti-tumor properties of A41 *in vivo*, we first would like to make sure of the safety and the absence of major off-target effects of A41. *In vitro* binding assays on a panel of potential targets, including membrane receptors, ion channel, and kinases, revealed only very limited off-target binding (Data S1 and Data S5). Conventional approaches that have been used to detect potential genotoxic effects of A41 (bacterial toxicity [Data S2], Ames fluctuations [Data S3], and micronucleus test [Data S4]) have excluded such effects in the concentration range 0.1–100 μM. *In vivo* safety has been addressed by monitoring various biological parameters in mice chronically receiving daily intraperitoneal A41 injection (1, 10, and 25 mg/kg) for 1 month. None of the parameters measured, including the weight of the mice, was affected by the chronic A41 treatment (Figure S6). No deaths were recorded, and visual inspection showed no sign of suffering or abnormal behavior of A41-treated mice. We then analyzed pharmacokinetics and tissue distribution of A41 following intraperitoneal injection of A41 (25 mg/kg). A41 rapidly reached its peak plasma concentration (∼15 min, 15.056 ± 0.5 μg/mL), which then declined over 2–3 h (Figure S7). A41 was detected in the liver, heart, and lungs. The highest concentration of A41 was found in the kidney, indicating renal uptake and subsequent clearance. A41 was found at extremely low concentration in the brain, which indicated that it could not cross the blood-brain barrier (Figure S7). Taken together, all these data suggest that A41 is suitable for *in vivo* testing in a mouse TNBC model.

### Compound A41 prevents metastasis in a mouse model of TNBC

**Figure 6:**
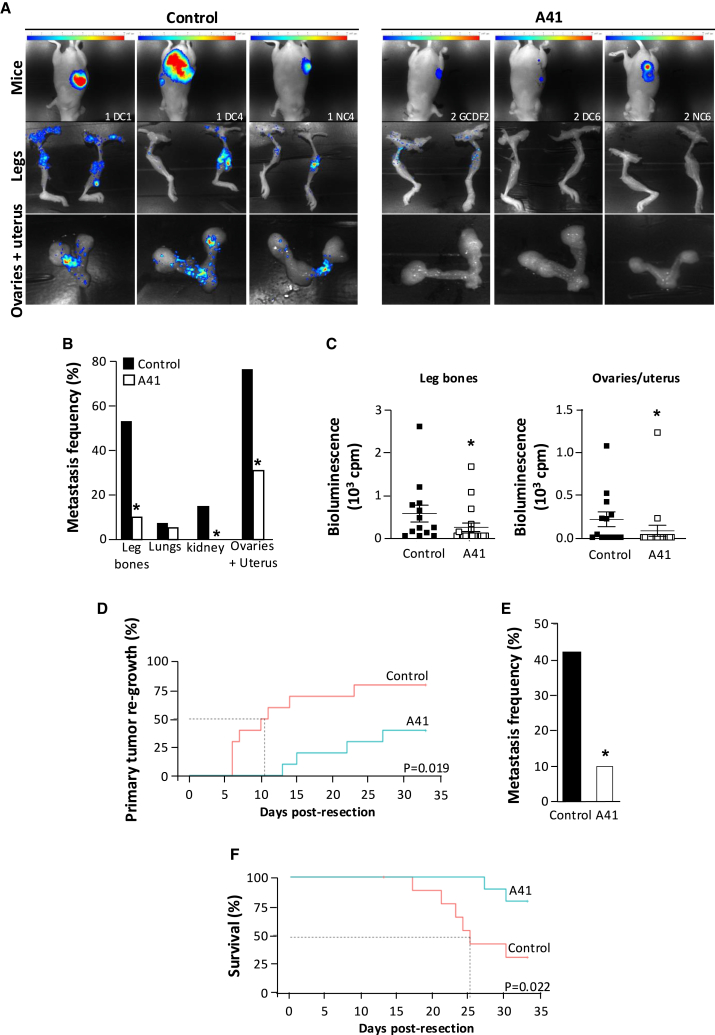
A41 decreases metastasis of TNBC cells *in vivo* (A) Representative images of luciferase bioluminescence intensities (BLI) in whole body, legs, and ovaries/uterus 4 weeks after resection of MD1-MB-468 Luc tumor in NMRI nude mice not treated (control) and treated with A41 (25 mg/kg/day, intraperitoneally for 4 weeks). (B) Metastasis frequency quantification based on *ex vivo* BLI measurements in indicated organs from control (black bars) and A41-treated mice 4 weeks after tumor resection (white bars) (*N* = 10–15 mice; ∗*p* < 0.05 vs. controls). (C) BLI quantification in legs and ovaries/uterus from control (black squares) and A41-treated (white squares) NMRI nude mice 4 weeks after tumor resection (*N* = 10–15 mice; ∗*p* < 0.05 vs. controls). (D) Cumulative proportion of immunocompetent control (red) and A41-treated mice (blue) displaying regrowth of the primary tumor after its resection. (E) Metastasis frequency quantified from *ex vivo* BLI measurements in leg bones, lungs, kidneys, and ovaries/uterus from immunocompetent control (black bar) and A41-treated (white bar) mice (*N* = 10 mice; ∗*p* < 0.05 vs. control). (F) Survival curve of immunocompetent control (red) and A41-treated mice (blue) after primary tumor resection (day 0).

The anti-metastatic potential of A41 was assessed in a TNBC xenograft model by orthotopic injection of MDA-MB-468 Luc cells into the mammary fat pad of 4-week-old Nordic Medical Research Institute (NMRI) nude mice randomized in 2 groups. The tumor grows gradually over time. At the 50^th^ day post grafting, when a volume of ∼1,000 mm^3^ was reached, the tumor was resected, which corresponded to tumor mass of approximately 0.6 g (Figure S8A). Mice were then treated with A41 (25 mg/kg/day, intraperitoneally; group 2) or vehicle for 4 weeks (group 1). Monitoring of the weight of the mice throughout the experiment, especially during the 4-week treatment period, shows that there was no difference between the two groups (Figure S8B). Longitudinal post-resection *in vivo* bioluminescence imaging (BLI) clearly showed a decrease in primary tumor regrowth in the A41 group compared to control mice (Figures 6A and S8C). *Ex vivo* BLI in relevant organs harvested at 4 weeks post resection did not detect metastasis in the liver and pancreas in both groups of mice. In contrast, it revealed the presence of secondary tumors in leg bones, lungs, kidneys, ovaries, and uterus with a significantly higher frequency in control than in A41-treated mice (Figure 6B). Moreover, *ex vivo* luminescence intensity of femur, ovaries, and uterus was significantly lower in A41-treated than in control mice (Figure 6C).

These results demonstrate that A41 limits tumor regrowth and metastases in a TNBC xenograft model in immunocompromised mice, which lacks the immune system component known to participate in the multi-step processes of tumor growth and dissemination.^44^ To circumvent this limitation and evaluate if the immune system could affect the anti-metastatic property of A41, we evaluated its effect in a syngeneic model. We injected murine 4T1 tumor cells into the mammary fat pad of immunocompetent BALB/c mice. A41 treatment after primary tumor resection significantly decreased the frequency of primary tumor re-growth, which was observed in 80% of control mice at 4 weeks post resection but in only 40% of A41-treated mice (Figure 6D), and also significantly reduced metastasis frequency (Figure 6E). These beneficial effects of chronic A41 treatment in immunocompetent mice were not accompanied by changes in white cell blood counts (Data S6), suggesting that A41 did not exhibit an immunosuppressive effect. Finally, treatment with A41 remarkably increased the survival rate from 30% in controls to 80% in A41-treated mice at 4 weeks post resection (Figure 6F).

### Compound A41 prevents metastasis in other invasive cancers

**Figure 7:**
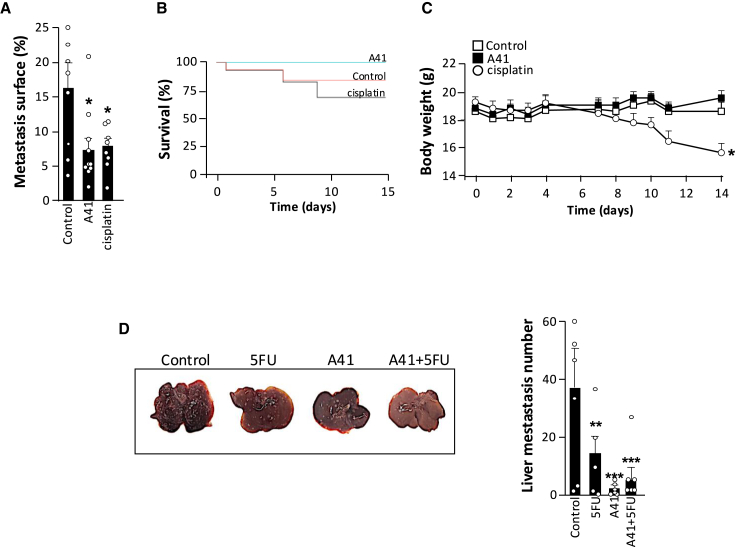
A41 decreases metastasis of colorectal cancer cells *in vivo* (A–C) Therapeutic effect of A41 on the pulmonary metastasis formation of colorectal circulating tumoral cells (CT26 cells). In control, A41-, and cisplatin-treated mice, metastasis surface (A), survival (B), and body weight (C) were quantified (*N* = 10 mice; ∗*p* < 0.05 vs. controls). (D) Therapeutic effect of A41 on colorectal tumor-dependent hepatic metastasis formation (*N* = 5–8 mice; ∗*p* < 0.05, ∗∗*p* < 0.01, and ∗∗∗*p* < 0.001 vs. controls).

To confirm the therapeutic potential of compound A41, we evaluated its anti-metastatic activity *in vivo* in other invasive cancer models. Following injection of colorectal tumor cells (CT26 cells) into the tail vein of mice, metastases were rapidly observed (Figure 7A). Compound A41 significantly reduced the development of pulmonary metastases with an efficacy comparable to the standard of care in colorectal cancer, the cisplatin. This anti-metastatic effect of A41 is accompanied by an improvement in animal survival (Figure 7B), in contrast to the toxicity of cisplatin when therapeutic effects are observed (Figures 7B and 7C).

Furthermore, in a mouse model of colorectal cancer development, we observed multiple liver metastases 2 weeks after subcutaneous implantation of CT26 cells (Figure 7D). Daily treatment of the mice with A41 significantly reduced the number of metastases and was more effective than 5-fluorouracil (5FU), another standard treatment for colorectal cancer.

Taken together, these results confirm that the therapeutic effects observed with A41 are not specific to breast cancer but could also be observed in other invasive cancers. Its efficacy would be comparable to the standard of care with the major advantage of no/less toxicity.

## Discussion

In this work, we identified the lead compound A41 as a selective inhibitor of RAC1. We show that A41 is a direct competitor of guanine nucleotides and that it occupies the nucleotide-binding site of RAC1 with high affinity and specificity. *In vitro*, A41 reduces the level of active wild-type RAC and of oncogenic RAC mutants, impairs RAC-dependent functions, and decreases invasiveness of TNBC cells. A41 displays good safety and bioavailability parameters *in vivo*. In a mouse model of TNBC, A41 decreased metastasis and increased survival. We discuss further the implications for the mechanism of action of A41 in cells and for its therapeutic use in cancer.

The nucleotide-binding site of small G proteins has long been considered undruggable for two major reasons: the high conservation of the nucleotide-binding site, which compromises specificity; and the extremely high affinity of GDP and GTP for the nucleotide-binding site combined with the high concentration of GTP in cells, which implies that inhibitors able to compete with guanine nucleotides must have an affinity in the same range.^45^ Our RAC1 inhibitor provides the proof of concept that the nucleotide-binding site of a wild-type small G protein is druggable by a non-covalent inhibitor. A41 functions by a mechanism distinct from that of NSC23766 and its derivatives and other previously described RAC inhibitors.^25^^,^^26^^,^^27^^,^^31^^,^^46^^,^^47^

We deciphered the mode of action of this inhibitor by demonstrating the interaction of A41 with Lys16 of RAC1. While A41 is specific to RAC proteins, this lysine residue is highly conserved in the nucleotide-binding pocket of RHO proteins. However, mutations in this region showed that Gly30, which is only present in the RAC protein sequences, plays a key role in the specificity of A41 for RAC proteins. We proposed earlier that Gly30, which is the only difference between RAC1 and RHOA (Glu30) or CDC42 (Ser30) in switch 1, endows switch 1 of RAC1 with a unique flexibility among the subfamily.^39^ Our observation that the Gly30Ser RAC1 mutant is insensitive to A41 suggests that the specificity of A41 for RAC1 arises from this structural dynamic of switch 1 allowing access to the nucleotide-binding site. More generally, the extensive interactions of A41 with the switch 1, a region that is highly variable between small G protein subfamilies, suggests that the inhibitor should readily distinguish between RAC1 and small G proteins outside the RHO protein subfamily.

Our results further provide the basis for the understanding of A41-mediated inhibition of RAC1 in cells. It is unlikely that A41 binds nucleotide-free RAC1 produced during the very slow spontaneous GDP/GTP exchange as we did not observe any change in nucleotide dissociation in the absence of GEF. We propose that in cells, A41 takes advantage of the nucleotide-free intermediate produced by GEF action to reach into the nucleotide-binding site.^37^ This highlights that transient intermediates of the exchange reaction may represent potent targets in drug discovery.

RAC1 is now well recognized as an important player in tumor initiation, progression, and metastatic dissemination and is also involved in immune escape and resistance to anti-tumor therapies.^3^^,^^20^ The aggressiveness of tumors and the poor prognosis associated with high RAC1 expression make targeting RAC1 in the clinical context an exciting therapeutic prospect.

Although RAC1 amplification is the predominant alteration of RAC1 in solid tumor that can lead to an increase in RAC1 expression and activity, the active RAC1 hotspot mutant RAC1 P29S has been identified in up to 9% of sun-exposed melanomas, making it the third most common gain-of-function mutation in melanoma.^48^ Other activating RAC1 mutants have also been found in other tumors or cancer cell lines such as RAC1 A159V mutant in head and neck neoplasms, RAC1 Q61R in primary prostate cancers,^40^^,^^49^ and RAC1 N92I in human sarcoma cell line HT1080.^50^ In addition, the naturally occurring splice isoform RAC1b is a self-activated fast-cycling variant involved in tumorigenesis and found to be overexpressed in colorectal and breast tumors.^41^^,^^51^ Considering the predicted interactions of A41 within the nucleotide-binding site, including the switch 1 but excluding the switch 2, we propose that oncogenic mutants that retain a native conformation of switch 1 should be sensitive to A41. Accordingly, we found that A41 significantly inhibits the overactivation of RAC1 P29S and RAC1b, both of which feature a native switch 1 conformation.

Overexpression or activation of some RAC GEFs such as VAV, TIAM, or DOCK is also responsible for upregulation of RAC1 signaling in cancers.^52^ Interestingly, our proposed mechanism for A41 binding to the transient nucleotide-free RAC1 intermediate of the GEF-induced nucleotide exchange reaction suggests that A41 may target nucleotide-free RAC1/GEF complexes regardless of the GEF. Thus, in addition to its ability to reduce the activity of wild-type RAC1 and cancer-associated RAC1 P29S and RAC1b, A41 may also be able to reduce a range of situations where RAC1 is upregulated by GEF mutations in cancers.

In solid tumors without RAC1 mutations, it would be useful to be able to determine the level of RAC1 activity to assess the potential oncogenic role of RAC1 overactivation and the potential value of its inhibition. Our results from the measurement of RAC1 activity in primary tumor by immunofluorescence suggest that high RAC1 activity could be predictive of metastasis relapses in breast cancer with a high specificity and sensitivity in TNBC. In addition to providing information on tumor aggressiveness, this measurement of RAC1 activity could be used as a predictive tumor biomarker to select patients whose tumors are characterized by high RAC1 activation and for whom the therapeutic use of an inhibitor of RAC1 activity would be beneficial.

*In vivo*, chronic administration of A41 significantly reduces the frequency of metastases in mouse models of TNBC and colorectal cancer. This effect is associated with an increase in the survival rate of treated mice, without observed adverse effects unlike standards of care for these cancers. This potent RAC inhibitor A41 can thus be a promising therapeutic agent to limit metastasis spreading in invasive cancers. Besides its direct beneficial effects, A41 could also be a valuable tool in the fight against acquired resistance to anti-cancer therapies, which remains a fundamental cause of relapses and treatment failure. The treatment of resistant tumors is a major challenge in the field of cancer therapy. It was recently observed that constitutive mutants of RAC1 or overactivity of the main RAC effector p21-activated kinase confer resistance to chemotherapy by mammalian target of rapamycin (mTOR) pathway activation and inhibition of apoptosis.^53^^,^^54^ In addition, a high-throughput functional screen using interference RNA to target genes commonly amplified in breast tumors with acquired resistance identified RAC1 as one of the most relevant proteins involved in the mechanisms of resistance. Remarkably, depletion of RAC1 expression restored sensitivity to chemotherapies.^55^ Thus, although not directly evaluated in the present study, it is likely that A41 may offer a pharmacological mean of preventing or correcting RAC1-mediated resistance to breast cancer therapies.

Overall, the pharmacological and safety properties of the RAC1 inhibitor A41 open up prospects for clinical trials aimed at assessing the risks and benefits of targeting RAC1 in human cancer, particularly in breast cancer. Based on the results described here, future therapeutic strategies should consider the use of RAC1 inhibitors in combination with existing anti-cancer therapies, to reduce tumor development and metastasis, as well as to decrease resistance to anti-cancer therapies. Furthermore, the correlation described between RAC1 overactivation and PD-L1 expression^56^^,^^57^ suggests that RAC1 inhibition in combination with anti-PD/PD-L1 (PD-L1, programmed death-ligand 1) immunotherapy or other agents that potentiate anti-tumor immune responses could represent a promising therapeutic strategy.

### Limitations of the study

This work has led to the identification of a RAC1-specific inhibitor. Although we have identified the mechanism of action of this small molecule A41, further development and optimization of this inhibitor is required to test it in the clinic. In addition, our study demonstrated the therapeutic efficacy of A41 in invasive cancers such as breast and colorectal cancer. It will be interesting to subsequently test the efficacy of this molecule in other types of cancer and in combination with different types of chemotherapy to determine the most interesting therapeutic combinations with A41.

## Resource availability

### Lead contact

Requests for further information and resources should be directed to and will be fulfilled by the lead contact, Dr. Vincent Sauzeau (vincent.sauzeau@univ-nantes.fr).

### Materials availability

All unique/stable reagents generated in this study are available from the lead contact with a completed materials transfer agreement.

### Data and code availability

• All data required to support the conclusions of this paper are included within the main text and supplementary materials.
• No original/custom code was generated in this study.
• Any additional information required to reanalyze the data reported in this paper is available from the lead contact upon request.

## Acknowledgments

The authors value the support provided by the animal facility units of Nantes University. This work also benefited from the support of the Labex IGO program funded by the ANR (ANR-11-LABX-0016-01). We thank the IBISA labeled and/or Biogenouest network member core facilities Therassay, M.shark, Imp@ct, and Cytocell (SFR François Bonamy, Nantes University) for molecular, functional, and cellular explorations. We acknowledge the MicroPICell core facility (SFR Bonamy, BioCore, Inserm UMS 016, CNRS UAR 3556, Nantes, France), member of the Scientific Interest Group (GIS) Biogenouest, IBISA, and the national infrastructure France-Bioimaging supported by the French National Research Agency (ANR-24-INBS-0005 FBI BIOGEN). Synthesis of RAC1 inhibitors used in this study was mostly performed by the IBISA core facility CHEM-Symbiose, as part of the Biogenouest network. This work was supported by grants from the Institut de Recherche en Santé Respiratoire des Pays de la Loire (STARac project), the TTO Ouest Valorisation (Oracle and Oracle2 projects), the French National Research Agency (ANR) (ORBIT project; ANR-22-CE18-0015), and the Institut National de la Santé et de la Recherche Médicale (INSERM). L.R. and D.H. were supported by grants from French Ministry of Higher Education and Research and from Fondation de la Recherche Médicale, respectively. C.T. was supported by a grant from region Pays de la Loire (PIRAMID) and doctoral school Biologie-Santé.

## Author contributions

Conceptualization, V.S.; methodology and validation, V.S., G.L., J.C., A.Q. M.M.M., and A.T.; investigation, F.D., L.R., A.Q., Y.F., D.H., M. Rousselle, H.B., S.M.L., C.T., G.A., M.M., M. Croyal, M. Riviere, F.S., A.P., A.T., and V.S.; writing – original draft, V.S., G.L., J.C., A.T., and F.D.; writing – review and editing, D.D., S.C., M. Croyal, E.M., P.J., and J.L.; funding acquisition, V.S.

## Declaration of interests

The authors have reported that they have no relationships with industry relevant to the contents of this paper to disclose. We, the authors, have a patent application related to this work: Patent WO2018224563: Inhibitors of RAC1 and uses thereof for treating cancers.

## STAR★Methods

### Key resources table

**Table:** 

REAGENT or RESOURCE	SOURCE	IDENTIFIER
**Antibodies**		

Anti-mouse IgG HRP-linked	Cell Signaling	CAT# 7076; RRID : AB_33144
Anti-rabbit IgG HRP-linked	Cell Signaling	CAT# 7074; RRID:AB_2099233
Phospho-Akt (Ser473)	Cell Signaling	CAT# 9271;RRID: AB_329825
Akt	Cell Signaling	CAT# 9272;RRID:AB_329827
Phospho-p44/42 MAPK (Erk 1/2) (Thy202/Tyr204)	Cell Signaling	CAT# 9101;RRID:AB_331646
p44/42 MAPK (Erk 1/2) (137F5)	Cell Signaling	CAT# 9271;RRID: AB_329825
P44/42	Cell Signaling	CAT# 4695, RRID:AB_390779
Anti-Firefly Luciferase	Abcam	CAT# ab21176;RRID:AB_446076
Active RAC1-GTP	New East Biosciences	CAT# 26903;RRID:AB_1961793
Anti-RAC1	BD Transduction Lab.	CAT# 610651;RRID:AB_397978
Anti-TUBA4A (TUBA1) Tubulin	Sigma-Aldrich	CAT# T9026;RRID:AB_477593
Goat anti-mouse IgG (H + L) Cross-Absorbed Secondary Antibody, Alexa Fluor 568	Invitrogen	CAT# A-11004;RRID:AB_2534072
Goat anti-rabbit IgG (H + L) Cross-Absorbed Secondary Antibody, Alexa Fluor 488	Invitrogen	CAT# A-11008;RRID:AB_143165
Anti-vimentin	Novus biologicals	CAT# NB300-223;RRID:AB_10003206
Anti-vimentin (5G3F10)	Cell Signaling	CAT# 3390;RRID:AB_2216128
Goat anti-chicken IgY, Alexa Fluor 488	Invitrogen	CAT# A-110339;RRID:AB_2534096
Anti-SM22a	Abcam	CAT# ab14106;RRID:AB_443021

**Biological samples**		

Breast cancer biopsies (human)	Institut de Cancérologie de l’Ouest (Nantes/Angers, FR)	

**Chemicals, peptides, and recombinant proteins**		

D-Luciferin K salt	Interchim	FP-M1224D
HitFinderTM collection	Maybridge	
DIVERSetTM-EXP	Chembridge	
DIVERSetTM-CL	Chembridge	
Recombinant Human EGF	Peprotech	AF-100-15
EHT1864	TOCRIS	3872
NSC 23766	TOCRIS	2161
5-Fluorouracil (5-FU)	Selleckchem	S1209
Cisplatin	Selleckchem	S1166
RAC1	Cytoskeleton	RH01
RHOA	Cytoskeleton	RC01
CDC42	Cytoskeleton	CD01
Phalloidin Alexa Fluor 488	Invitrogen	A12379
Firefly luciferase	Abcam	ab21176
DAPI	Thermo Scientific	62248

**Critical commercial assays**		

CYTOOplate™ 96 RW Custom A	CYTOO	20-950-00
E-Plate 96 well	Agilent	5232368001
ProteinWorks™ eXpress kit	Waters Corporation	

**Deposited data**		

Cancer Genome Atlas	Kmplot.com	

**Experimental models: Cell lines**		

NIH/3T3	ATCC	CRL-1658
MDA-MB-468	ATCC	HTB-132
MDA-MB-231	ATCC	HTB-26
MDA-MB-435s	ATCC	HTB-129
A375	ATCC	CRL-1619
4T1	ATCC	CRL-2539
CT26	ATCC	CCL-2638

**Experimental models: Organisms/strains**		

NMRI mice	Janvier Labs	
NMRI Nude mice	Janvier Labs	
C57Bl/6 mice	Charles River	

**Oligonucleotides**		

Primer forward for pET-3a-RAC1HisCter plasmid: CAATGCATTTCCTTCA GAATATATCCCTAC		
Primer reverse for pET-3a-RAC1HisCter plasmid: GTAGGGATATAT TCTGAAGGAAATGCATTG		

**Software and algorithms**		

GraphPad Software	Prism	
OSIRIS	Data Warrior software	
Accelrys Discovery Studio 4.0 software	DS4.0	
LigandFit and C-Docker programs	DS4.0	
ImageJ software	ImageJ	
MetaMorph software	MetaMorph	
MassLynx®	Waters Corporation	
MaxEnt_1_ extension software	Waters Corporation	
TargetLynx®	Waters Corporation	
ExPASy software	ExPASy	

**Other**		

Dmi6000b Wide Field Fluorescence Microscope	Leica Microsystems	
xCELLigence Cell Analyzer	Roche Applied Science	
*300* spectrometer	*Bruker Avance*	
*400* spectrometer	*Bruker Avance*	
DSQII quadripolar spectrometer	ThermoFinnigan	
LCQ Advantage spectrometer	ThermoFinnigan	
MAT95XL spectrometer	ThermoFinnigan	
LTQ-Orbitrap spectrometer	ThermoFisher Scientific	
Waters Atlantis T3 with an ELSD detector	Waters Corporation	
Xevo® TQD mass spectrometer with an electrospray (ESI)	Waters Corporation	
Acquity H-Class® UPLC™ device	Waters Corporation	
Synapt™ G2 HRMS Q-TOF mass spectrometer equipped with an ESI interface	Waters Corporation	
Imager IVIS spectrum bioluminescence imager	PerkinElmer	

### Experimental models and study participant details

#### Human biopsies

Breast cancer tumors were obtained after surgical resection at the Institut de Cancérologie de l’Ouest (Nantes/Angers, France). As required by the French Committee for the Protection of Human Subjects, informed consent was obtained from enrolled patients and protocol was approved by Ministère de la Recherche (agreement no.: DC-2012-1598) and by local ethic committee (agreement no.: CB 2012/06). Triple negative (TNBC) (*n* = 18) and Luminal B-like (LL) (*n* = 20) breast cancer patients were included to analyze RAC1 activity in biopsies.

#### Animals use

All experimental procedures and animal care were performed in accordance with the European Community Standards on the Care and Use of Laboratory Animals and approved by the local ethics committee (Comité d’Ethique en Expérimentation Animale des Pays de Loire) and conform to the ARRIVE guidelines.

The Animal Care Facility in Nantes is pathogen free and located in the *Unité de Thérapeutique Expérimentale* (UTE), which is accredited by the French Ministry of Agriculture (accreditation number C44-015). Mice are housed at 21°C with regulated relative humidity in isolated ventilated cages under positive pressure. A 12:12-h light-dark cycle is used and mice have free access to food (standard diet: *Safe* A04 irradiated at 10 kGy) and water (filtered water at 0.22 μm). The cage size is 500 cm^2^ and can include up to 5 mice according to the EU directives 2010/63/EC, poplar-based bedding (*Serlab* irradiated at 10 kGy) and enrichment (*Serlab* irradiated at 10 kGy) are used.

The animals were allocated to the experimental groups at random.

Experimental cancer models have been extensively studied in female mice.

By selecting a single sex, we reduced the complexity of our experimental design and we minimized the number of animals used in our study while still obtaining meaningful and interpretable results. (3Rs principle of Reduction). 5-week-old NMRI nude female mice were used for orthotopic breast cancer model. 7-8 weeks-old BALB/c female mice were used for circulating tumor cells model. 8 weeks-old BALB/c female mice were used for colorectal tumor model.

### Method details

#### In silico screening

The structure of RAC1 was first extracted from the crystal structure of RAC1-NSC23766 complex.^36^ Pharmacophore models were created from the binding site of NSC23766 with RAC1 using the Receptor-Ligand Pharmacophore Generation tools within Accelrys Discovery Studio 4.0 (DS4.0) software package. The pharmacophore models were built using HBA (hydrogen bond acceptor), HBD (hydrogen bond donor) and hydrophobic features. These features were created based on the observation of RAC1/NSC23766 interactions either directly from the ligand or in projection on RAC1 structure. The models consist of several combinations of four features, two main features (one HBA oriented toward the hydroxyl group of Ser71 and one HBD pointed to the O atom of Leu70), an accessory feature (one HBA oriented toward the amine group of Gln74) and several hydrophobic features facing residues Val36, Ala59, Tyr64, and leu67 of RAC1, completed by thirteen exclusion spheres centered on the main residues of the defined binding site (Val36, Asn39, Trp56, Asp57, Thr58, Ala59, Tyr64, Leu67, Arg68, Leu70, Ser71, Pro73 and Asn74).

The pharmacophore models were used as a search query against three dimensional multi-conformational molecular databases. The 2013 edition of the HitFinderTM collection (14,400 compounds) from Maybridge (www.maybridge.com), the DIVERSetTM-EXP (50,000 compounds) and the DIVERSetTM-CL (50,000 compounds) from Chembridge (www.chembridge.com) were used in the virtual screening. For the preparation of ligands, duplicate structures were removed and 3D coordinates were generated. A multi-conformational ligand database was then created using Catalyst within the Build 3D Database tool under DS4.0. The query was performed using the Search 3D Database tool with the FAST search method under DS4.0, retrieving as hits only compounds matching all features of the query.

The docking studies were performed using LigandFit option of receptor-ligand interactions protocol section available in DS4.0. Initially, RAC1 protein was prepared, by adding the hydrogen atoms and removing the water molecules, and then minimized using CHARMm force field. The protein molecule thus prepared was then defined as the total receptor. The ligand molecules retained by the pharmacophore models were docked into the binding site of the Rac1 and the interaction energies in the form of dock score^58^ between each ligand and the protein were calculated. Docking was performed using CFF as the energy grid. Penalty of 200kcal/mol/atom was set up to reduce the dock score of poses that occurred outside of the binding site. The conformational search of the ligand poses was performed by the Monte Carlo trial method. Maximum internal energy was set at 10000 kcal/mol. A short rigid body minimization was then performed (steepest descent and Broyden Fletcher Goldfarb Shanno (BFGS) minimizations). Ten poses were saved for each ligand after docking and 100 steps of BFGS rigid body minimization were then carried out. Scoring was performed with the scoring functions: LigScore1 and Ligscore2,^59^ using CFF force field. Best scored compounds were retained based on the calculation of a consensus score and binding free energies after *in situ* ligand minimization under DS4.0.

The potential binding mode of the hit A4 in the NSC23766 binding site as well as in the nucleotide binding pocket of RAC1 was predicted by additional docking experiments using the crystal structure of RAC1 extracted from RAC1/NSC23766 complex^36^ and the RAC1 structure extracted from RAC1/Arfaptin complex (PDB code 1I4D)^60^ respectively. Both LigandFit and C-Docker programs were used, the latter being another docking program using CHARMm-based molecular dynamics docking algorithm and implemented under DS4.0. For both docking programs, best poses among the 50 saved were retained based on consensus score (LigandFit) or C-Docker energy (C-Docker), and then compared based on the calculation of binding free energy after *in situ* ligand minimization.

#### Cell culture

NIH/3T3 cells grew up in DMEM (Gibco; Invitrogen) containing 1 g/L glucose, 10% fetal bovine serum, 100 units/mL penicillin and 100 μg/mL streptomycin at 37°C and 5% CO2. MDA-MB-468Luc, MDA-MB-231, MDA-MB-435s and A375 and primary culture of cancer associated fibroblasts^61^ cells grew up in DMEM (Gibco; Invitrogen) containing 4.5 g/L glucose, 10% fetal bovine serum, 100 units/mL penicillin and 100 μg/mL streptomycin at 37°C and 5% CO2. CT26 cells (chemically induced BALB/c mice-derived colorectal carcinoma) were cultured in DMEM media supplemented with 10% fetal bovine serum, penicillin (10 μg/mL), streptomycin (100 μg/mL) and L-glutamine (2 mM). Other cancer cell lines grew up in RPMI 1640 (Gibco; Invitrogen) containing 10% fetal bovine serum, 100 units/mL penicillin and 100 μg/mL streptomycin at 37°C and 5% CO2.

#### Cell imaging by immunofluorescence

After indicated treatments, cells were fixed with 4% paraformaldehyde and permeabilized in PBS 0.5% Triton X-100. To assess RAC1 activity, cells were then incubated with RAC-GTP antibody (26903, NewEast Biosciences, King of Prussia, Pa) (dilution 1/500) overnight at room temperature, followed by secondary Alexa 568-labeled anti-mouse antibody (dilution 1/1000). Cancer cells were detected with firefly luciferase antibody (ab21176, Abcam) (dilution 1/500) overnight at room temperature, followed by secondary Alexa 488-labeled anti-rabbit antibody (dilution 1/1000). To assess cytoskeleton organization, cells were incubated with Alexa Fluor 488 phalloidin (A12379, Invitrogen) to visualize F-actin. After staining, cells were mounted in Prolong gold antifade reagent with DAPI and images were captured by a fluorescence microscope.

#### Focus formation assays

1000 cells/well from the indicated cancer cell lines were seeded in 6-wells plates and allowed to grow 2 days before treatments. When indicated, cells were then treated three times a week with indicated doses or 10^−5^M (when not indicated) of A41. When the untreated well reached 70% of confluence, cells were fixed with 4% paraformaldehyde and colored with 0.1% Coomassie Blue. The area occupied by the cells was then quantified with ImageJ software.

#### Immunoblotting

After indicated treatments, MDA-MB-468Luc cells were incubated on ice with lysis buffer supplemented with proteases and phosphatases inhibitor cocktails (Sigma Aldrich, Saint Quentin Fallavier, France) and sodium orthovanadate. Lysates were subjected to SDS-PAGE, transferred to nitrocellulose membranes, and incubated with specific antibodies. P-Akt (9271), Akt (9272), pP44/42 (9101) and P44/42 (4695) antibodies were from Cell Signaling Technology (Leiden, The Netherlands). Tubulin was from Beckman Coulter (Villepinte, France). Vimentin was from Cell signaling (#3390). Immune complexes were detected with appropriate secondary antibodies and enhanced chemiluminescence reagent (Clarity ECL BioRad, Marnes la Coquette, France). Protein band intensities were quantified using ImageJ Software (NIH software, Bethesda, Md).

#### 3D invasion assays

3D-spheroids were obtained, according the protocol previously described,^62^ by re-suspending cells at a concentration of 1.25 × 10^4^ cells/ml in 20% Methyl-cellulose/80% DMEM 10% FBS. Then, 100 μL per well were distributed in 96-well-conical-plates (non-treated surface) and centrifuged 1 min at 200 × g. 3D-spheroids invasion was performed on spheroids containing 2500 cells in a collagen type I suspension (2 mg/mL) (BD/corning) (354249) supplemented with 1% FBS in presence or not of A41 (10^−5^M). Invasion was monitored by fully automated Dmi6000b Wide Field Fluorescence Microscope (Leica Microsystems, Wetzlar, Germany) during 48h.

#### RAC-GTP pull-down assays

Pull-down assay using GST-PBD fusion proteins were performed on NIH-3T3 lysates to assess RAC1 activity as previously described.^63^ The precipitated active RAC was subjected to SDS-PAGE and detected by immunoblot with anti-RAC1 antibody (BD biosciences) (dilution 1/1000).

#### Surface plasmon resonance studies

SPR immobilization was performed at 25°C. RAC1, RHOA and CDC42 purified proteins (respectively RH01, RC01 and CD01, Cytoskeleton) were diluted to 5 μg/mL in Na^+^ acetate buffer (pH 5.0) and injected into sensor chip CM5 (GE Healthcare) in a Biacore T200 (GE Healthcare) that was activated with NHS/EDC buffer. Approximately 5,000 response units of the purified protein were immobilized on the biosensor chip. Biosensor chips were blocked by an injection of 1 mM ethanolamine (pH 8.5). SPR analysis was performed at 25°C in HBSEP running buffer (5% DMSO) with or without EDTA (20 mM). A41 compound was injected over the RAC1, RHOA or CDC42 functionalized surface in concentration range from 0 to 25μM. Association and dissociation phases were monitored during 60s and 300s, respectively.

#### 1D cell migration

NIH3T3cells (1000/well) were seeded in a 96 well plate with 10mm fibronectin stripes (CytooPlates Motility, CYTOO) in medium with 1% SVF and allowed to spread for 4 h before capturing time-lapse images for 24 h (image/10 min) on a Widefield Leica DMI 6000B drove with Metamorph software. Cells speed was measured with ImageJ software.

#### Cell adhesion assay using impedance technology

NIH3T3cells (10000/well) were seeded in a 96 well plate microtiter xCELLigence assay plate (E-Plate) (ACEA Biosciences Inc.) and placed on the Real-time xCELLigence Cell Analyzer (Roche Applied Science) platform at 37°C to measure the “cell index” every 5 min for a period of 6 h. The cell index unit is defined as (Rn − Rb)/15. Rn is the cell electrode impedance of the well when it contains cells. Rb is the background impedance of the well with the media alone.

#### Proteins

Full-length RAC1 carrying a 6xHis tag in C-terminus was purified as previously described.^64^ Full-length RAC2, RHOG, RHOA and CDC42 were purified as previously described.^64^^,^^65^ Full-length RAC1G30S was obtained by directed mutagenesis on the pET-3a-RAC1HisCter plasmid using Quick change II site directed mutagenesis kit (Agilent technologies) accordingly to the manufacturer instructions (Primer for: CAATGCATTTCCTTCAGAATATATCCCTAC/Primer rev: GTAGGGATATATTCTGAAGGAAATGCATTG). Small GTPases were loaded with GDP prior to nucleotide exchange by incubation of 250 μM GTPase with 1.5 μM GDP and 10 μM EDTA for 30 min at room temperature. Nucleotide exchange was stopped by the addition of 20 mM MgCl2. Removal of excess nucleotides and buffer exchange was done by gel filtration. Trio^DH1PH1^ was purified as previously described.^39^ TIAM^DHPH^ is a kind gift of Scott Hansen (University of California, Berkeley). TIAM^DHPH^ was expressed in Rosetta (DE3) pLysS Escherichia coli strains in LB medium by induction with 0.5 mM IPTG overnight at 20°C. Bacterial pellet was resuspended in lysis buffer (20 mM Tris pH 8.0, 500 mM NaCl, 2 mM β-mercaptoethanol, 2 mM MgCl_2_, 10% glycerol, 0.5% tween 20, anti-protease cocktail) and frozen in liquid nitrogen. After thawing, benzonase was added to 7.5 U/mL and cells were disrupted using a French press, cleared by centrifugation at 14 000 g for 30 minutes and the supernatant was filtered over a 0.22 μm filter. Proteins were first purified by an affinity step using a 5 mL His-Trap column (GE Healthcare) with elution at 500 mM imidazole, followed by gel filtration on a Superdex 200 column (GE Healthcare) equilibrated with 20 mM Tris pH 8.0, 150 mM NaCl, 2 mM β-mercaptoethanol, 1 mM MgCl_2_, 5% glycerol. P115RHOGEF^DHPH^ (residues 388–799) was cloned into a pFastBac HTA vector (EcoRI-KpnI sites) for expression in insect cells. After sequencing, the plasmid pFastBac HTA was transposed into a bacmid by transformation into DH10Bac competent cells. After extraction and screening of the recombinant bacmids, Sf21 cells were transfected. After obtaining the viral stock, expression tests in 24-well plates were performed. P115RHOGEF^DHPH^ was expressed in insect cells and purified by affinity chromatography on a Histrap column followed by gel filtration with a Superdex 75 XK16/61 column (Amersham), and then concentrated at 30mg/ml in 50mM HEPES pH 7.4, 100mM NaCl, 2mM β-mercaptoethanol. Specific exchange activity toward RHOA determined by fluorescence kinetics is 0.04 s^−1^M^−1^.

#### Nucleotide exchange kinetics

Nucleotide exchange kinetics were measured by recording the increase in fluorescence following the association of mant-GTP on GDP-bound RHO protein or the decay in fluorescence following the dissociation of mant-GDP pre-loaded onto the RHO protein (λ_Ex_ = 360 nm, λ_Em_ = 440 nm) as previously described.^39^ All reactions were performed at 30°C in a buffer containing Tris 20 mM pH 8, NaCl 150 mM and MgCl_2_ 1 mM. The assay was performed with purified RHO protein at 0.5 μM, indicated GEFs at 0.01 μM, GTP at 1 μM, and A41 at 5 μM. K_obs_ and K_max_ were determined by a single exponential over the entire kinetics, which was preferred over analysis initial velocities which can be affected by the intrinsic fluorescence of chemical compounds as described here.^39^^,^^64^ All experiments were done at least in triplicate.

#### Histology

Paraformaldehyde (4% in PBS, 1 mL) was administered intratracheally in the lungs through a flexible catheter, trachea was ligatured, and lungs were excised. Lungs were fixed in 4% paraformaldehyde for 48 h and embedded into paraffin. Sections measuring 6 mm in size were stained with hematoxylin/eosin for morphological studies. Histological grade (/12 points) was determined to assess inflammation (0–8) and pulmonary remodeling (0–4). To assess smooth muscle hypertrophy/hyperplasia, sections were stained by immunochemistry with SM22α antibody (Abcam).

The biopsies paraffin-embedded sections (6 μm) were stained with hematoxylin/eosin for morphological studies. Sections were stained by immunochemistry with RAC-GTP (NewEast Biosciences, 26903) (dilution 1/500) and vimentin antibodies (Novusbio, NB300-223) (dilution 1/150).

#### Synthesis of chemical materials

*General considerations for the synthesis of RAC1 inhibitors:* Solvents were purified and dried by standard methods prior to use; alternatively, the MB SPS-800-dry solvent system was used to dry dichloromethane. Commercially available reagents were purchased from Sigma Aldrich and were used without purification. Dry dichloromethane was obtained by refluxing solvent on calcium hydride for an hour and distilled under argon. Glassware used for reaction was either flame dried under vacuum or under argon stream for several minutes. Reactions were carried out under rigorous anhydrous conditions and argon stream/positive pressure of argon. ^1^H and ^13^C NMR spectra were recorded on a *Bruker Avance 300* spectrometer fitted with a 5 mm i.d. BBO probe carefully tuned to the recording frequency of 300.13 MHz (for ^1^H) and 75.47 MHz (for ^13^C), the temperature of the probe was set at room temperature (around 293–294 K), on a *Bruker Avance 400* spectrometer fitted with a 5 mm i.d. BBFO+ probe carefully tuned to the recording frequency of 400.13 MHz (for ^1^H) and 100.61 MHz (for ^13^C) (Data S7). The spectra are referenced to the solvent in which they were run (7.26 ppm for ^1^H CDCl_3_ and 77.16 ppm for ^13^C CDCl_3_, 2.5 ppm for ^1^H DMSO and 39.52 ppm for ^13^C DMSO). Chemical shifts (*δ*) are given in ppm, and coupling constants (*J*) are given in Hz with the following splitting abbreviations: s = singlet, d = doublet, t = triplet, q = quartet, qt = quintet, sx = sextuplet, sp = septuplet, m = massif and br = broad. All assignments were confirmed with the aid of two-dimensional ^1^H, ^1^H (COSY), or ^1^H, ^13^C (HSQC, HMBC) experiments using standard pulse programs. All reactions were monitored by TLC on commercially available precoated plates (Kieselgel 60 F254), and the compounds were visualized with KMnO_4_ solution [KMnO_4_ (3 g), K_2_CO_3_ (20 g), NaOH (5% aq.; 5 mL), H_2_O (300 mL)] and heating or by UV (254 nm) when possible. Flash column chromatography was carried out using high purity grade (Merck grade 9385) pore size 60Å, 230-400 mesh particle size silica gel (Sigma Aldrich). Solvents used for chromatography were prior distilled on a Buchi rotavapor R-220-SE. Low resolution mass spectrometry (MS) were recorded on a ThermoFinnigan DSQII quadripolar spectrometer (coupled with a TracUltra GC apparatus) for Chemical Ionization (CI), on a ThermoFinnigan LCQ Advantage spectrometer for ElectroSpray Ionisation (ESI). High resolution mass spectrometry (HRMS) were recorded on a ThermoFinnigan MAT95XL spectrometer (for CI) and on a ThermoFisher Scientific LTQ-Orbitrap spectrometer (for ESI).

#### Analytical conditions

LC characterization was performed on a Waters Atlantis T3 (5μm, 4.6 x 150 mm; Waters) equiped with an ELSD detector. The chromatographic separation was carried out with the injection of 2μL of a sample solution (1mg/mL) followed by an isocratic elution (H2O/Methanol: 25/75) at a flow rate of 0.8 mL/min.

#### ADME-tox studies

Blood cell counts, kinase profiler**,** safety screen, bacterial cytotoxicity, Ames, micronucleus assays were performed by the company Eurofins (France). The kinase profiler was performed by the company Eurofins (Discovery, Panlab and Cerep).

#### *In vivo* pharmacokinetics

A41 was administrated intraperitoneally (25 mg/kg, aqueous solution of A41 in 5% DMSO, 50% PEG) in 2 months old C57Bl/6 after 4 h of fasting. After 5, 30, 60, 120 and 360 minutes following injection, mice (n = 3 *per* kinetic time) were sacrificed and tissues (blood, heart, lung, liver, kidney and brain) were collected. Control mice (n = 5) were used to get blank tissues for mass spectrometry analyses. Blood was collected by cardiac puncture into tubes containing 10% EDTA. Immediately after blood collection, plasma was separated by centrifugation for 5 min at 4 °C (2,000 × *g*). Solid tissues were rapidly excised and snap frozen in liquid nitrogen before to be disrupted and homogenized in PBS (1/10; wt/vol) using TissueLyser II (Quiagen). A41 concentrations were determined in mouse plasma and tissues by liquid chromatography-tandem mass spectrometry (LC-MS/MS). All solvents were LC-MS grade and purchased from Biosolve (Valkenswaard, Netherlands). A41 10× standard solutions were prepared and serially diluted in acetonitrile to obtain 8 standard solutions ranging 10-20,000 nmol/L. A41 10X standard solutions (5 μL or 20 μL) were added to blank plasma (45 μL) or blank tissue homogenates (180 μL), respectively, to get final concentrations ranging 1-2,000 nmol/L. Acetonitrile (5 μL or 20 μL) was added to plasma (45 μL) or tissue samples (180 μL). Tissue homogenates (standards and samples) were then centrifugated for 5 min at 5,000 × *g* for 5 min at 10 °C and supernatants (100 μL) were collected. Proteins were precipitated by the addition of 150 μL or 300 μL of acetonitrile containing labeled *D*_*3*_-A41 as internal standard (200 nmol/L) in all plasma (50 μL) and tissue supernatants (100 μL), respectively. After centrifugation (17,000 × *g*, 10 min, 10 °C), supernatants were collected and dried under a gentle stream of nitrogen. Dried samples were finally reconstituted with 25% acetonitrile containing 0.1% formic acid (100 μL), and injected into the LC-MS/MS system. Analyses were performed on a Xevo® TQD mass spectrometer with an electrospray (ESI) interface and an Acquity H-Class® UPLC™ device (Waters Corporation, Milford, MA, USA). Samples (10 μL) were injected onto a BEH C_18_ column (1.7 μm; 2.1 × 50 mm, Waters Corporation) held at 60 °C, and compounds were separated with a linear gradient of mobile phase B (acetonitrile, 0.1% formic acid) in mobile phase A (water, 0.1% formic acid) at a flow rate of 500 μL/min. Mobile phase B was kept constant for 0.5 min at 1%, linearly increased from 1% to 100% for 3 min, kept constant for 0.5 min, returned to the initial condition over 0.5 min, and kept constant for 0.5 min before the next injection. A41 and its internal standard *d*_*3*_-A41 were then detected by the mass spectrometer with the ESI interface operating in the positive ion mode (capillary voltage, 3 kV; desolvatation gas (N_2_) flow and temperature, 900 L/h and 450 °C; source temperature, 120 °C). The multiple reaction monitoring mode was applied for MS/MS detection at the following mass-to-charge (*m/z*) ratio transitions: 487.1 → 153.0 and 490.2 → 156.0 for A41 and *d*_*3*_-A41, respectively. Cone voltage and collision energy were set at 35 V and 20 eV, respectively. Data acquisition and processing were achieved using MassLynx® and TargetLynx® softwares (version 4.1, Waters Corporation). Chromatographic peak area ratios between A41 and its internal standard constituted the detector responses. Standard solutions were used to plot calibration curves for quantification and dilution factors related to biological matrix preparations were included in calculation. The linearity was expressed by the mean r^2^ which was greater than 0.995 for all matrices (linear regression, 1/x weighting, origin excluded). AUC_0-6h_ was calculated as follow:

$${AUC}_{0-6h}=\int_{0}^{6h}C_{0}e^{-Kt}dt=C_{0}/K$$

Where K = constant of elimination, and C_0_ = administrated dose.

#### RAC1 labeling by irradiation and photoaffinity

Solutions of RAC1 (2.1 mg/mL) in PBS containing EDTA (84 μg/mL) were irradiated with or without [N3]-A41 (500 μg/mL) at 362 nm for 6 min. A control solution of RAC1 (no irradiation and without [N3]-A41) was used to ascertain the stability of the protein throughout the experiment. Prior all experiments, both modified and unmodified RAC1 samples (∼100 μL) were desalted and concentrated with 3 mL of 50 mM ammonium bicarbonate (Sigma Aldrich) buffer (pH 8) and a 5-kDa molecular weight cut-off filter. Resulting samples were stored at -20 °C until analysis.

*Analysis of whole proteins*. Samples were directly analyzed by liquid chromatography-high resolution mass spectrometry (LC-HRMS). LC-HRMS analyses were performed on a Synapt™ G2 HRMS Q-TOF mass spectrometer equipped with an ESI interface operating in the positive mode and an Acquity H-Class® UPLC™ device (Waters Corporation). Samples were injected (10 μL) onto a Acquity® CSH C18 (1.7 μm; 2.1 × 150 mm; 180 Å) reversed-phased LC column held at 60 °C. Proteins were then eluted over 20 min with a linear gradient of mobile phase B (100% acetonitrile) in mobile phase A (5% acetonitrile), each containing 0.1% formic acid, and at a flow rate of 250 μL/min. Mobile phase B was kept constant at 1% for 1 min, then linearly increased from 1% to 80% for 15 min, kept constant at 80% for 1 min, returned to the initial condition over 1 min, and kept constant for 2 min before the next injection. The full-HRMS mode was applied for protein detection (*m/z* range 100-4,000) at a mass resolution of 25,000 full-widths at half maximum. The ionization settings were as follows: capillary voltage, +3 kV; cone voltage, 30 V; desolvation gas (N_2_) flow rate, 900 L/h; desolvation gas/source temperatures, 450/120 °C. Leucine enkephalin solution (2 μg/mL, 50% acetonitrile) was infused at a constant flow rate of 10 μL/min in the lockspray channel, allowing for correction of the measured *m/z* throughout the batch (theoretical *m/z* 556.2771 in positive mode). Data acquisition and processing were achieved using MassLynx® software (version 4.1, Waters Corporation). Complex mass spectra under chromatographic peaks were deconvoluted with the MaxEnt_1_ extension software to get the experimental molecular weights of proteins, which were then compared with those of the unmodified protein to estimate the number of added A469 residues.

*Peptide mapping after RAC1 tryptic digestion*. The positive ESI mode is appropriate for proteins (ESI+) but leads to the formation of several adducts (H+, K+, Na+…) with multiple charge states ([M+nH]^n+^ ions) in addition to natural isotope signals (^13^C, ^2^H, ^15^N…). Protein samples were therefore subjected to proteolysis in order to form peptides that are more easily detected by mass spectrometry (lower charge states) and suitable for tandem mass spectrometry (MS/MS) analysis. The protein samples (50 μL) were reduced (addition of 120 μL ammonium bicarbonate 50 mM containing 7 mg/mL of RapidGest detergent [Waters Corporation], incubated 10 min at 80 °C; then addition of dithiothreitol, 70 mM, 20 μL, incubated 20 min at 60 °C), alkylated (addition of iodoacetamide, 142 mM, 30 μL, incubated 20 min at room temperature in the dark) and trypsin digested overnight (7 mg/mL in HCl 1 mM, 30 μL, 37 °C) using the ready-to-use solutions of the ProteinWorks™ eXpress kit (Waters Corporation) and according to the manufacturer’s instructions. Enzymatic digestion was stopped with 20% trifluoroacetic acid (TFA; 5 μL). After 15 min at 45 °C, the precipitate was removed by centrifugation (15 min, 10°C, 10,000 × *g*), and supernatants were removed for LC-HRMS and LC-MS/MS analyses. Proteotypic peptides were separated and detected by the full-HRMS mode as described above. In parallel, protein sequence was *in-silico* digested using the free Expasy software (https://web.expasy.org/peptide_mass). All tryptic peptides were looked for from their theoretical *m/z* ratios assuming several charged ions (from 1+ to 6+). Relevant peptides were then subjected to MS/MS fragmentations to ascertain their amino-acid sequences. Then, peptide sequences were compared with each other and between both modified and unmodified protein samples to establish the location of the labeled A469 (+485.1 Da). Finally, MS/MS fragmentation patterns allowed the identification of the modified amino-acid during the labeling experiment.

#### Orthotopic breast cancer model

Orthotopic cell xenograft was performed by injection of 4 million MDA-MB-468Luc cells in the fourth fat pad from 5-week-old NMRI nude mice. Primary tumour was allowed to develop during 5 weeks and tumour growth was assessed by tumour volume measurement using a caliper. Primary tumor was then removed by surgical exeresis and weighed. Treatments began 1 day before exeresis. A41 (25 mg/kg) was administered intraperitoneally on a daily basis during 4 weeks. Mice were weighed 5 times a week and *in vivo* bioluminescence intensities were measured once a week (Imager PerkinElmer IVIS spectrum bioluminescence imager). At the end of the protocol, mice were sacrificed and *ex vivo* bioluminescence intensities of indicated organs (legs, ovaries, uterus, lungs and liver) were measured within 15 min of D-luciferin intraperitoneal injection (150 mg kg−1). Photons emitted by cancer cells were counted by bioluminescent imaging and expressed in counts per minute (c.p.m.).

#### Colorectal cancer model

##### Circulating tumor cells

7-8 weeks-old BALB/c female mice received an intravenous injection through the caudal vein of the cell suspension (CT26 cells, ATCC® CCL-2638™). The volume needed for implantation was loaded in insulin syringes (2x10^5^ cells) in 100 μL of phosphate-buffered saline). The anesthetized mice were placed on a warming blanket and were monitored (breathing) until they wake up. A41 (25 mg/kg BW) was administered (i.p) daily from day 0 to day 14 and Cisplatin (3 mg/kg BW) was administrated (i.p) at days 4, 8, and 12.

##### Tumor model

8 weeks-old BALB/c female mice were obtained from the School of Veterinary Sciences at the National University of La Plata and treated in accordance with the Canadian Council on Animal Care and ARRIVE guidelines. 1x10^6^ CT26 viable cells were resuspended in PBS (100 μL) and injected subcutaneously into the right flank of each animal. Mice were distributed and treated as follows: control, consist in a daily intraperitoneal (ip) administration of vehicle (5% DMSO, 45% PEG400); A41, ip administration of 25 mg/kg BW/day; 5-FU, ip administration 20 mg/kgBW/week in sterile water; A41+5-FU, ip administration of A41 and 5-FU treatments. Liver metastases were identified and quantified under a stereoscopic magnifying glass. These lesions were distinguished by their isolated circular shape, their marked whitish color contrasting with the reddish liver tissue, and their raised appearance.

### Quantification and statistical analysis

All data are expressed as the mean ± SEM of sample size n. For multiple comparisons, the non-parametric Kruskal-Wallis test was used followed by Dunns’ post-test. When the sample size was greater than 30, the one-way ANOVA test was used followed by Tukey’s multiple comparisons test. For individual comparisons, statistical analysis was performed using non-parametric t-test (Mann-Whitney). Data analysis was performed using the GraphPad Prism software. The threshold for statistical significance was set at *p* < 0.05.

## Supplemental information

Data S1. Data S1–S8

Document S2. Article plus supplemental information
