# Supplementary material for: A Rac-specific competitive inhibitor of guanine nucleotide binding reduces metastasis in triple-negative breast cancer
Source: Cell Rep Med. 2025 Jul 8;6(7):102233. doi: 10.1016/j.xcrm.2025.102233 (PMC12281424; doi:10.1016/j.xcrm.2025.102233)
Supplement: Document S1. Figures S1–S8 and Tables S1–S5 [file mmc1.pdf]

Cell Reports Medicine, Volume *6*

Supplemental information

A Rac-specific competitive inhibitor of guanine nucleotide binding reduces metastasis in triple-negative breast cancer

Florian Dilasser, Lindsay Rose, Agnès Quemener, Yann Ferrandez, Dorian Hassoun, Morgane Rousselle, Hugo Bergereau, Séverine Marionneau Lambot, Luciano E. Anselmino, Camille Trouillet, Gwennan Andre, Mike Maillasson, Mikael Croyal, Matthieu Riviere, Didier Dubreuil, Sylvain Collet, Frédérique Souaze, Mario Campone, Anne Patsouris, Erwan Mortier, Mauricio Menacho Marquez, Philippe Juin, Jacques Lebreton, Arnaud Tessier, Jacqueline Cherfils, Gervaise Loirand, and Vincent Sauzeau

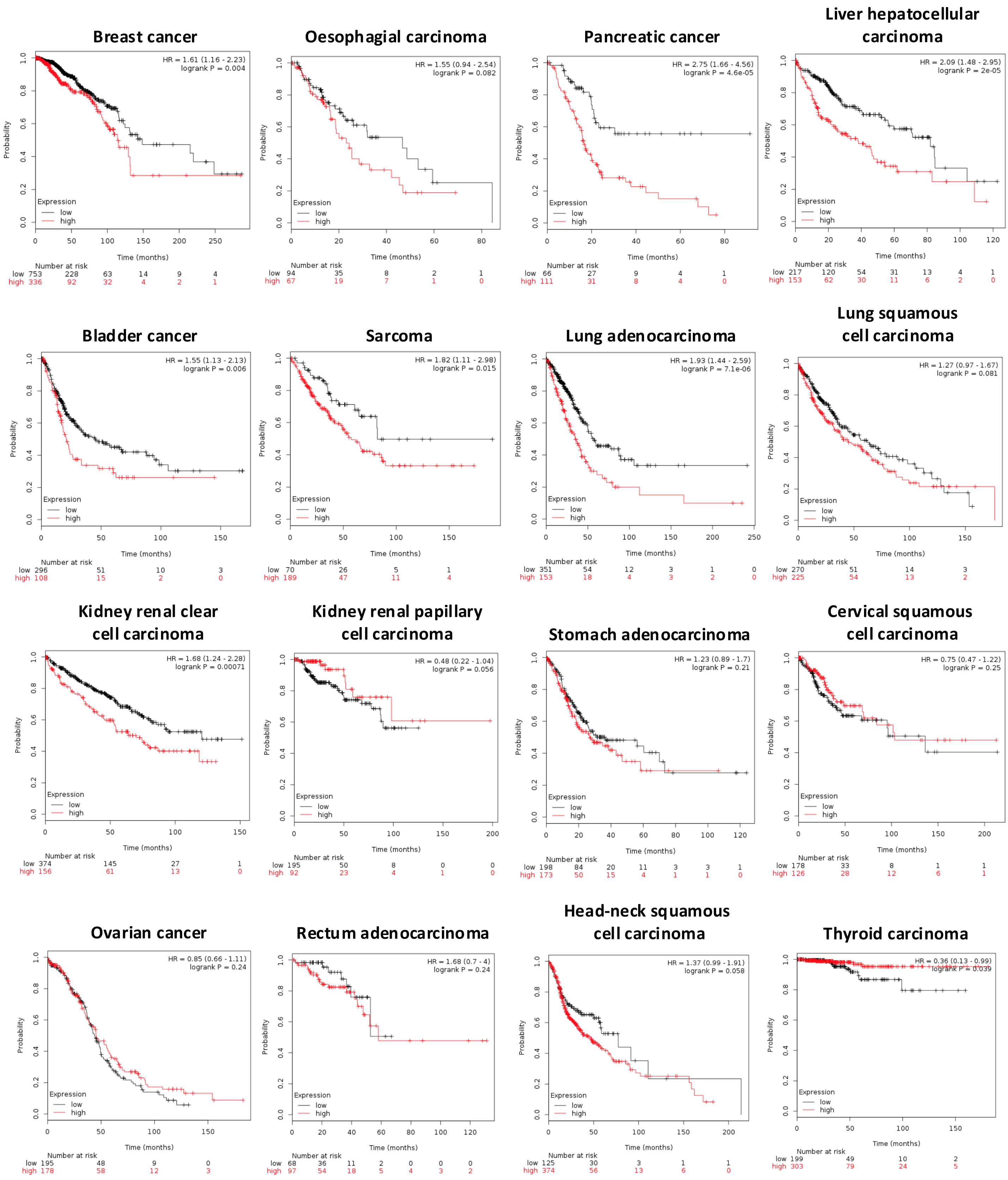
Breast cancer
HR = 1.61 (1.16 - 2.23)
logrank P = 0.004
low 753 228 63 14 9 4
high 336 92 32 4 2 1
Oesophagial carcinoma
HR = 1.55 (0.94 - 2.54)
logrank P = 0.082
low 94 35 8 2 1
high 67 19 7 1 0
Pancreatic cancer
HR = 2.75 (1.66 - 4.56)
logrank P = 4.6e-05
low 66 27 9 4 1
high 111 31 8 4 0
Liver hepatocellular carcinoma
HR = 2.09 (1.48 - 2.95)
logrank P = 2e-05
low 217 120 54 31 13 4 1
high 153 62 30 11 6 2 0
Bladder cancer
HR = 1.55 (1.13 - 2.13)
logrank P = 0.006
low 296 51 10 3
high 108 15 2 0
Sarcoma
HR = 1.82 (1.11 - 2.98)
logrank P = 0.015
low 70 26 5 1
high 189 47 11 4
Lung adenocarcinoma
HR = 1.93 (1.44 - 2.59)
logrank P = 7.1e-06
low 351 54 12 3 1 0
high 153 18 4 3 2 0
Lung squamous cell carcinoma
HR = 1.27 (0.97 - 1.67)
logrank P = 0.081
low 270 51 14 3
high 225 54 13 2
Kidney renal clear cell carcinoma
HR = 1.68 (1.24 - 2.28)
logrank P = 0.00071
low 374 145 27 1
high 156 61 13 0
Kidney renal papillary cell carcinoma
HR = 0.48 (0.22 - 1.04)
logrank P = 0.056
low 195 50 8 0 0
high 92 23 4 1 0
Stomach adenocarcinoma
HR = 1.23 (0.89 - 1.7)
logrank P = 0.21
low 198 84 20 11 3 3 1
high 173 50 15 4 1 1 0
Cervical squamous cell carcinoma
HR = 0.75 (0.47 - 1.22)
logrank P = 0.25
low 178 33 8 1 1
high 126 28 12 6 1
Ovarian cancer
HR = 0.85 (0.66 - 1.11)
logrank P = 0.24
low 195 48 9 0
high 178 58 12 3
Rectum adenocarcinoma
HR = 1.68 (0.7 - 4)
logrank P = 0.24
low 68 36 11 2 0 0 0
high 97 54 18 5 4 3 2
Head-neck squamous cell carcinoma
HR = 1.37 (0.99 - 1.91)
logrank P = 0.058
low 125 30 3 1 1
high 374 56 13 6 0
Thyroid carcinoma
HR = 0.36 (0.13 - 0.99)
logrank P = 0.039
low 199 49 10 2
high 303 79 24 5
Probability
Time (months)
Number at risk
Expression
low
high

	TNBC (N=18)	LL (N=20)
**Histologic.subtype**		
Poorly differenciated	16/18	16/20
Micropapillary	0/18	1/20
Colloïdal 20%	0/18	1/20
Non specified	2/18	2/20
**Status**		
Remission	10/18	10/20
Recurrence	8/18	10/20
**Age.Dg (years)**		
Median	63.000	60.500
Q1, Q3	52.0, 72.2	47.7, 65.5
**Laterality**		
Left	12/18	9/20
Right	6/18	11/20
**Neoadjuvant.Chemotherapy**		
NO	17/18	20/20
YES	1/18	0/20
**Breast.Surgery**		
Conservative	13/18	15/20
Mastectomy	5/18	5/20
**LymphNode.Surgery**		
GAS	13/18	4/20
CURAGE	5/18	16/20
**Stade.UICC**		
I	10/18	5/20
IIA	3/18	6/20
IIB	1/18	2/20
IIIA	2/18	5/20
IIIB	1/18	1/20
IIIC	1/18	1/20
**Histologic.Grade**		
-1	1/18	2/20
-2	5/18	5/20
-3	12/18	13/20
**CIS**		
NO	8/18	8/20
YES	10/18	12/20
**Embols**		
NO	13/18	6/20
YES	5/18	14/20
**Lymphocytic.infiltration**		
NO	14/18	20/20
YES	4/18	0/20
**RE.H**		
Negative	17/18	0/20
Positive	1/18	20/20
**RP.H**		
Negative	18/18	1/20
Positive	0/18	19/20

	TNBC (N=18)	LL (N=20)
**Radiotherapy.Breast.ChestWall**		
NO	2/18	0/20
YES	16/18	20/20
**Radiotherapy.LymphNodes.area**		
NO	15/18	9/20
YES	3/18	11/20
**Chemotherapy.Type**		
NO	3/18	3/20
ANTHRACYCLINE	2/18	6/20
TAXANE	1/18	0/20
ANTHRACYCLINE + TAXANE	11/18	11/20
ANTHRACYCLINE + TAXANE + BEVACIZUMAB	1/18	0/20
**Hormonotherapy.Adjuvant**		
NO	17/18	0/20
YES	1/18	20/20
**Tumoral emboli**		
NO	13/18	6/20
YES	5/18	14/20

Supp Table 1. Clinicopathologic data of TNBC and Luminal B-like (LL) patients included in the analyze of RAC1 activity.

			Druglikeness					
Name	Structure	Molecular Formula	MW	logP	HBD	HBA	Docking score	IC_{50} (nM)
A4	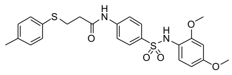	$C_{24}H_{26}N_2O_5S_2$	486.60	3.977	2	6	-10.32	34.2
**A41**	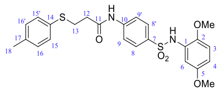	$\mathbf{C_{24}H_{26}N_2O_5S_2}$	**486.60**	**3.977**	**2**	**6**	**-11.12**	**2.5**
A414	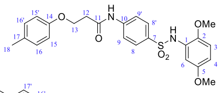	$C_{24}H_{26}N_2O_6S$	470.54	3.339	2	6	-11.62	0.56
A416	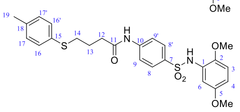	$C_{25}H_{28}N_2O_5S_2$	500.63	4.432	2	6	-9.92	2600
A415	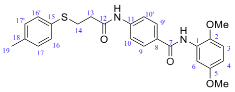	$C_{25}H_{26}N_2O_4S$	450.55	4.826	2	5	-7.53	-

Supp. Table 2. Chemical library derived from A4 compound. For each molecule, druglikeness has been assessed according to Lipinski's rules: molecular weight (MW)<500; high lipophilicity (LogP<5); less than 5 hydrogen bond donors (HBD); less than 10 hydrogen bond acceptors (HBA). The log P was calculated by using OSIRIS – Data Warrior software. The docking score (kcal/mol) for the nucleotide binding site corresponds to the value of the binding free energy after *in situ* ligand minimization divided by the number of heavy atoms of each ligand. IC_{50} was determined experimentally from the inhibition of ruffles formation.

			Druglikeness					
**Name**	**Structure**	**Molecular Formula**	**MW**	**logP**	**HBD**	**HBA**	**Docking score**	**Inhbition (%)**
**A41**	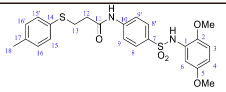	$C_{24}H_{26}N_2O_5S_2$	486.60	3.977	2	6	-11.12	46
**[N_3]- A41**	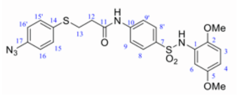	$C_{24}H_{24}N_2O_5S_2{}^2H_3$	513.59	3.905	2	7	-11.08	46

Supp. Table 3. Chemical property of [N3]- A41 compound. For each molecule, druglikeness has been assessed according to Lipinski's rules: molecular weight (MW)<500; high lipophilicity (LogP<5); less than 5 hydrogen bond donors (HBD); less than 10 hydrogen bond acceptors (HBA). The docking score (kcal/mol) for the nucleotide binding site corresponds to the value of the binding free energy after *in situ* ligand minimization divided by the number of heavy atoms of each ligand. Inhibition of RAC1 activity was determined experimentally from the inhibition of nucleotide exchange assay.

Type	Sequence<br>MS/MS fragments	Fragment ion (*m/z*)<br>Precursor: *m/z* 530.8	Fragment ion (*m/z*)<br>Precursor: *m/z* 773.4	Mass shift<br>A469: 485 Da
y_{10}^{+}	VVVGDGAVGK	900.8	-	-
y_{9}^{+}	VVGDGAVGK	801.7	1286.6	Yes
y_{8}^{+}	VGDGAVGK	702.6	1187.6	Yes
y_{7}^{+}	GDGAVGK	603.5	-	-
y_{6}^{+}	DGAVGK	546.5	1031.6	Yes
y_{5}^{+}	GAVGK	431.4	916.5	Yes
y_{4}^{+}	AVGK	374.5	-	-
y_{3}^{+}	VGK	303.3	788.5	Yes
**y_{2}^{+}**	**GK**	**204.2**	**689.5**	**Yes**
y_{1}^{+}	K	-	-	-
b_{1}^{+}	C	-	-	-
b_{2}^{+}	CV	260.2	260.2	No
b_{3}^{+}	CVV	-	-	-
b_{4}^{+}	CVVV	458.3	458.3	No
b_{5}^{+}	CVVVG	515.4	515.4	No
b_{6}^{+}	CVVVGD	-	-	-
b_{7}^{+}	CVVVGDG	-	-	-
b_{8}^{+}	CVVVGDGA	758	758	No
b_{9}^{+}	CVVVGDGAV	-	-	-
**b_{10}^{+}**	**CVVVGDGAVG**	**914**	**914**	**No**

Supp Table 4. Fragmentation patterns of unlabeled (precursor: *m/z* 530.8) and labeled (precursor: *m/z* 773.4) CVVVGDGAVGK peptide.

**Tissue**	**Cell line**	**Mutation**
Breast	MDA-MB-468	*P53*
	MDA-MB-231	*KRAS, BRAF*
	MDA-MB-435s	*BRAF*
Colon	LS147T	*KRAS, PI3K*
	HCT 116	*KRAS, PI3K*
	SW948	*PI3K*
	HT29	*BRAF*
	SW48	NA
Prostate	PC3	*PTEN*
Lung	NCI-H358	*KRAS*
	NCI-H460	*KRAS, PI3K*
	NCI-H1975	*PI3K*
Skin	A375	*BRAF*

Supp Table 5. Known mutations of cancer cell lines from various organs and tissues used in clonogenic assays.

Supplementary. Fig. 1: RAC1 activity as poor prognostic factor of aggressive cancer. Kaplan-Meier survival curves for patients with low (black line) or high (red line) RAC mRNA in the indicated cancer types. Data used were obtained with the Cancer Genome Atlas (Kmplot.com)

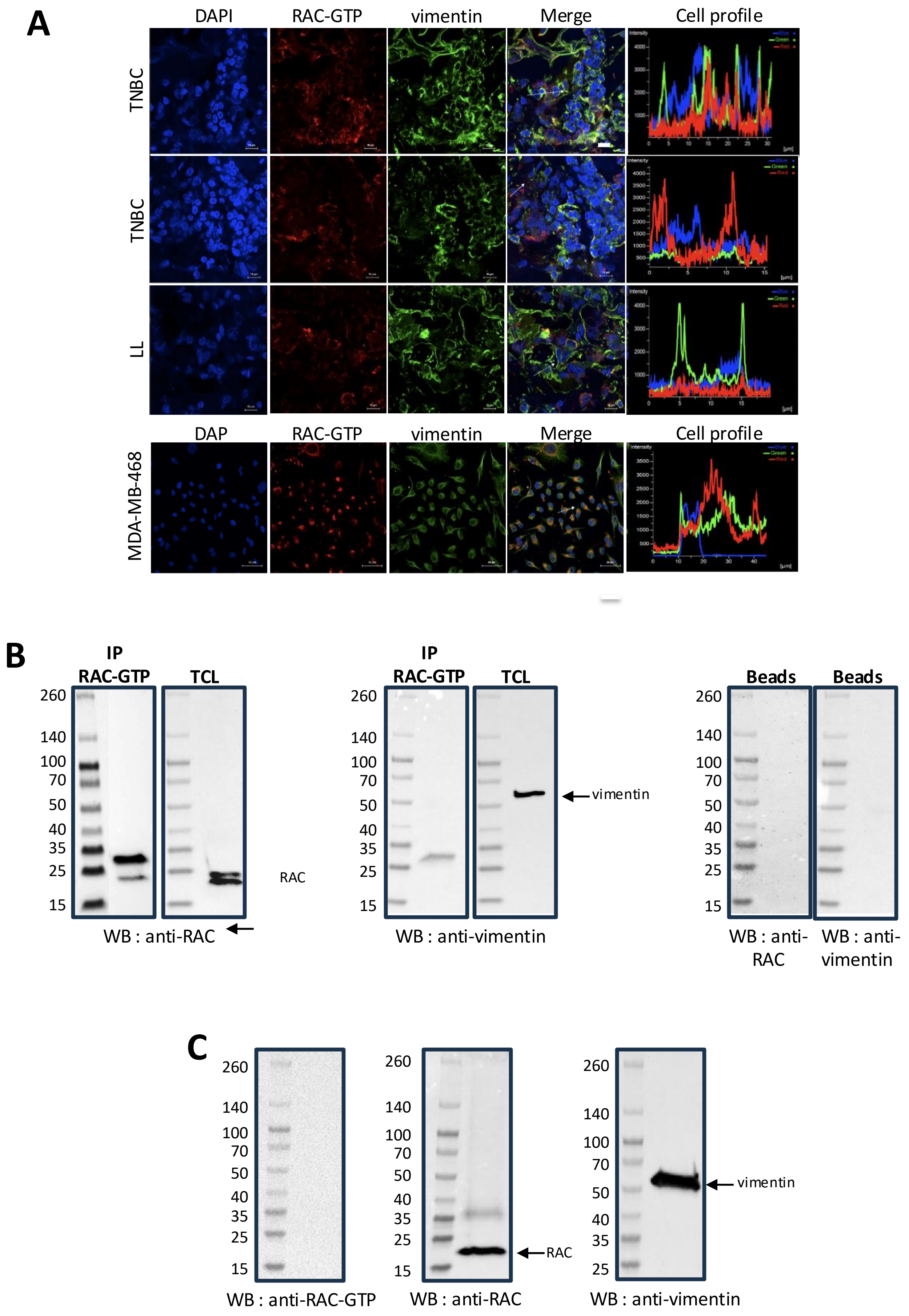
A
DAPI
RAC-GTP
vimentin
Merge
Cell profile
TNBC
TNBC
LL
DAP
RAC-GTP
vimentin
Merge
Cell profile
MDA-MB-468
B
IP
RAC-GTP
TCL
260
140
100
70
50
40
35
25
15
RAC
WB : anti-RAC
IP
RAC-GTP
TCL
vimentin
WB : anti-vimentin
Beads
Beads
WB : anti-RAC
WB : anti-vimentin
C
260
140
100
70
50
40
35
25
15
WB : anti-RAC-GTP
RAC
WB : anti-RAC
vimentin
WB : anti-vimentin

Supplementary Fig. 2: Validation of RAC-GTP antibody staining. (a) RAC-GTP and vimentin staining by immunofluorescence in biopsies from patients with triple negative (TNBC) and luminal B-like (LL) breast cancer, and in MDA-MB-468 cell line. Nucleus were detected by DAPI labelling. Cell profiles were performed to analyze each staining patterns. (scale bars: 10 µm biopsies ; 50 µm MDA-MB-468 cells). **(b)** RAC-GTP immunoprecipitation (IP) in MDA-MB-468 cells. RAC and vimentin proteins were detected in IP fraction and in the total cell lysate (TCL). IP without antibody was used as negative control (beads). **(c)** Western blot analyses of RAC-GTP, RAC and vimentin expression in MDA-MB-468 cells.

a

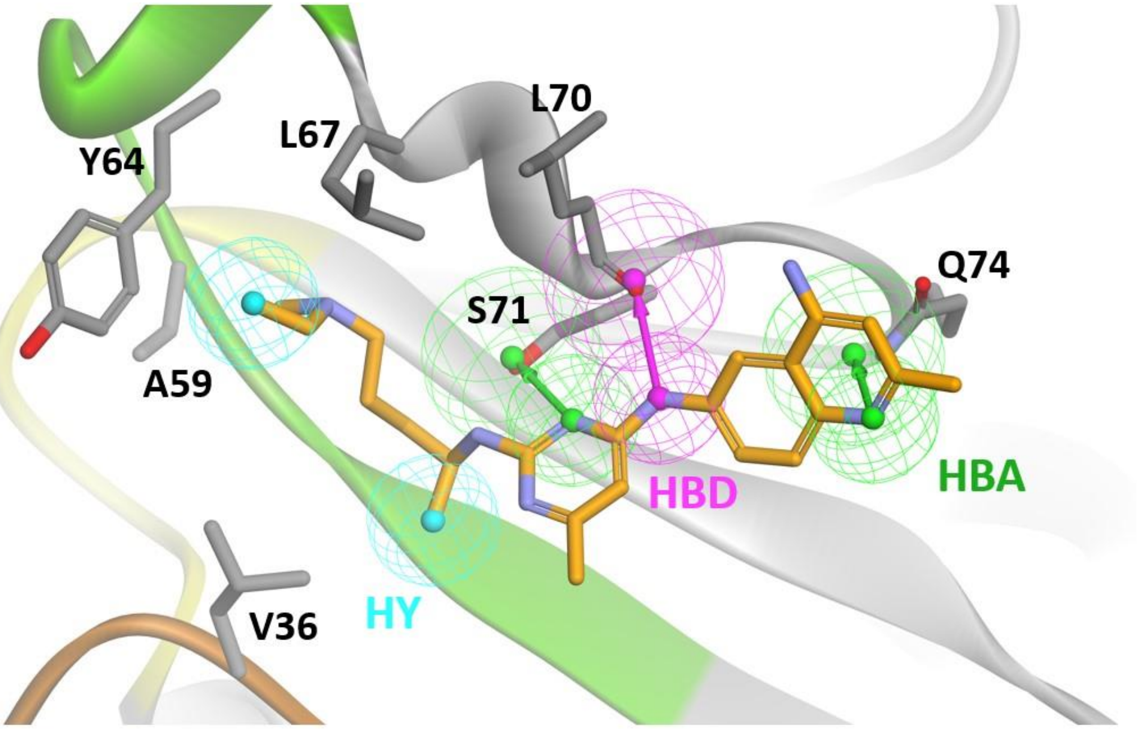

b

#Sample ID (10^{-5} M)	Adhesion (% inhib)	Migration (% inhib)	Proliferation (% ctrl)	Rac-GTP level (relative to EHT1864)
A4	58	34	100	0.38
D10	36	25	114	0.60
D9	42	10	110	0.77
B10	62	32	7	0.80
D6	37	35	94	0.82
A2	32	13	131	0.83
B3	51	22	51	0.95
EHT1864	32	35	101	1.00
G5	36	44	134	1.08
H7	40	14	144	1.41
A8	65	10	53	1.46
E6	48	27	101	2.44
E4	44	22	143	2.87
D11	37	40	88	3.79
NSC23766	34	20	131	N.D

Supplementary Fig. 3: Example of one pharmacophore model used for virtual screening. (a) The pharmacophore model shown was created based on RAC1 (grey) and NSC23766 (orange) interaction. The pharmacophore model is formed by two hydrogen bond acceptor (HBA) features, one oriented towards the hydroxyl group of Ser71 (S71) and the other towards the amine group of Gln74 (Q74), one hydrogen bond donor (HBD) feature pointed to the oxygen atom of Leu70 (L70) and two hydrophobic features (HY) located on certain carbon atoms of NSC23766 and facing residues Val36 (V36), Ala59 (A59), Tyr64 (Y64) and Leu67 (L67). The spheres correspond to location constraint. The pharmacophore model was completed by thirteen exclusion spheres centered on the main residues of RAC1 defining the binding site. The P loop, the switch 1 and the switch 2 are shown in yellow, orange and green respectively. **(b)** Impact of different screening molecules (10^{-5} M) on NIH/3T3 cell adhesion (xCELLingence Real-time cell analysis, Agilent), migration (CYTOO Mobility plates) and proliferation (xCELLingence Real-time cell analysis, Agilent). The level of RAC activation is determined by the detection of RAC-GTP (pull-down assay) in NIH/3T3 cell treated with EGF (10 ng/mL) and the different molecules and normalize to the level of RAC activity measured in the presence of the reference RAC inhibitor EHT1864.

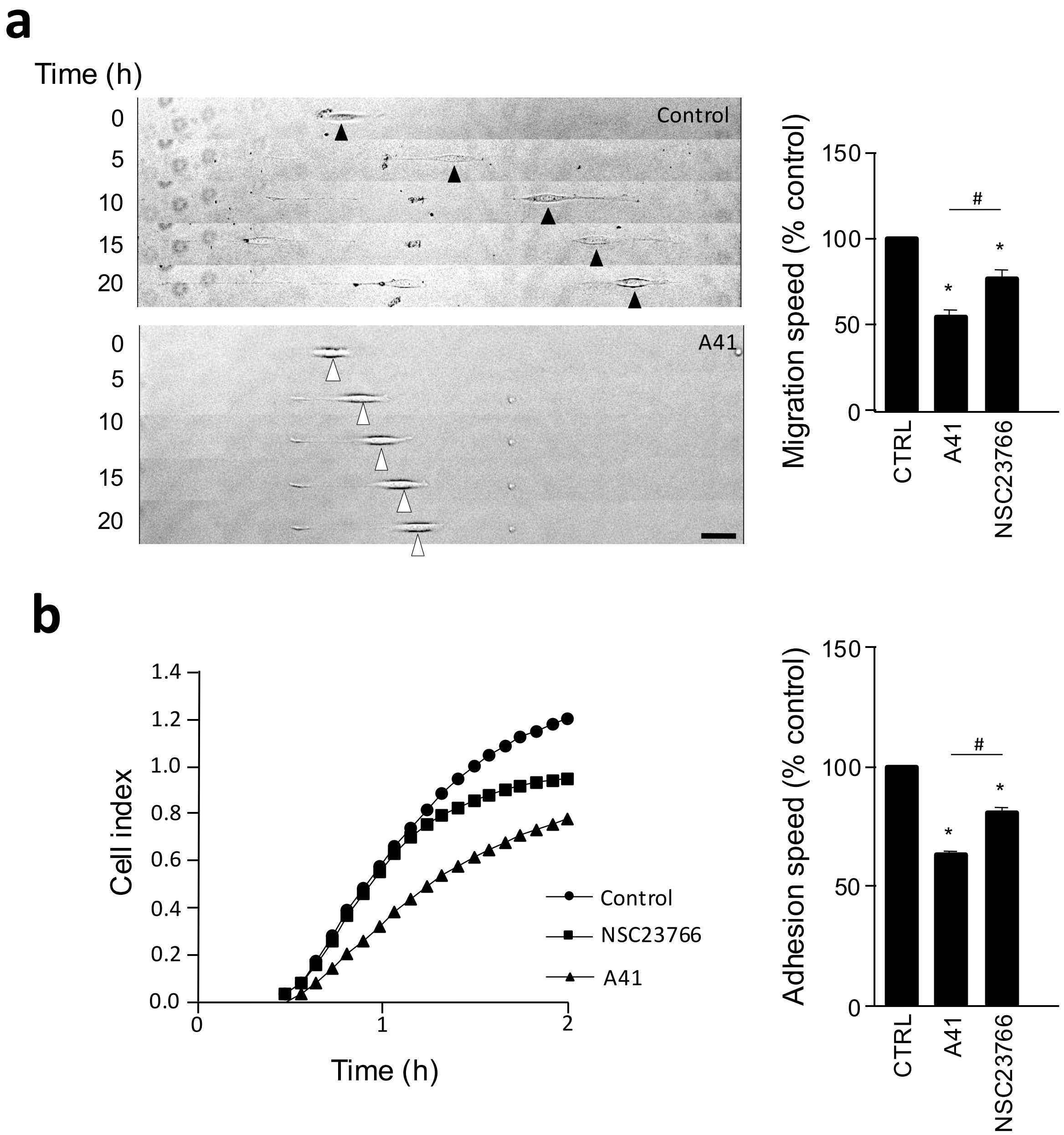
a
Time (h)
0
5
10
15
20
Control
0
5
10
15
20
A41
Migration speed (% control)
150
100
50
0
#
*
*
CTRL
A41
NSC23766
b
Cell index
1.4
1.2
1.0
0.8
0.6
0.4
0.2
0.0
0
1
2
Time (h)
Control
NSC23766
A41
Adhesion speed (% control)
150
100
50
0
#
*
*
CTRL
A41
NSC23766

–

Supplementary Fig. 4: A41 inhibits RAC-dependent cell functions. (a) Representative records of single NIH/3T3 cell migration on micropatterned fibronectin lines (CYTOO Mobility plates) in the absence (Control) and presence A41 at 10^{-5} M (left panel) and corresponding quantification of migration speed (right panel) (scale bar: 20µm). Graph also shown migration speed in the presence of the reference RAC inhibitor NSC23766 (10^{-5} M). (Results shown are representative of 3 independent experiments; *P<0.05 vs controls, #P<0.05 NSC23766). **(b)** Typical curves showing the cell index representing the adhesion of NIH/3T3 fibroblast (xCELLingence Real-time cell analysis, Agilent) under control condition (Control) and pre-treated with 10 µM A41 or NSC23766 (left panel) and corresponding quantification of cell adhesion speed (right panel). (Results shown are representative of 3 independent experiments; *P<0.05 vs control and $^{\#}$P<0.05 vs NSC23766).

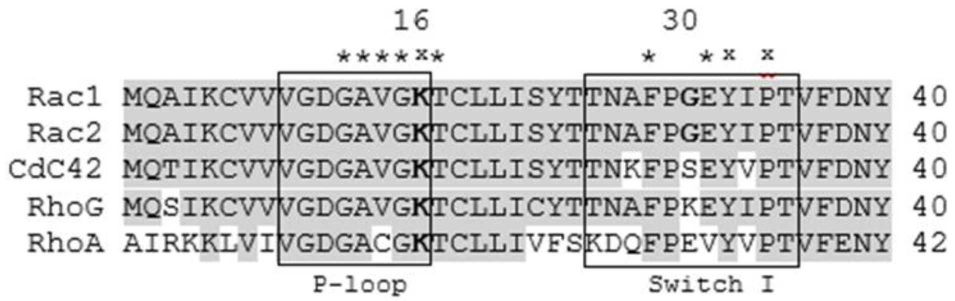

Supplementary Fig. 5: Alignment of indicated human RHO protein sequence focused on P-loop and Switch I domain.

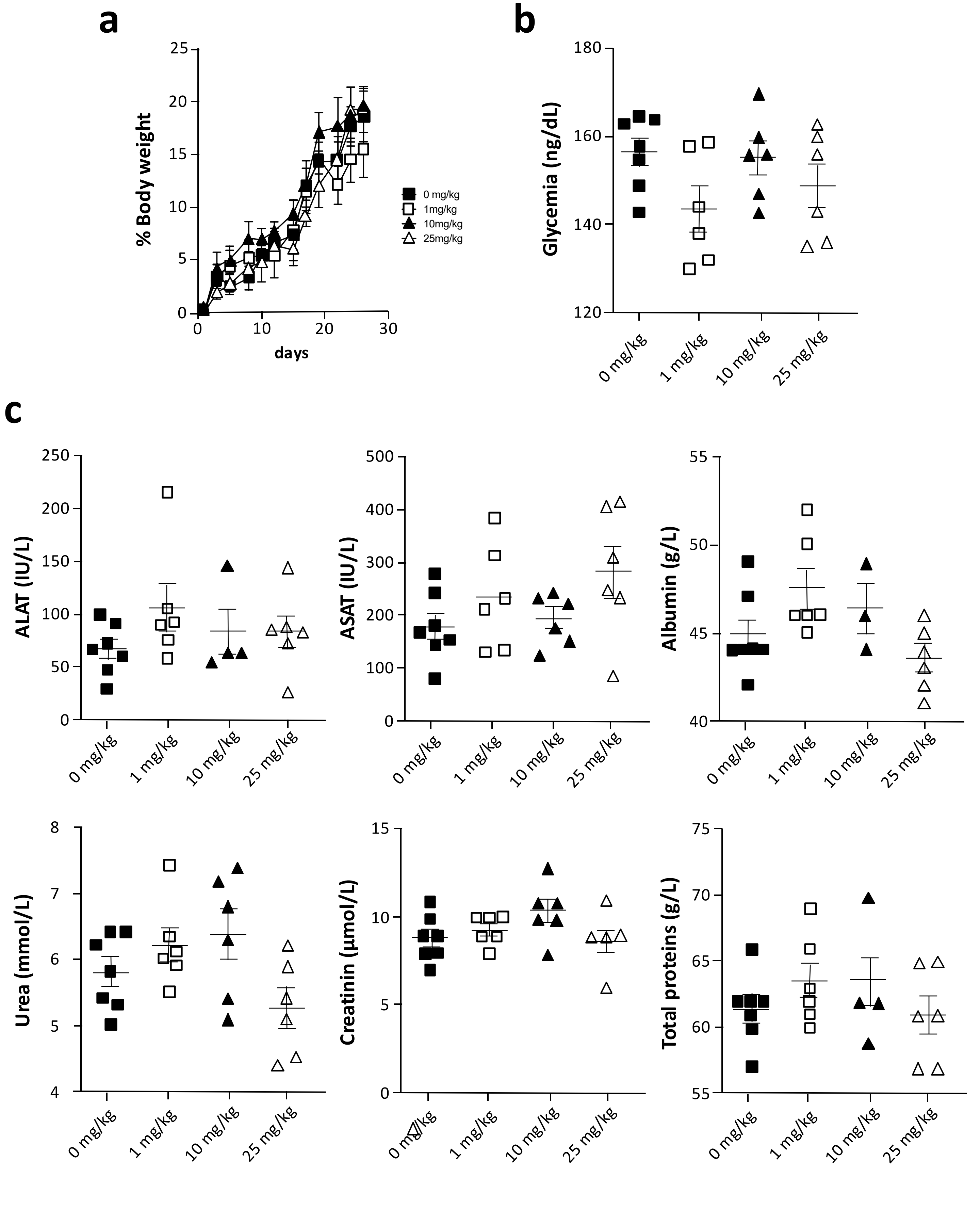
a
b
c
% Body weight
days
0 mg/kg
1mg/kg
10mg/kg
25mg/kg
Glycemia (ng/dL)
ALAT (IU/L)
ASAT (IU/L)
Albumin (g/L)
Urea (mmol/L)
Creatinin (µmol/L)
Total proteins (g/L)
0 mg/kg
1 mg/kg
10 mg/kg
25 mg/kg

Supplementary Fig. 6: ***In vivo*** **A41 toxicity. (a)** Effect of chronic administration of A41 at the indicated concentrations by daily intraperitoneal injections in mice on weight gain glycemia. **(b) and (c),** Glycemia **(b)** and plasmatic concentration of transaminases (ALAT and ASAT), albumin, urea, creatinine and total protein in mice after of 1 month of chronic administration of A41 at indicated concentrations **(c)**. N=6-7 mice.

a

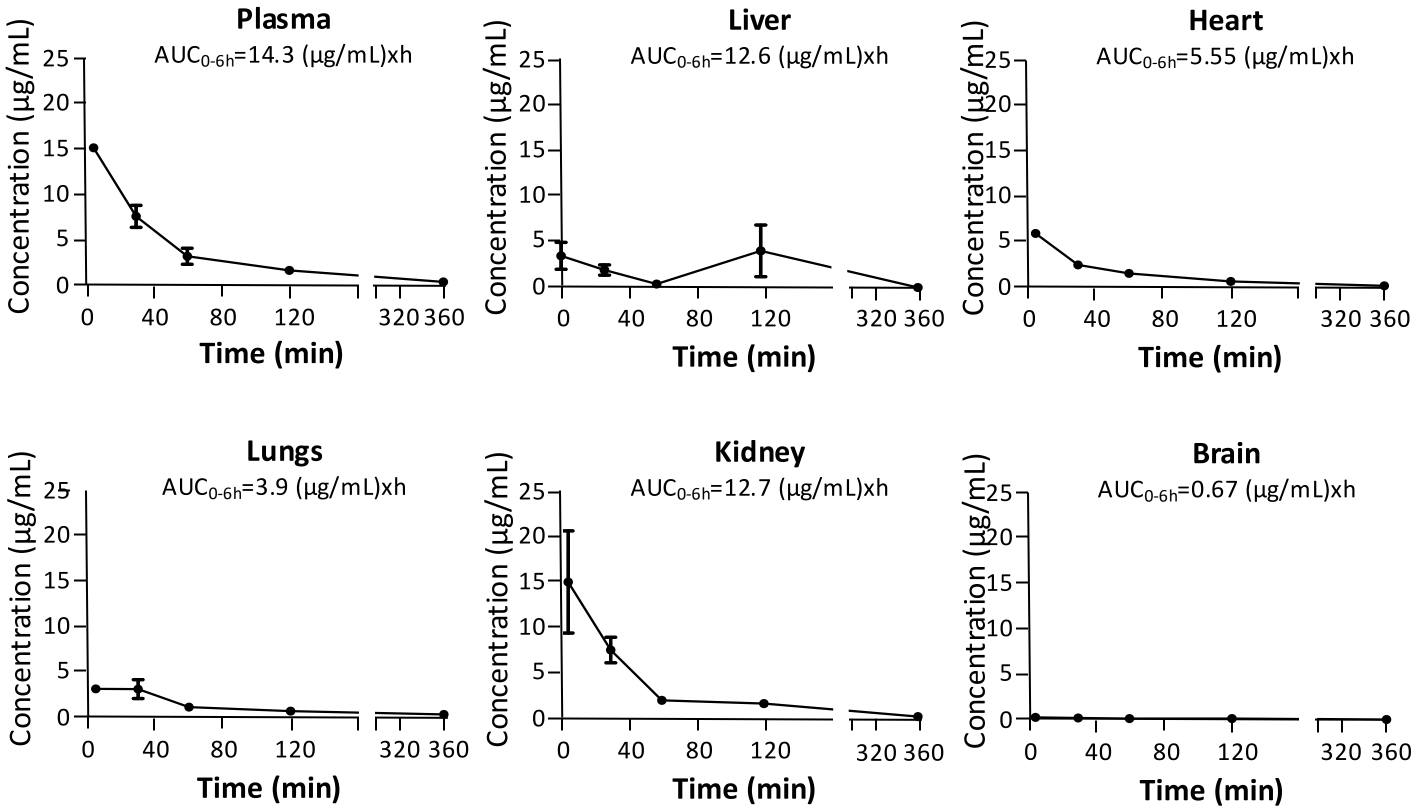

b

t ½ (min)	97.764
C_0 (µg/ml)	4.335
AUC_{0-6h} (µg*h/ml)	14.3
Vd (ml)	5.767
Cl (ml/h)	1.748
Cmax (µg/ml)	15.056 +/- 0.5
Tmax (min)	5

Supplementary Fig. 7: A41 pharmacokinetics. (a) Molecule distribution in plasma and indicated organs after intraperitoneal injection of A41 (25 mg/kg) in mice (N=3 mice/kinetic time point). **(b)** Pharmacokinetic parameters of A41 calculated in mice after a single intraperitoneal injection (25mg/kg). AUC = Area Under the Concentration-time curve.

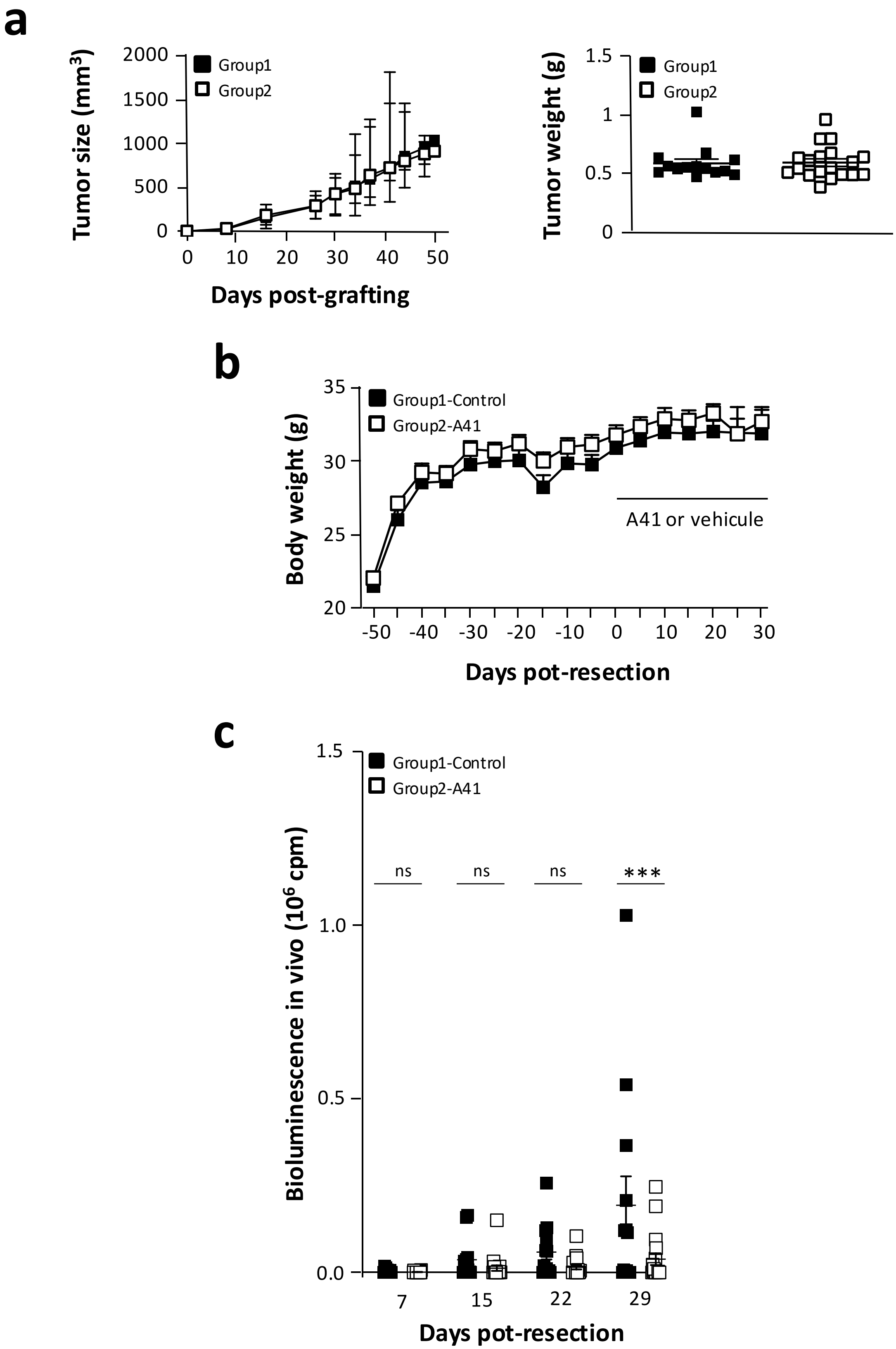
a
Tumor size (mm^3)
2000
1500
1000
500
0
0
10
20
30
40
50
Days post-grafting
Group1
Group2
Tumor weight (g)
1.5
1
0.5
0
b
Body weight (g)
35
30
25
20
Group1-Control
Group2-A41
A41 or vehicule
-50
-40
-30
-20
-10
0
10
20
30
Days pot-resection
c
Bioluminescence in vivo (10^6 cpm)
1.5
1.0
0.5
0.0
Group1-Control
Group2-A41
ns
ns
ns
***
7
15
22
29
Days pot-resection

Supplementary Fig. 8: Effect of A41 on the mice model of TNBC. (**a**) Time course of tumor growth estimated by measuring tumor size in the two experimental groups after transplantation of MDA-MB-468Luc cells (left) and weight of the tumor removed at day 50 in both group mice (right). **(b)** Time-course of the body weight of mice treated with vehicle (Group 1) or A41 (25 mg/kg/d) from the day before resection of the primary tumor resection. **(c)** *In vivo* measurement of bioluminescence in mice at indicated days after primary tumor resection.

	TNBC (N=18)	LL (N=20)
**Histologic.subtype**		
Poorly differenciated	16/18	16/20
Micropapillary	0/18	1/20
Colloïdal 20%	0/18	1/20
Non specified	2/18	2/20
**Status**		
Remission	10/18	10/20
Recurrence	8/18	10/20
**Age.Dg (years)**		
Median	63.000	60.500
Q1, Q3	52.0, 72.2	47.7, 65.5
**Laterality**		
Left	12/18	9/20
Right	6/18	11/20
**Neoadjuvant.Chemotherapy**		
NO	17/18	20/20
YES	1/18	0/20
**Breast.Surgery**		
Conservative	13/18	15/20
Mastectomy	5/18	5/20
**LymphNode.Surgery**		
GAS	13/18	4/20
CURAGE	5/18	16/20
**Stade.UICC**		
I	10/18	5/20
IIA	3/18	6/20
IIB	1/18	2/20
IIIA	2/18	5/20
IIIB	1/18	1/20
IIIC	1/18	1/20
**Histologic.Grade**		
-1	1/18	2/20
-2	5/18	5/20
-3	12/18	13/20
**CIS**		
NO	8/18	8/20
YES	10/18	12/20
**Embols**		
NO	13/18	6/20
YES	5/18	14/20
**Lymphocytic.infiltration**		
NO	14/18	20/20
YES	4/18	0/20
**RE.H**		
Negative	17/18	0/20
Positive	1/18	20/20
**RP.H**		
Negative	18/18	1/20
Positive	0/18	19/20

	TNBC (N=18)	LL (N=20)
**Radiotherapy.Breast.ChestWall**		
NO	2/18	0/20
YES	16/18	20/20
**Radiotherapy.LymphNodes.area**		
NO	15/18	9/20
YES	3/18	11/20
**Chemotherapy.Type**		
NO	3/18	3/20
ANTHRACYCLINE	2/18	6/20
TAXANE	1/18	0/20
ANTHRACYCLINE + TAXANE	11/18	11/20
ANTHRACYCLINE + TAXANE + BEVACIZUMAB	1/18	0/20
**Hormonotherapy.Adjuvant**		
NO	17/18	0/20
YES	1/18	20/20
**Tumoral emboli**		
NO	13/18	6/20
YES	5/18	14/20

Supp Table 1. Clinicopathologic data of TNBC and Luminal B-like (LL) patients included in the analyze of RAC1 activity.

			Druglikeness					
**Name**	**Structure**	**Molecular Formula**	**MW**	**logP**	**HBD**	**HBA**	**Docking score**	**IC_{50} (nM)**
A4	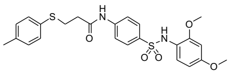	$C_{24}H_{26}N_2O_5S_2$	486.60	3.977	2	6	-10.32	34.2
**A41**	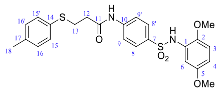	**$C_{24}H_{26}N_2O_5S_2$**	**486.60**	**3.977**	**2**	**6**	**-11.12**	**2.5**
A414	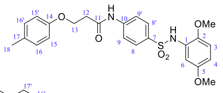	$C_{24}H_{26}N_2O_6S$	470.54	3.339	2	6	-11.62	0.56
A416	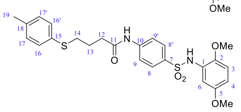	$C_{25}H_{28}N_2O_5S_2$	500.63	4.432	2	6	-9.92	2600
A415	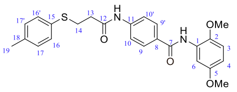	$C_{25}H_{26}N_2O_4S$	450.55	4.826	2	5	-7.53	-

Supp. Table 2. Chemical library derived from A4 compound. For each molecule, druglikeness has been assessed according to Lipinski's rules: molecular weight (MW)<500; high lipophilicity (LogP<5); less than 5 hydrogen bond donors (HBD); less than 10 hydrogen bond acceptors (HBA). The log P was calculated by using OSIRIS – Data Warrior software. The docking score (kcal/mol) for the nucleotide binding site corresponds to the value of the binding free energy after *in situ* ligand minimization divided by the number of heavy atoms of each ligand. IC_{50} was determined experimentally from the inhibition of ruffles formation.

Name	Structure	Molecular Formula	Druglikeness				Docking score	Inhbition (%)
			MW	logP	HBD	HBA		
**A41**	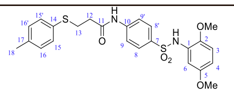	$C_{24}H_{26}N_2O_5S_2$	486.60	3.977	2	6	-11.12	46
**[N_3]-A41**	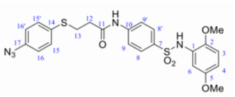	$C_{24}H_{24}N_2O_5S_2{}^2H_3$	513.59	3.905	2	7	-11.08	46

Supp. Table 3. Chemical property of [N3]- A41 compound. For each molecule, druglikeness has been assessed according to Lipinski's rules: molecular weight (MW)<500; high lipophilicity (LogP<5); less than 5 hydrogen bond donors (HBD); less than 10 hydrogen bond acceptors (HBA). The docking score (kcal/mol) for the nucleotide binding site corresponds to the value of the binding free energy after *in situ* ligand minimization divided by the number of heavy atoms of each ligand. Inhibition of RAC1 activity was determined experimentally from the inhibition of nucleotide exchange assay.

Type	Sequence<br>MS/MS fragments	Fragment ion (*m/z*)<br>Precursor: *m/z* 530.8	Fragment ion (*m/z*)<br>Precursor: *m/z* 773.4	Mass shift<br>A469: 485 Da
y_{10}^+	VVVGDGAVGK	900.8	-	-
y_9^+	VVGDGAVGK	801.7	1286.6	Yes
y_8^+	VGDGAVGK	702.6	1187.6	Yes
y_7^+	GDGAVGK	603.5	-	-
y_6^+	DGAVGK	546.5	1031.6	Yes
y_5^+	GAVGK	431.4	916.5	Yes
y_4^+	AVGK	374.5	-	-
y_3^+	VGK	303.3	788.5	Yes
**y_2^+**	**GK**	**204.2**	**689.5**	**Yes**
y_1^+	K	-	-	-
b_1^+	C	-	-	-
b_2^+	CV	260.2	260.2	No
b_3^+	CVV	-	-	-
b_4^+	CVVV	458.3	458.3	No
b_5^+	CVVVG	515.4	515.4	No
b_6^+	CVVVGD	-	-	-
b_7^+	CVVVGDG	-	-	-
b_8^+	CVVVGDGA	758	758	No
b_9^+	CVVVGDGAV	-	-	-
**b_{10}^+**	**CVVVGDGAVG**	**914**	**914**	**No**

Supp Table 4. Fragmentation patterns of unlabeled (precursor: *m/z* 530.8) and labeled (precursor: *m/z* 773.4) CVVVGDGAVGK peptide.

Tissue	Cell line	Mutation
Breast	MDA-MB-468	*P53*
	MDA-MB-231	*KRAS, BRAF*
	MDA-MB-435s	*BRAF*
Colon	LS147T	*KRAS, PI3K*
	HCT 116	*KRAS, PI3K*
	SW948	*PI3K*
	HT29	*BRAF*
	SW48	NA
Prostate	PC3	*PTEN*
Lung	NCI-H358	*KRAS*
	NCI-H460	*KRAS, PI3K*
	NCI-H1975	*PI3K*
Skin	A375	*BRAF*

Supp Table 5. Known mutations of cancer cell lines from various organs and tissues used in clonogenic assays.
