# Supplementary material for: A Rac-specific competitive inhibitor of guanine nucleotide binding reduces metastasis in triple-negative breast cancer
Source: Cell Rep Med. 2025 Jul 8;6(7):102233. doi: 10.1016/j.xcrm.2025.102233 (PMC12281424; doi:10.1016/j.xcrm.2025.102233)
Supplement: Data S1. Data S1–S8 [file mmc2.pdf]

Data S1. Safety screen assay A41

Assay Name	Spec.	% inh.
ATPase, Na^+/K^+, Heart, Pig	pig	-15
Cholinesterase, Acetyl, ACES	hum	64
Cyclooxygenase COX-1	hum	-11
Cyclooxygenase COX-2	hum	11
Monoamine Oxidase MAO-A	hum	10
Monoamine Oxidase MAO-B	hum	17
Peptidase, Angiotensin Converting Enzyme	rabbit	11
Peptidase, CTSG (Cathepsin G)	hum	-2
Phosphodiesterase PDE3	hum	9
Phosphodiesterase PDE4	hum	4
Protein Ser/Thr Kinase, PKC, Non- Selective	rat	48
Protein Tyrosine Kinase, Insulin Receptor	hum	-25
Protein Tyrosine Kinase, LCK	hum	14
Adenosine A1	hum	2
Adenosine A2A	hum	16
Adrenergic α1A	rat	34
Adrenergic α1B	rat	41
Adrenergic α1D	hum	21
Adrenergic α2A	hum	29
Adrenergic α2B	hum	9
Adrenergic β1	hum	2
Adrenergic β2	hum	5
Androgen (Testosterone)	hum	10
Angiotensin AT1	hum	17
Bradykinin B2	hum	1
Calcium Channel L-Type, Benzothiazepine	rat	46
Calcium Channel L-Type, Dihydropyridine	rat	85
Calcium Channel L-Type, Phenylalkylamine	rat	52
Calcium Channel N-Type	rat	3
Cannabinoid CB1	hum	81
Cannabinoid CB2	hum	36
Chemokine CCR1	hum	2
Chemokine CXCR2 (IL-8RB)	hum	1
Cholecystokinin CCK1 (CCKA)	hum	31
Cholecystokinin CCK2 (CCKB)	hum	0
Dopamine D1	hum	17
Dopamine D2L	hum	9
Dopamine D2S	hum	35
Endothelin ETA	hum	-8
Estrogen ERα	hum	10
GABAA, Chloride Channel, TBOB	rat	45
GABAA, Flunitrazepam, Central	rat	15
GABAA, Ro-15-1788, Hippocampus	rat	-12
GABAB1A	hum	-6
Glucocorticoid	hum	17

Assay Name	Spec.	% inh.
Glutamate, NMDA, Glycine	rat	7
Glutamate, NMDA, Phencyclidine	rat	-6
Glutamate, NMDA, Polyamine	rat	19
Glycine, Strychnine-Sensitive	rat	-1
Histamine H1	hum	41
Histamine H2	hum	-14
Leukotriene, Cysteinyl CysLT1	hum	14
Melanocortin MC1	hum	14
Melanocortin MC4	hum	8
Muscarinic M1	hum	11
Muscarinic M2	hum	12
Muscarinic M3	hum	4
Muscarinic M4	hum	-1
Neuropeptide Y Y1	hum	-2
Nicotinic Acetylcholine	hum	-3
Nicotinic Acetylcholine α1, Bungarotoxin	hum	0
Opiate δ1 (OP1, DOP)	hum	6
Opiate κ(OP2, KOP)	hum	10
Opiate μ(OP3, MOP)	hum	14
Platelet Activating Factor (PAF)	hum	78
Potassium Channel [KATP]	ham	11
Potassium Channel hERG	hum	22
PPARγ	hum	-7
Progesterone PR-B	hum	-16
Serotonin (5-Hydroxytryptamine) 5-HT1A	hum	6
Serotonin (5-Hydroxytryptamine) 5-HT1B	hum	15
Serotonin (5-Hydroxytryptamine) 5-HT2A	hum	84
Serotonin (5-Hydroxytryptamine) 5-HT2B	hum	90
Serotonin (5-Hydroxytryptamine) 5-HT2C	hum	56
Serotonin (5-Hydroxytryptamine) 5-HT3	hum	-2
Sodium Channel, Site 2	rat	57
Tachykinin NK1	hum	41
Transporter, Adenosine	gp	37
Transporter, Dopamine (DAT)	hum	30
Transporter, GABA	rat	-4
Transporter, Norepinephrine (NET)	hum	36
Transporter, Serotonin (5- Hydroxytryptamine) (SERT)	hum	-3

Data S2. Bacterial cytotoxicity of A41.

Test Concentration	%Effect			Cytotoxicity (% of control)
	1st	2nd	3rd	Mean %Effect
**Bacterial cytotoxicity (TA98 - S9)**				
6.0E-07 M	92.4	82.4	82.4	**85.7**
1.2E-06 M	89.7	74.1	55.8	**73.2**
2.5E-06 M	80.5	83.3	82.4	**82.0**
5.0E-06 M	88.8	84.2	81.4	**84.8**
1.0E-05 M	82.4	76.9	77.8	**79.0**
2.5E-05 M	95.2	92.4	83.3	**90.3**
5.0E-05 M	88.8	83.3	75.9	**82.7**
1.0E-04 M	154.6	177.5	143.7	**158.6**
**Bacterial cytotoxicity (TA100 - S9)**				
6.0E-07 M	104.7	104.7	98.2	**102.5**
1.2E-06 M	103.4	99.5	92.9	**98.6**
2.5E-06 M	106.0	103.4	92.9	**100.8**
5.0E-06 M	116.5	116.5	91.6	**108.2**
1.0E-05 M	116.5	108.6	92.9	**106.0**
2.5E-05 M	113.9	111.3	100.8	**108.6**
5.0E-05 M	112.6	53.1	102.1	**107.3**
1.0E-04 M	222.5	205.5	199.0	**209.0**
**Bacterial cytotoxicity (TA1535 -S9)**				
6.0E-07 M	89.4	96.4	94.0	**93.3**
1.2E-06 M	91.7	97.5	88.2	**92.5**
2.5E-06 M	92.9	91.7	97.5	**94.0**
5.0E-06 M	96.4	95.2	99.8	**97.1**
1.0E-05 M	96.4	98.7	108.0	**101.0**
2.5E-05 M	116.1	99.8	98.7	**104.9**
5.0E-05 M	175.3	168.3	163.7	**169.1**
1.0E-04 M	219.4	233.3	215.9	**222.9**
**Bacterial cytotoxicity (TA1537 - S9)**				
6.0E-07 M	107.2	107.2	115.3	**109.9**
1.2E-06 M	109.9	108.6	101.9	**106.8**
2.5E-06 M	111.2	104.5	99.2	**105.0**
5.0E-06 M	116.6	111.2	100.5	**109.4**
1.0E-05 M	109.9	108.6	111.2	**109.9**
2.5E-05 M	128.7	115.3	112.6	**118.8**
5.0E-05 M	162.2	150.1	148.8	**153.7**
1.0E-04 M	222.5	214.4	229.2	**222.0**

Data S3. Ames-fluctuation assay-A41.

Test Concentration	Count (# of wells)	Positive Significance (- to +++)	Fisher Exact Test (p-value)
**Ames fluctuation test**			
5.0E-06 M	0	-	1.0000
1.0E-05 M	2	-	0.2474
5.0E-05 M	0	-	1.0000
1.0E-04 M	1	-	0.5000
**Ames fluctuation test**			
5.0E-06 M	3	-	0.3085
1.0E-05 M	1	-	1.0000
5.0E-05 M	0	-	0.5000
1.0E-04 M	0	-	0.5000
**Ames fluctuation test (TA100 + S9**			
5.0E-06 M	8	-	0.0993
1.0E-05 M	5	-	0.3572
5.0E-05 M	5	-	0.3572
1.0E-04 M	3	-	1.0000
**Ames fluctuation test (TA100 + S9)**			
5.0E-06 M	6	-	0.1424
1.0E-05 M	10	-	0.5000
5.0E-05 M	9	-	0.4011
1.0E-04 M	7	-	0.2167
**Ames fluctuation test (TA1535 - S9)**			
5.0E-06 M	0	-	1.0000
1.0E-05 M	2	-	0.2474
5.0E-05 M	0	-	1.0000
1.0E-04 M	0	-	1.0000
**Ames fluctuation test (TA1535 + S9)**			
5.0E-06 M	1	-	0.5000
1.0E-05 M	0	-	0.2474
5.0E-05 M	0	-	0.2474
1.0E-04 M	1	-	0.5000
5.0E-06 M	1	-	0.5000
1.0E-05 M	0	-	1.0000
5.0E-05 M	0	-	1.0000
1.0E-04 M	1	-	0.5000
**Ames fluctuation test (TA1537 + S9)**			
5.0E-06 M	4	-	0.5000
1.0E-05 M	5	-	0.3572
5.0E-05 M	2	-	0.5000
1.0E-04 M	1	-	0.3085

Data S4. Micronucleus assay-A41.

Test Concentr.	Scored Cells	% Cytotox CBPI Index	% Cytotox Cell Numbers	% Micronucleated Cells	t-Test p-value	Test Result
**Micronucleus (CHO + S9, HCA)**						
6.3E-05 M	2143	-0.4	54.3	1.51	0.1759	-
1.3E-04 M	2168	3.0	63.7	1.66	0.0564	-
2.5E-04 M	2114	3.3	71.5	1.12	0.4390	-
5.0E-04 M	2130	46.8	72.1	2.16	0.0111	+/-
1.0E-03 M	486	82.3	77.9	2.68	0.0038	+/-
**Micronucleus (CHO - S9, HCA)**						
7.8E-06 M	2034	-21.4	14.3	1.13	0.0011	-
1.6E-05 M	2287	-23.9	26.4	2.04	0.1443	-
3.1E-05 M	2155	8.1	42.0	3.12	0.3177	-
6.3E-05 M	1868	65.8	52.5	6.96	0.0003	+/-
1.3E-04 M	1018	72.8	64.6	13.39	0.0377	+
2.5E-04 M	751	73.8	73.6	14.40	0.0000	+

Data S5. Kinase Profiler-A41.

Kinase	Activity(%)
AAK1(h)	105
Abl(h)	90
Abl(m)	125
Abl (H396P) (h)	92
Abl (M351T)(h)	111
Abl (Q252H) (h)	105
Abl(T315I)(h)	117
Abl(Y253F)(h)	102
ACK1(h)	103
ACTR2(h)	108
ALK(h)	82
ALK1(h)	102
ALK2(h)	100
ALK4(h)	101
ALK6(h)	104
Arg(h)	122
AMPKα1(h)	102
AMPKα2(h)	111
A-Raf(h)	85
Arg(m)	109
ARK5(h)	106
ASK1(h)	94
Aurora-A(h)	124
Aurora-B(h)	86
Aurora-C(h)	100
Axl(h)	101
BIKe(h)	112
Blk(h)	99
Blk(m)	87
BMPR2(h)	100
Bmx(h)	124
BRK(h)	107
BrSK1(h)	86
BrSK2(h)	118
BTK(h)	117
BTK(R28H)(h)	102
B-Raf(h)	90
B-Raf(V599E)(h)	87
CaMKI(h)	114
CaMKIß(h)	114
CaMKIγ(h)	121

Kinase	Activity(%)
CaMKIIα(h)	99
CaMKIIβ(h)	112
CaMKIIγ(h)	92
CaMKIδ(h)	94
CaMKIIδ(h)	92
CaMKIV(h)	114
CaMKK1(h)	95
CaMKK2(h)	110
Cdc7/cyclinB1(h)	92
CDK1/cyclinB(h)	96
CDK2/cyclinA(h)	97
CDK2/cyclinE(h)	100
CDK3/cyclinE(h)	77
CDK4/cyclinD3(h)	96
CDK5/p25(h)	110
CDK5/p35(h)	99
CDK6/cyclinD3(h)	111
CDK7/cyclinH/MAT1(h)	106
CDK9/cyclin T1(h)	92
CDK12/cyclinK(h)	100
CDK13/cyclinK(h)	97
CDK14/cyclinY(h)	89
CDK16/cyclinY(h)	107
CDK17/cyclinY(h)	96
CDK18/cyclinY(h)	101
CDKL1(h)	105
CDKL2(h)	103
CDKL3(h)	103
CDKL4(h)	91
ChaK1(h)	104
CHK1(h)	120
CHK2(h)	111
CHK2(I157T)(h)	112
CHK2(R145W)(h)	92
CK1α(h)	102
CK1ε(h)	108
CK1γ1(h)	106
CK1γ2(h)	112
CK1γ3(h)	101
CK1δ(h)	96
CK1(y)	98

Kinase	Activity(%)
CK2(h)	110
CK2α1(h)	80
CK2α2(h)	109
CLIK1(h)	112
CLK1(h)	81
CLK2(h)	97
CLK3(h)	110
CLK4(h)	102
cKit(h)	101
cKit(D816V)(h)	88
cKit(D816H)(h)	109
cKit(V560G)(h)	115
cKit(V654A)(h)	99
CRIK(h)	106
CSK(h)	119
c-RAF(h)	93
cSRC(h)	111
DAPK1(h)	117
DAPK2(h)	99
DCAMKL1(h)	99
DCAMKL2(h)	103
DCAMKL3(h)	104
DDR1(h)	116
DDR2(h)	114
DMPK(h)	98
DRAK1(h)	101
DRAK2(h)	90
DYRK1A(h)	93
DYRK1B(h)	96
DYRK2(h)	104
DYRK3(h)	112
eEF-2K(h)	97
EGFR(h)	115
EGFR(L858R)(h)	104
EGFR(L861Q)(h)	107
EGFR(T790M)(h)	113
EGFR(T790M,L858R)(h)	104
EphA1(h)	87
EphA2(h)	98
EphA3(h)	139
EphA4(h)	111

Kinase	Activity(%)	Kinase	Activity(%)	Kinase	Activity(%)
EphA5(h)	110	HIPK3(h)	95	MAP4K4(h)	107
EphA7(h)	108	HIPK4(h)	105	MAP4K5(h)	102
EphA8(h)	112	HPK1(h)	98	MAPKAP-K2(h)	111
EphB2(h)	97	HRI(h)	98	MAPKAP-K3(h)	112
EphB1(h)	111	ICK(h)	77	MEK1(h)	97
EphB3(h)	113	IGF-1R(h)	100	MEK2(h)	109
EphB4(h)	112	IGF-1R(h), activated	108	MARK1(h)	110
ErbB2(h)	116	IKKα(h)	107	MARK3(h)	98
ErbB4(h)	108	IKKβ(h)	110	MARK4(h)	105
FAK(h)	111	IKKε(h)	106	MEKK2(h)	102
Fer(h)	114	IR(h)	137	MEKK3(h)	109
Fes(h)	116	IR(h), activated	104	MELK(h)	107
FGFR1(h)	83	IRE1(h)	101	Mer(h)	90
FGFR1(V561M)(h)	129	IRR(h)	118	Met(h)	88
FGFR2(h)	89	IRAK1(h)	104	Met(D1246H)(h)	115
FGFR2(N549H)(h)	105	IRAK4(h)	99	Met(D1246N)(h)	119
FGFR3(h)	89	Itk(h)	78	Met(M1268T)(h)	108
FGFR4(h)	116	JAK1(h)	99	Met(Y1248C)(h)	118
Fgr(h)	114	JAK2(h)	114	Met(Y1248D)(h)	118
Flt1(h)	103	JAK3(h)	106	Met(Y1248H)(h)	120
Flt3(D835Y)(h)	101	JNK1α1(h)	94	MINK(h)	114
Flt3(h)	98	JNK2α2(h)	101	MKK3(h)	117
Flt4(h)	92	JNK3(h)	123	MKK4(m)	109
Fms(h)	112	KDR(h)	113	MKK6(h)	90
Fms(Y969C)(h)	107	LATS1(h)	98	MLCK(h)	103
Fyn(h)	102	LATS2(h)	110	MLK1(h)	102
GCK(h)	114	Lck(h)	74	MLK2(h)	109
GCN2(h)	101	Lck(h) activated	90	MLK3(h)	115
GRK1(h)	103	LIMK1(h)	113	MLK4(h)	104
GRK2(h)	96	LIMK2(h)	115	Mnk2(h)	107
GRK3(h)	106	LKB1(h)	107	MOK(h)	90
GRK5(h)	101	LOK(h)	112	MRCKα(h)	109
GRK6(h)	100	Lyn(h)	109	MRCKβ(h)	102
GRK7(h)	100	Lyn(m)	110	MRCKγ(h)	97
GSK3α(h)	90	LRRK2(h)	111	MSK1(h)	117
GSK3β(h)	90	LTK(h)	113	MSK2(h)	105
Haspin(h)	116	MAK(h)	117	MSSK1(h)	125
Hck(h)	111	MAPK1(h)	103	MST1(h)	96
Hck(h) activated	102	MAPK2(h)	103	MST2(h)	88
HIPK1(h)	110	MAPK2(m)	108	MST3(h)	95
HIPK2(h)	81	MAP4K3(h)	98	MST4(h)	101

Kinase	Activity(%)
mTOR(h)	101
mTOR/FKBP12(h)	104
MuSK(h)	112
MYLK2(h)	103
MYO3B(h)	106
NDR1(h)	114
NDR2(h)	112
NEK1(h)	104
NEK2(h)	118
NEK3(h)	107
NEK4(h)	108
NEK6(h)	109
NEK7(h)	97
NEK9(h)	107
NIM1(h)	105
NEK11(h)	99
NLK(h)	99
NUAK2(h)	110
OSR1(h)	103
p70S6K(h)	103
PAK1(h)	115
PAK2(h)	110
PAK4(h)	106
PAK3(h)	107
PAK5(h)	105
PAK6(h)	99
PAR-1Bα(h)	111
PASK(h)	97
PEK(h)	102
PDGFRα(h)	92
PDGFRα(D842V)(h)	112
PDGFRα(V561D)(h)	119
PDGFRβ(h)	82
PDHK2(h)	96
PDHK4(h)	93
PDK1(h)	107
PhKγ1(h)	102
PhKγ2(h)	98
Pim-1(h)	95
Pim-2(h)	110
Pim-3(h)	101

Kinase	Activity(%)
PKA(h)	84
PKAcβ(h)	116
PKBα(h)	108
PKBβ(h)	116
PKBγ(h)	98
PKCα(h)	105
PKCβI(h)	98
PKCβII(h)	97
PKCγ(h)	112
PKCδ(h)	110
PKCε(h)	102
PKCη(h)	118
PKCι(h)	93
PKCμ(h)	104
PKCθ(h)	114
PKCζ(h)	97
PKD2(h)	100
PKD3(h)	104
PKG1α(h)	113
PKG1β(h)	105
PKR(h)	101
Plk1(h)	106
Plk3(h)	115
Plk4(h)	104
PRAK(h)	88
PRKG2(h)	123
PRK1(h)	93
PRK2(h)	110
PrKX(h)	98
PRP4(h)	110
PTK5(h)	113
Pyk2(h)	92
Ret(h)	104
Ret (V804L)(h)	93
Ret(V804M)(h)	106
RIPK1(h)	98
RIPK2(h)	94
ROCK-I(h)	101
ROCK-II(h)	93
ROCK-II(r)	110
Ron(h)	110

Kinase	Activity(%)
Ros(h)	139
Rse(h)	102
Rsk1(h)	122
Rsk1(r)	128
Rsk2(h)	86
Rsk3(h)	93
Rsk4(h)	128
SAPK2a(h)	100
SAPK2a(T106M)(h)	98
SAPK2b(h)	107
SAPK3(h)	105
SAPK4(h)	104
SBK1(h)	101
SGK(h)	116
SGK2(h)	117
SGK3(h)	106
SIK(h)	144
SIK2(h)	90
SIK3(h)	106
SLK(h)	127
Snk(h)	112
SNRK(h)	123
Src(1-530)(h)	105
Src(T341M)(h)	89
SRMS(h)	102
SRPK1(h)	114
SRPK2(h)	96
STK16(h)	115
STK25(h)	98
STK32A(h)	100
STK32B(h)	105
STK32C(h)	105
STK33(h)	98
STK39(h)	91
Syk(h)	91
TAF1L(h)	110
TAK1(h)	107
TAO1(h)	118
TAO2(h)	108
TAO3(h)	97
TBK1(h)	102

Kinase	Activity(%)
Tec(h) activated	112
TGFBR1(h)	103
TGFBR2(h)	99
Tie2 (h)	110
Tie2(R849W)(h)	101
Tie2(Y897S)(h)	109
TLK1(h)	102
TLK2(h)	101
TNIK(h)	94
TRB2(h)	104
TrkA(h)	99
TrkB(h)	108
TrkC(h)	104
TSSK1(h)	103
TSSK2(h)	111
TSSK3(h)	104
TSSK4(h)	103
TTBK1(h)	103
TTBK2(h)	106
TTK(h)	103
Txk(h)	118
TYK2(h)	108
ULK1(h)	106
ULK2(h)	99
ULK3(h)	97
VRK1(h)	103
VRK2(h)	110
Wee1(h)	112
Wee1B(h)	89
WNK1(h)	96
WNK2(h)	90
WNK3(h)	101
WNK4(h)	107
Yes(h)	110
ZAK(h)	109
ZAP-70(h)	108
ZIPK(h)	103
ATM(h)	114
ATR/ATRIP(h)	93
DNA-PK(h)	122
PI3K (p110β/p85α)(h)	101

Kinase	Activity(%)
PI3K (p120γ)(h)	104
PI3K (p110δ/p85α)(h)	100
PI3K (p110α/p85α)(m)	102
PI3K (p110α/p65α)(m)	100
PI3K (p110α(E545K)/p85α)(m)	100
PI3K (p110α(H1047R)/p85α)(m)	100
PI3K (p110β/p85β)(m)	100
PI3K (p110β/p85α)(m)	101
PI3K (p110δ/p85α)(m)	100
PI3K (p110α(E542K)/p85α)(m)	99
PI3K (p110α/p85α)(h)	100
PI3K (p110α(E542K)/p85α)(h)	105
PI3K (p110α(H1047R)/p85α)(h)	97
PI3K (p110α(E545K)/p85α)(h)	105
PI3K e (p110α/p65α)(h)	102
PI3KC2α(h)	95
PI3KC2γ(h)	114
PIP4K2α(h)	95
PIP5K1α(h)	100
PIP5K1γ(h)	104

Data S6. Formule white blood cell (% of leukocytes) in control and A41-treated mice (25 mg/kg/d).

	**Control** (n=5)	**A41** (n=5)	**p value**
**B cells**			
Mean (SD)	14.11 (2.69)	11.62 (1.58)	0.14
**T cells**			
Mean (SD)	44.72 (8.28)	52.20 (2.11)	0.22
**NK cells**			
Mean (SD)	0.44 (0.14)	0.40 (0.22)	0.81
**Macrophages**			
Mean (SD)	2.74 (1.85)	2.46 (2.34)	0.77
**Monocytes**			
Mean (SD)	0.72 (0.15)	0.86 (0.57)	1
**Neutrophiles**			
Mean (SD)	4.88 (5.16)	4.19 (6.24)	1

Data S7. NMR spectra of the intermediates 1, 2, 3 and final compounds A4.1, A4.14, A4.16, A4.20

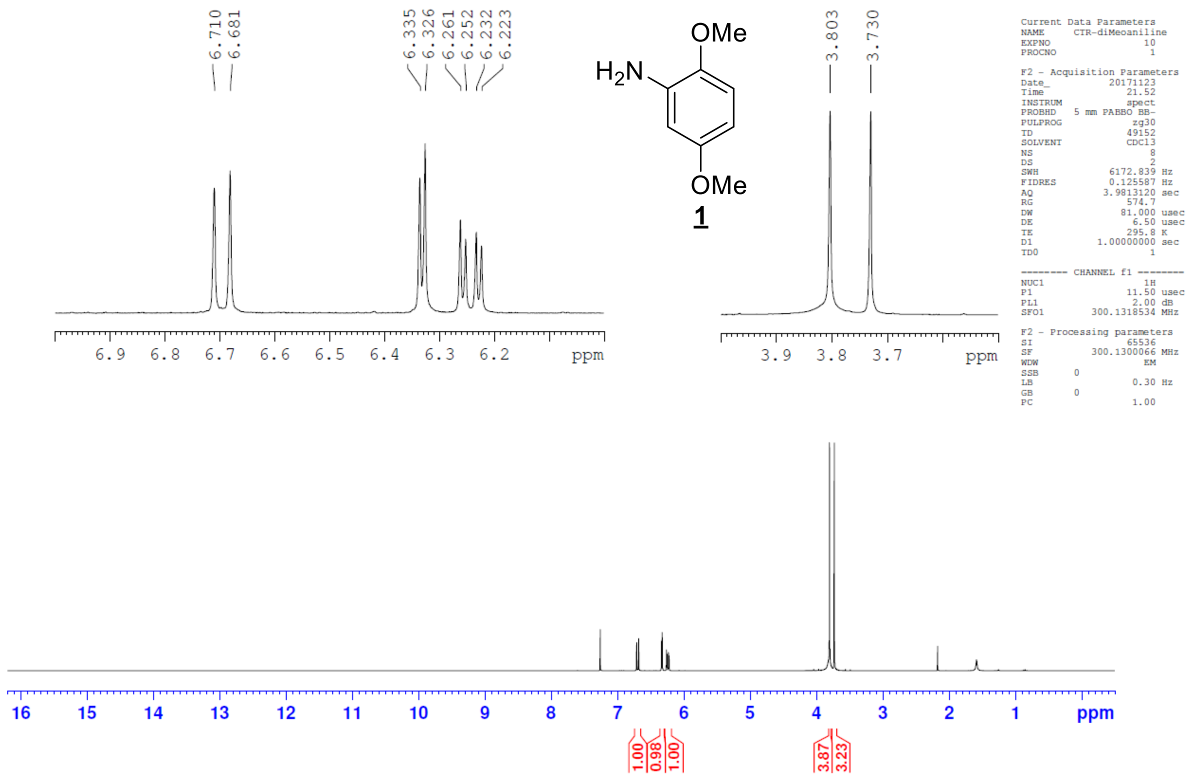

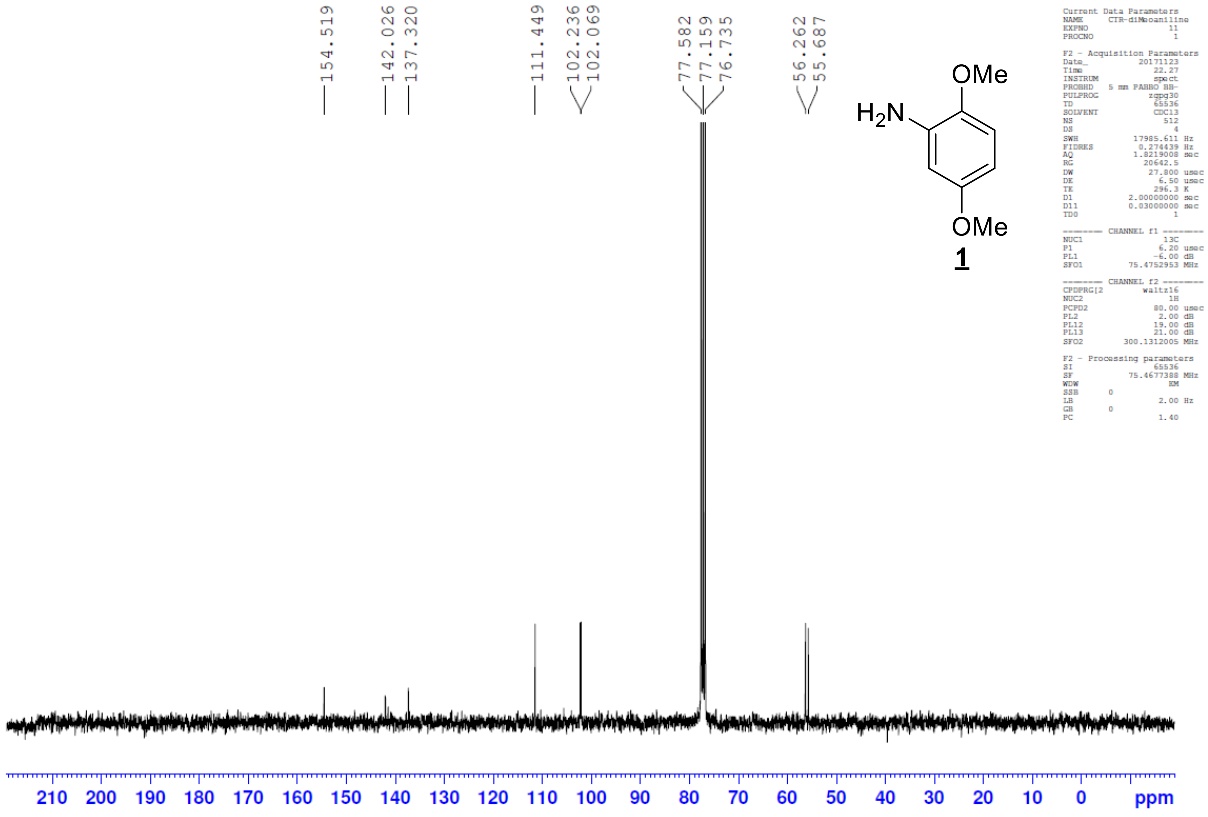

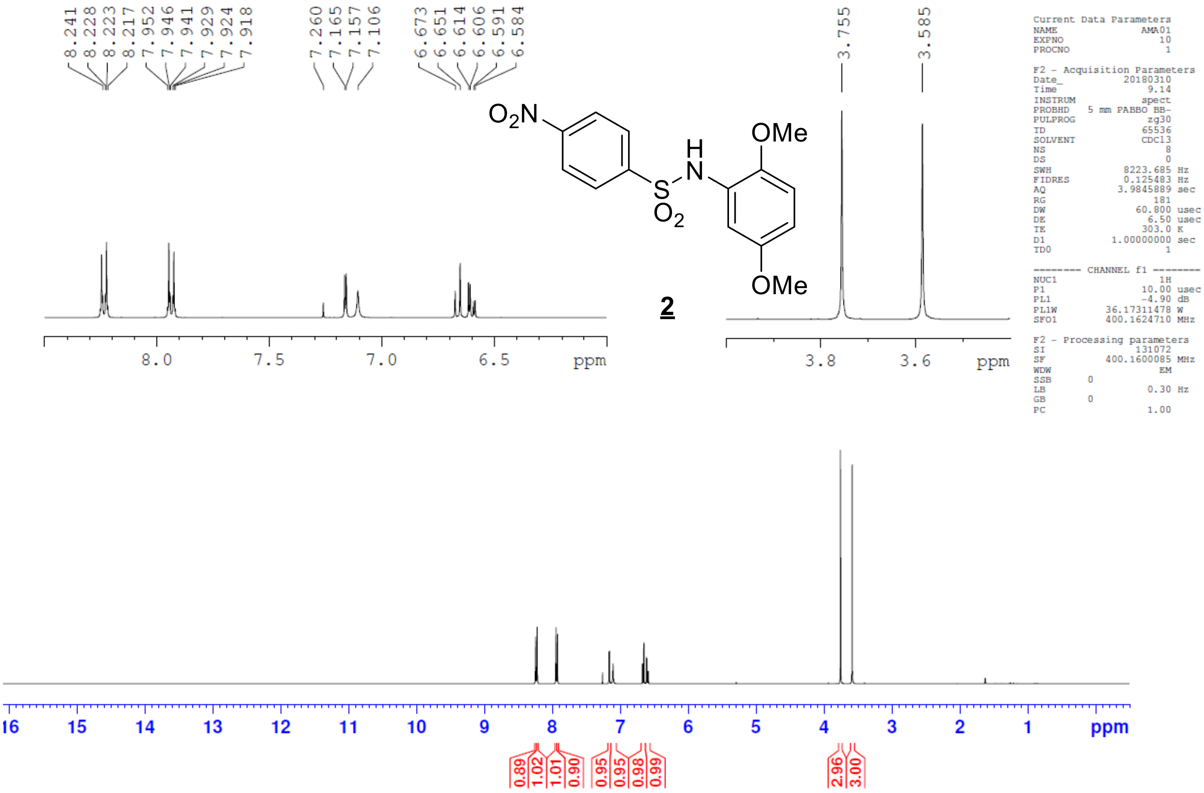

8.241
8.228
8.223
8.217
7.952
7.946
7.941
7.929
7.924
7.918
7.260
7.165
7.157
7.106
6.673
6.651
6.614
6.606
6.591
6.584
3.755
3.585
O2N
H
N
S
O2
OMe
OMe
2
8.0
7.5
7.0
6.5
ppm
3.8
3.6
ppm
Current Data Parameters
NAME AMA01
EXPNO 10
PROCNO 1
F2 - Acquisition Parameters
Date_ 20180310
Time 9.14
INSTRUM spect
PROBHD 5 mm PABBO BB-
PULPROG zg30
TD 65536
SOLVENT CDCl3
NS 8
DS 0
SWH 8223.685 Hz
FIDRES 0.125483 Hz
AQ 3.9845889 sec
RG 181
DW 60.800 usec
DE 6.50 usec
TE 303.0 K
D1 1.00000000 sec
TD0 1
======== CHANNEL f1 ========
NUC1 1H
P1 10.00 usec
PL1 -4.90 dB
PL1W 36.17311478 W
SFO1 400.1624710 MHz
F2 - Processing parameters
SI 131072
SF 400.1600085 MHz
WDW EM
SSB 0
LB 0.30 Hz
GB 0
PC 1.00
16
15
14
13
12
11
10
9
8
7
6
5
4
3
2
1
ppm
0.89
1.02
1.01
0.90
0.95
0.95
0.98
0.99
2.96
3.00

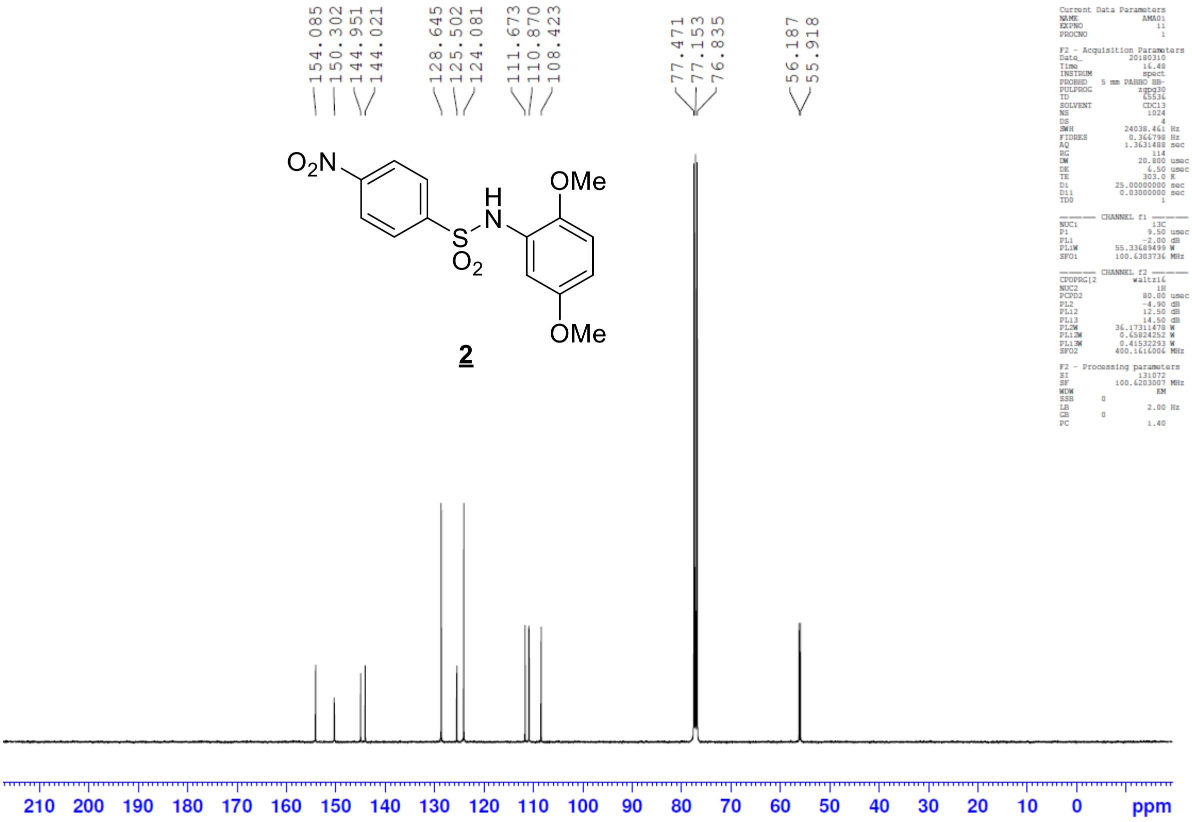

154.085
150.302
144.951
144.021
128.645
125.502
124.081
111.673
110.870
108.423
77.471
77.153
76.835
56.187
55.918
O2N
H
N
S
O2
OMe
OMe
2
210
200
190
180
170
160
150
140
130
120
110
100
90
80
70
60
50
40
30
20
10
0
ppm

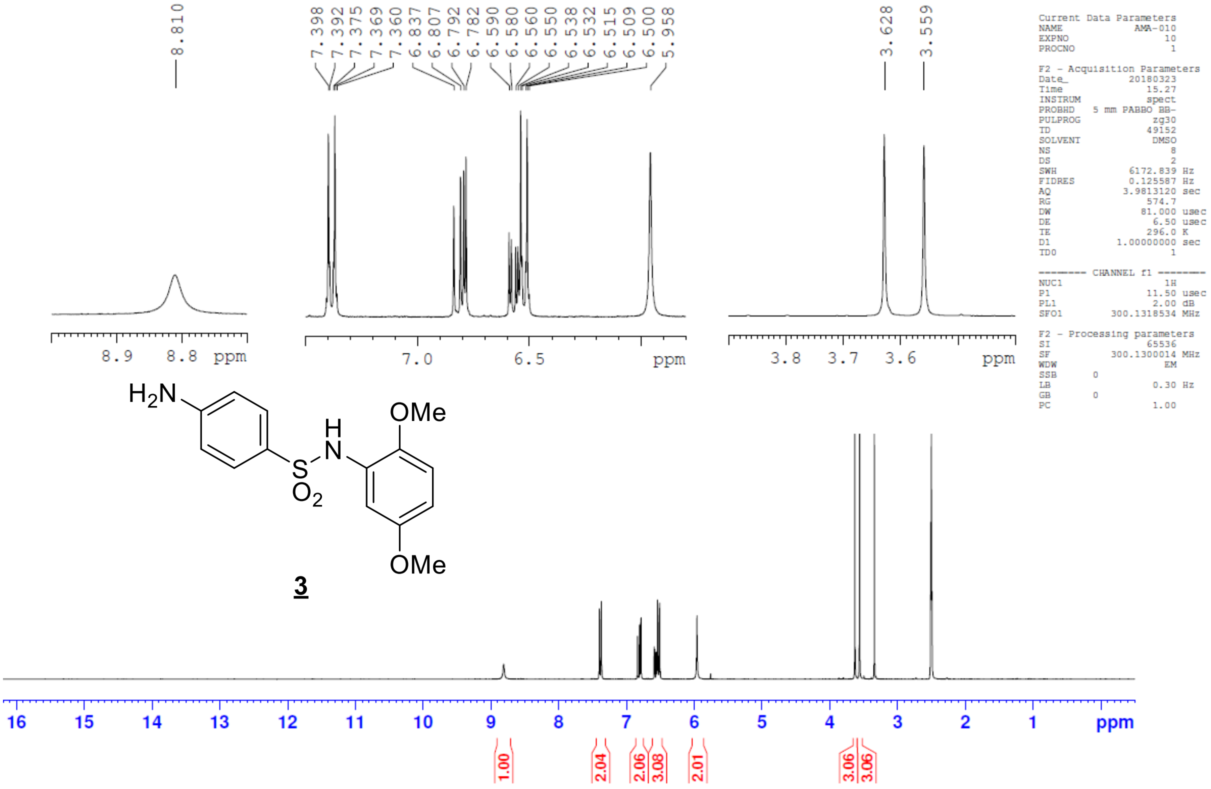

8.810
7.398 7.392 7.375 7.369 7.360 6.837 6.807 6.792 6.782 6.590 6.580 6.560 6.550 6.538 6.532 6.515 6.509 6.500 5.958
3.628 3.559
8.9 8.8 ppm
7.0 6.5 ppm
3.8 3.7 3.6 ppm
Current Data Parameters
NAME AMA-010
EXPNO 10
PROCNO 1
F2 - Acquisition Parameters
Date_ 20180323
Time 15.27
INSTRUM spect
PROBHD 5 mm PABBO BB-
PULPROG zg30
TD 49152
SOLVENT DMSO
NS 8
DS 2
SWH 6172.839 Hz
FIDRES 0.125587 Hz
AQ 3.9813120 sec
RG 574.7
DW 81.000 usec
DE 6.50 usec
TE 296.0 K
D1 1.00000000 sec
TD0 1
CHANNEL f1
NUC1 1H
P1 11.50 usec
PL1 2.00 dB
SFO1 300.1318534 MHz
F2 - Processing parameters
SI 65536
SF 300.1300014 MHz
WDW EM
SSB 0
LB 0.30 Hz
GB 0
PC 1.00
H2N
H
N
S
O2
OMe
OMe
3
16 15 14 13 12 11 10 9 8 7 6 5 4 3 2 1 ppm
1.00 2.04 2.06 3.08 2.01 3.06 3.06

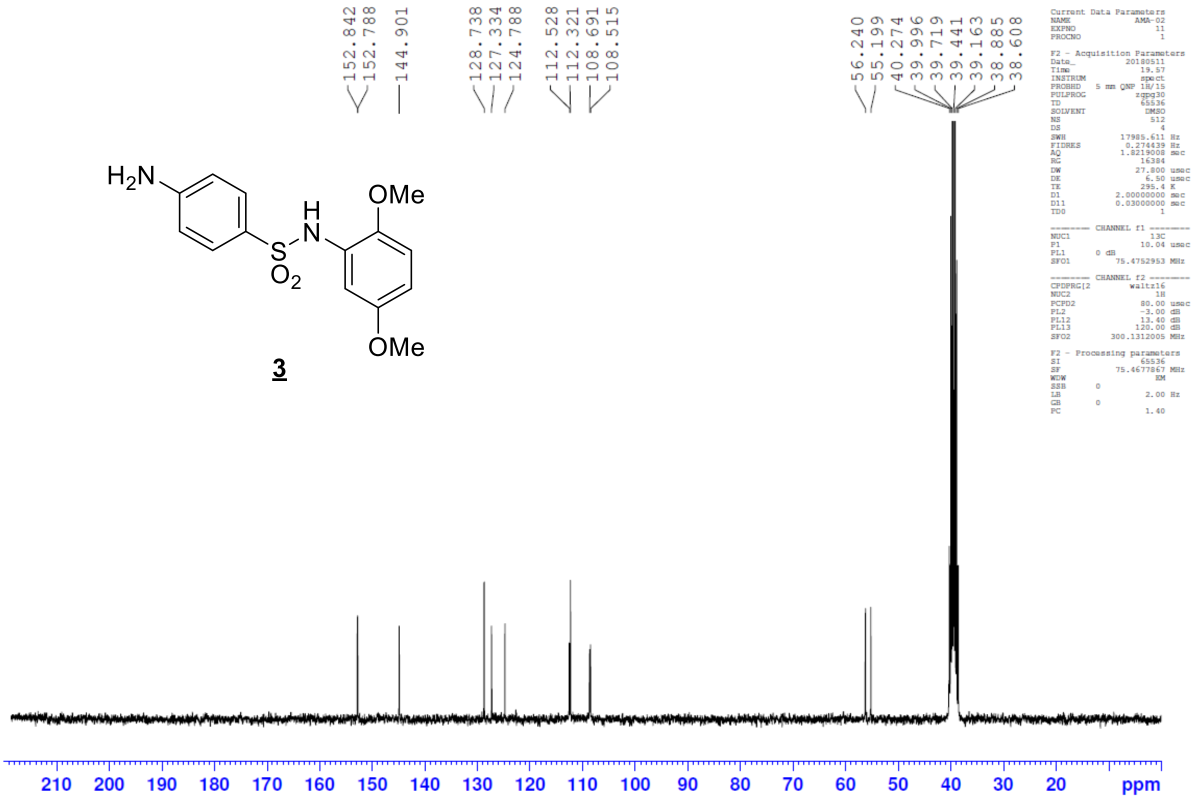

152.842 152.788 144.901 128.738 127.334 124.788 112.528 112.321 108.691 108.515
56.240 55.199 40.274 39.996 39.719 39.441 39.163 38.885 38.608
H2N
H
N
S
O2
OMe
OMe
3
210 200 190 180 170 160 150 140 130 120 110 100 90 80 70 60 50 40 30 20 ppm

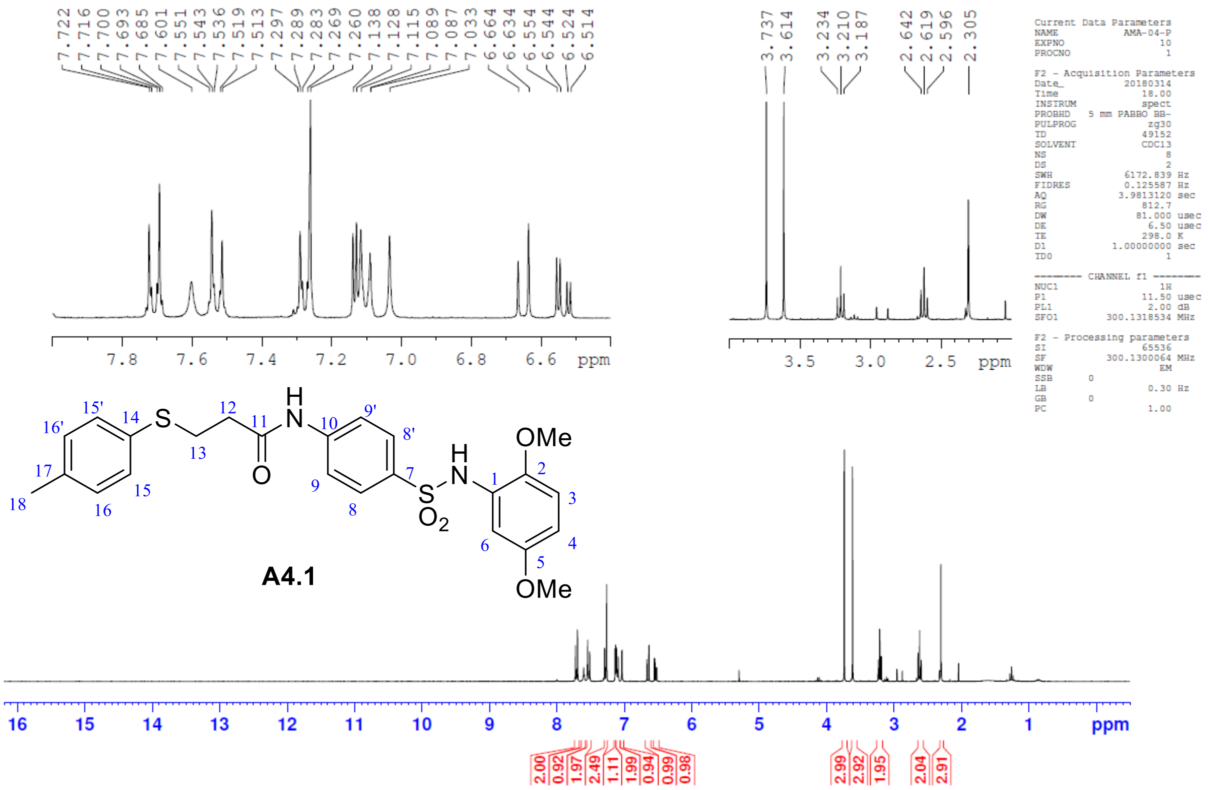
7.722 7.716 7.700 7.693 7.685 7.601 7.551 7.543 7.536 7.519 7.513 7.297 7.289 7.283 7.269 7.260 7.138 7.128 7.115 7.089 7.087 7.033 6.664 6.634 6.554 6.544 6.524 6.514
3.737 3.614 3.234 3.210 3.187 2.642 2.619 2.596 2.305
Current Data Parameters
NAME AMA-04-P
EXPNO 10
PROCNO 1
F2 - Acquisition Parameters
Date_ 20180314
Time 18.00
INSTRUM spect
PROBHD 5 mm PABBO BB-
PULPROG zg30
TD 49152
SOLVENT CDCl3
NS 8
DS 2
SWH 6172.839 Hz
FIDRES 0.125587 Hz
AQ 3.9813120 sec
RG 812.7
DW 81.000 usec
DE 6.50 usec
TE 298.0 K
D1 1.00000000 sec
TD0 1
CHANNEL f1
NUC1 1H
P1 11.50 usec
PL1 2.00 dB
SFO1 300.1318534 MHz
F2 - Processing parameters
SI 65536
SF 300.1300064 MHz
WDW EM
SSB 0
LB 0.30 Hz
GB 0
PC 1.00
A4.1
OMe
OMe
2.00 0.92 1.97 2.49 1.11 1.99 0.94 0.99 0.98 2.99 2.92 1.95 2.04 2.91
ppm

MJR-59-p2c2

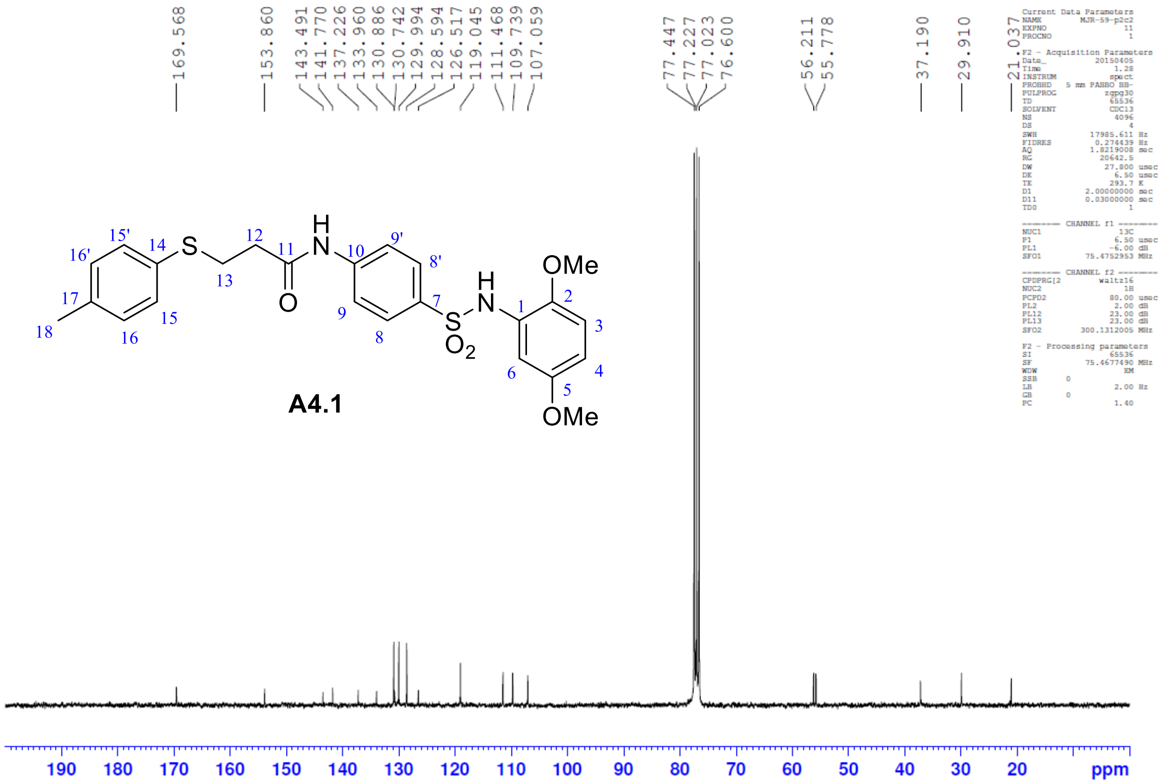
169.568 153.860 143.491 141.770 137.226 133.960 130.886 130.742 129.994 128.594 126.517 119.045 111.468 109.739 107.059 77.447 77.227 77.023 76.600 56.211 55.778 37.190 29.910 21.037
A4.1
OMe
OMe
ppm

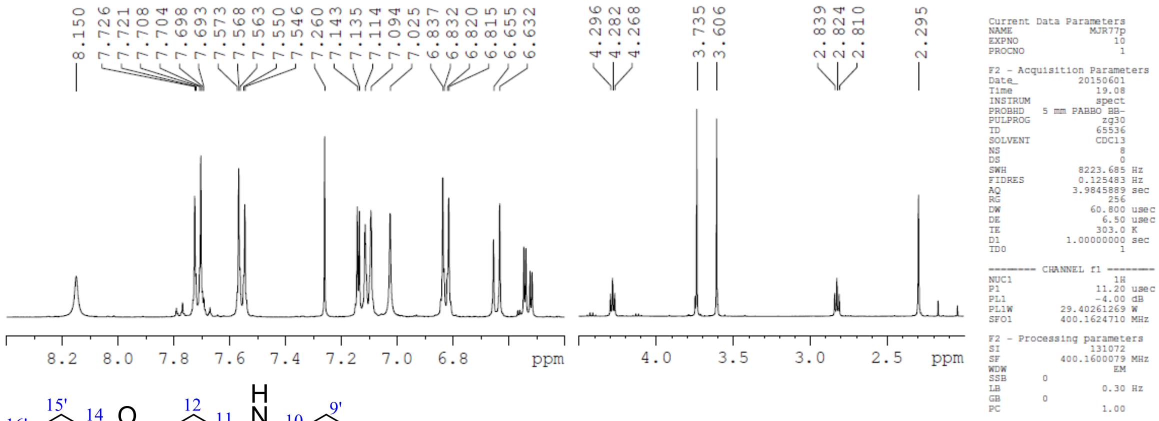
8.150
7.726 7.721 7.708 7.704 7.698 7.693 7.573 7.568 7.563 7.550 7.546 7.260 7.143 7.135 7.114 7.094 7.025 6.837 6.832 6.820 6.815 6.655 6.632
4.296 4.282 4.268
3.735 3.606
2.839 2.824 2.810
2.295
8.2 8.0 7.8 7.6 7.4 7.2 7.0 6.8 ppm
4.0 3.5 3.0 2.5 ppm
Current Data Parameters
NAME MJR77p
EXPNO 10
PROCNO 1
F2 - Acquisition Parameters
Date_ 20150601
Time 19.08
INSTRUM spect
PROBHD 5 mm PABBO BB-
PULPROG zg30
TD 65536
SOLVENT CDCl3
NS 8
DS 0
SWH 8223.685 Hz
FIDRES 0.125483 Hz
AQ 3.9845889 sec
RG 256
DW 60.800 usec
DE 6.50 usec
TE 303.0 K
D1 1.00000000 sec
TD0 1
======== CHANNEL f1 ========
NUC1 1H
P1 11.20 usec
PL1 -4.00 dB
PL1W 29.40261269 W
SFO1 400.1624710 MHz
F2 - Processing parameters
SI 131072
SF 400.1600079 MHz
WDW EM
SSB 0
LB 0.30 Hz
GB 0
PC 1.00

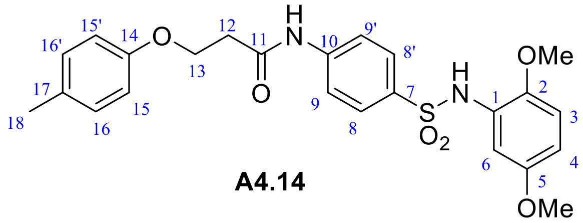

A4.14

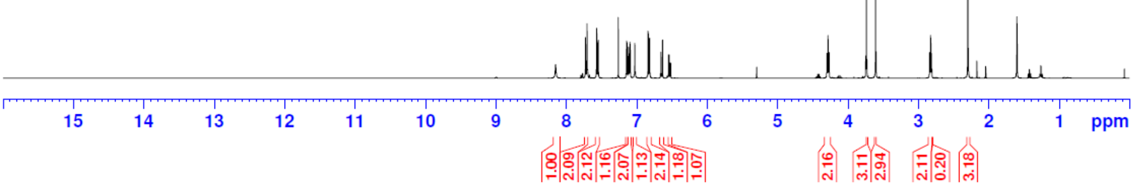
15 14 13 12 11 10 9 8 7 6 5 4 3 2 1 ppm
1.00 2.09 2.12 1.16 2.07 1.13 2.14 1.18 1.07
2.16 3.11 2.94 2.11 0.20 3.18

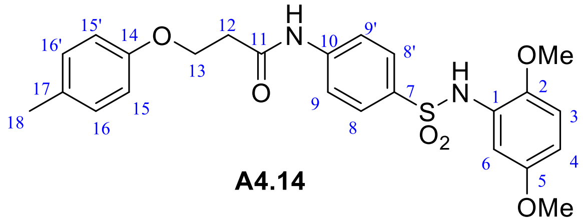

A4.14

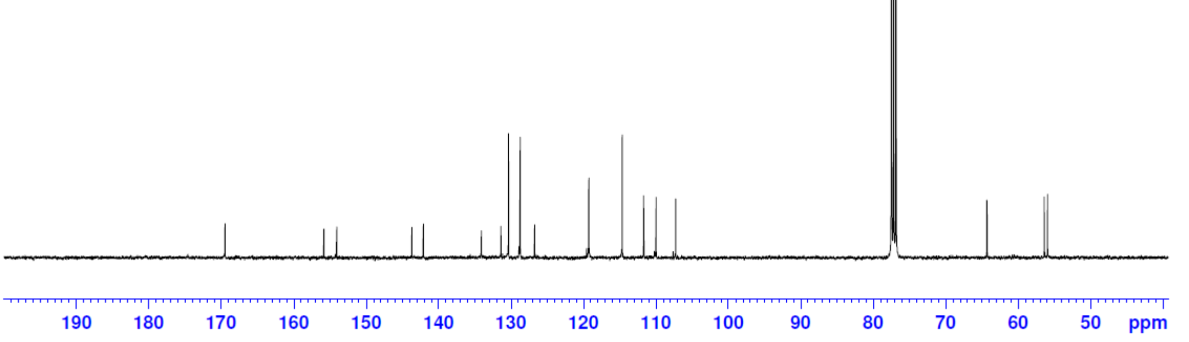
169.484
155.836 154.062
143.687 142.113
134.091 131.396 130.341 128.741 126.747
119.260
114.653 111.689 109.969 107.264
64.285
56.378
190 180 170 160 150 140 130 120 110 100 90 80 70 60 50 ppm

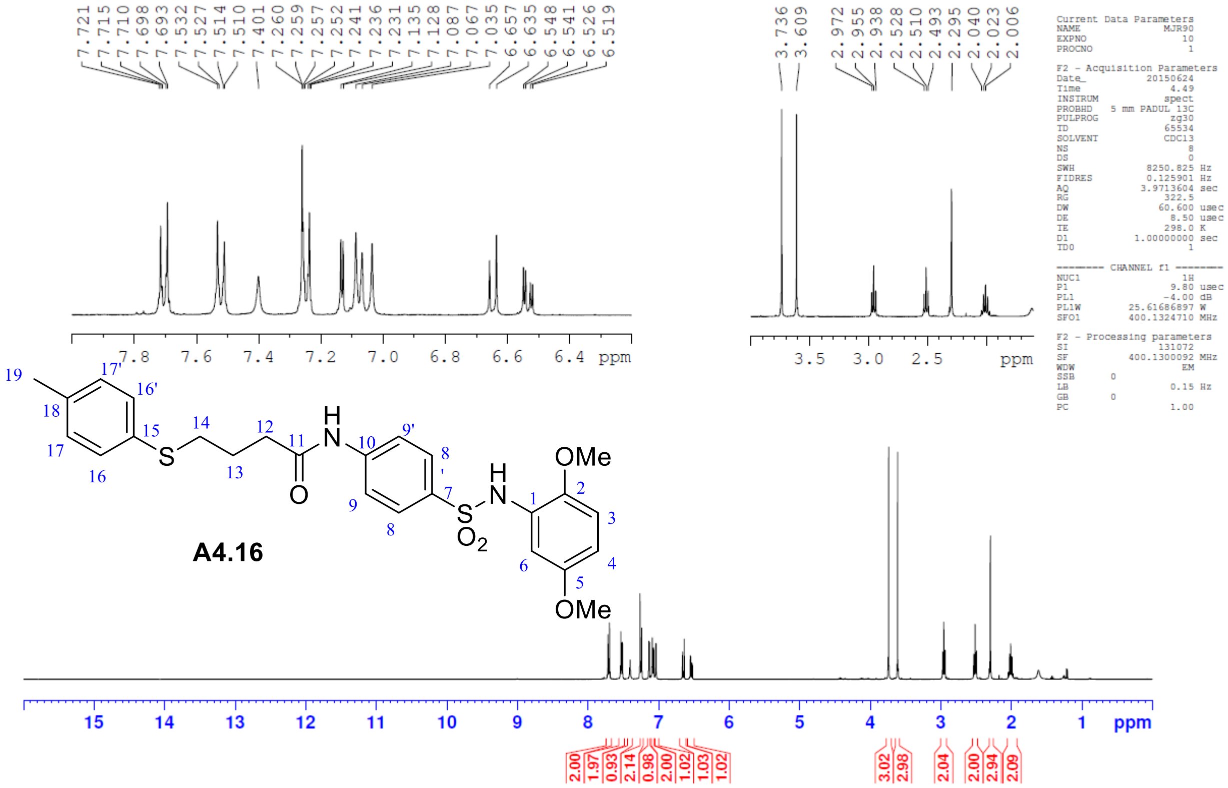
7.721 7.715 7.710 7.698 7.693 7.532 7.527 7.514 7.510 7.401 7.260 7.259 7.257 7.252 7.241 7.236 7.231 7.135 7.128 7.087 7.067 7.035 6.657 6.635 6.548 6.541 6.526 6.519
3.736 3.609 2.972 2.955 2.938 2.528 2.510 2.493 2.295 2.040 2.023 2.006
A4.16
OMe
OMe
2.00 1.97 0.93 2.14 0.98 2.00 1.02 1.03 1.02 3.02 2.98 2.04 2.00 2.94 2.09
ppm

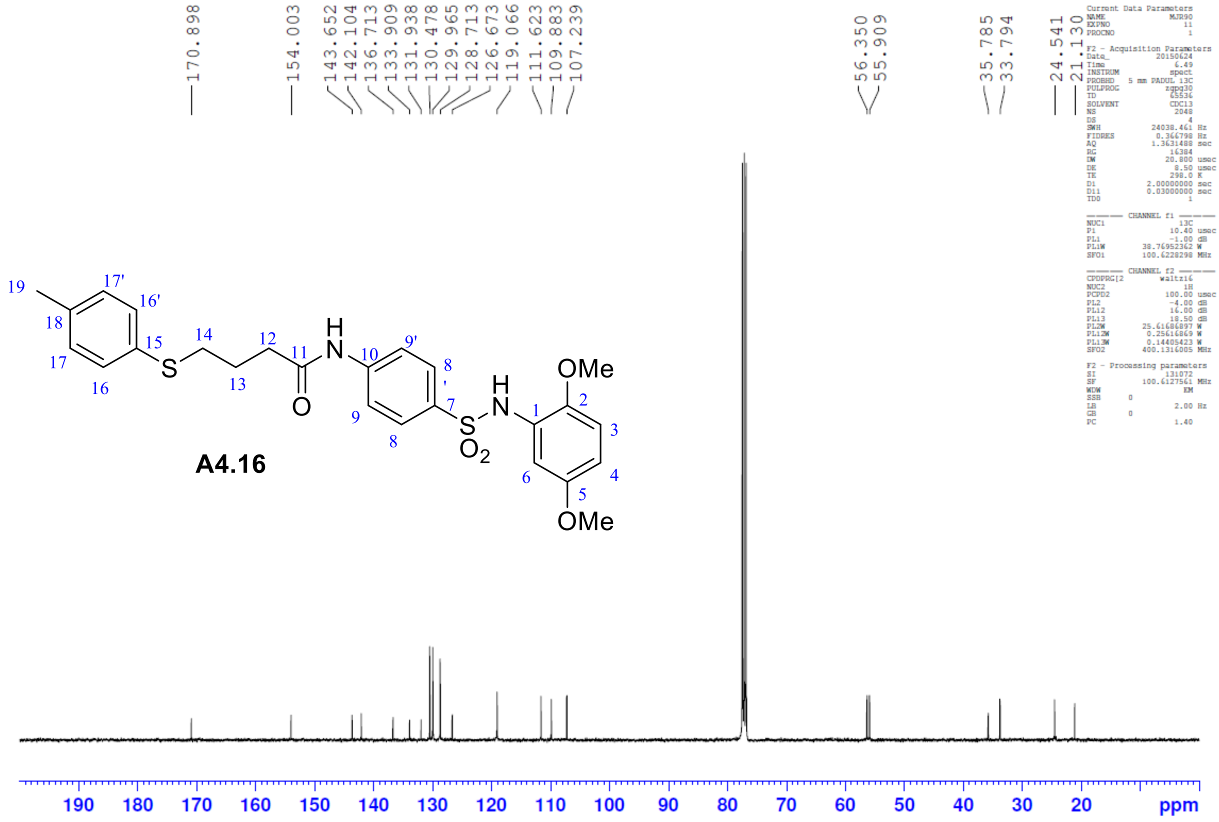
170.898
154.003
143.652 142.104 136.713 133.909 131.938 130.478 129.965 128.713 126.673 119.066 111.623 109.883 107.239
56.350 55.909
35.785 33.794
24.541 21.130
A4.16
OMe
OMe
ppm

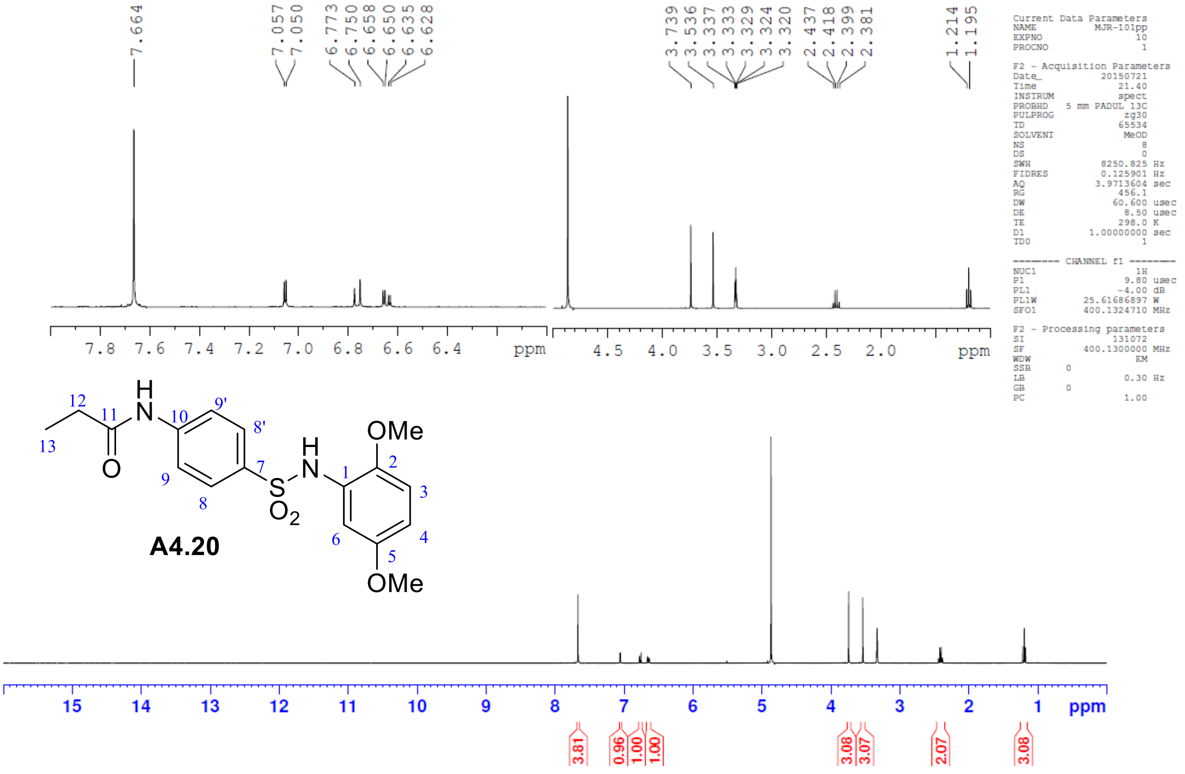
7.664
7.057
7.050
6.773
6.750
6.658
6.650
6.635
6.628
3.739
3.536
3.337
3.333
3.329
3.324
3.320
2.437
2.418
2.399
2.381
1.214
1.195
Current Data Parameters
NAME MJR-101pp
EXPNO 10
PROCNO 1
F2 - Acquisition Parameters
Date_ 20150721
Time 21.40
INSTRUM spect
PROBHD 5 mm PADUL 13C
PULPROG zg30
TD 65534
SOLVENT MeOD
NS 8
DS 0
SWH 8250.825 Hz
FIDRES 0.125901 Hz
AQ 3.9713604 sec
RG 456.1
DW 60.600 usec
DE 8.50 usec
TE 298.0 K
D1 1.00000000 sec
TD0 1
======== CHANNEL f1 ========
NUC1 1H
P1 9.80 usec
PL1 -4.00 dB
PL1W 25.61686897 W
SFO1 400.1324710 MHz
F2 - Processing parameters
SI 131072
SF 400.1300000 MHz
WDW EM
SSB 0
LB 0.30 Hz
GB 0
PC 1.00
7.8 7.6 7.4 7.2 7.0 6.8 6.6 6.4 ppm
4.5 4.0 3.5 3.0 2.5 2.0 ppm
OMe
OMe
A4.20
15 14 13 12 11 10 9 8 7 6 5 4 3 2 1 ppm
3.81
0.96
1.00
1.00
3.08
3.07
2.07
3.08

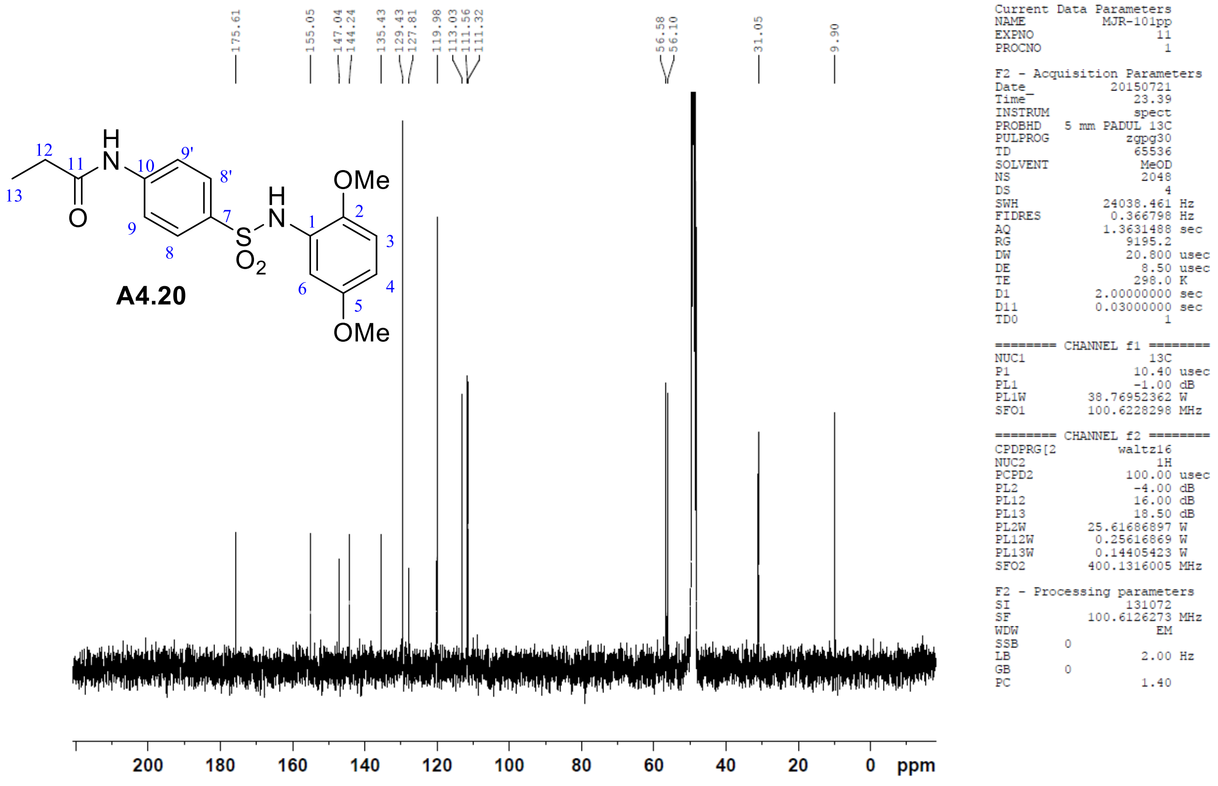
175.61
155.05
147.04
144.24
135.43
129.43
127.81
119.98
113.03
111.56
111.32
56.58
56.10
31.05
9.90
Current Data Parameters
NAME MJR-101pp
EXPNO 11
PROCNO 1
F2 - Acquisition Parameters
Date_ 20150721
Time 23.39
INSTRUM spect
PROBHD 5 mm PADUL 13C
PULPROG zgpg30
TD 65536
SOLVENT MeOD
NS 2048
DS 4
SWH 24038.461 Hz
FIDRES 0.366798 Hz
AQ 1.3631488 sec
RG 9195.2
DW 20.800 usec
DE 8.50 usec
TE 298.0 K
D1 2.00000000 sec
D11 0.03000000 sec
TD0 1
======== CHANNEL f1 ========
NUC1 13C
P1 10.40 usec
PL1 -1.00 dB
PL1W 38.76952362 W
SFO1 100.6228298 MHz
======== CHANNEL f2 ========
CPDPRG[2 waltz16
NUC2 1H
PCPD2 100.00 usec
PL2 -4.00 dB
PL12 16.00 dB
PL13 18.50 dB
PL2W 25.61686897 W
PL12W 0.25616869 W
PL13W 0.14405423 W
SFO2 400.1316005 MHz
F2 - Processing parameters
SI 131072
SF 100.6126273 MHz
WDW EM
SSB 0
LB 2.00 Hz
GB 0
PC 1.40
OMe
OMe
A4.20
200 180 160 140 120 100 80 60 40 20 0 ppm

*NMR spectra of the compounds **A4.15***

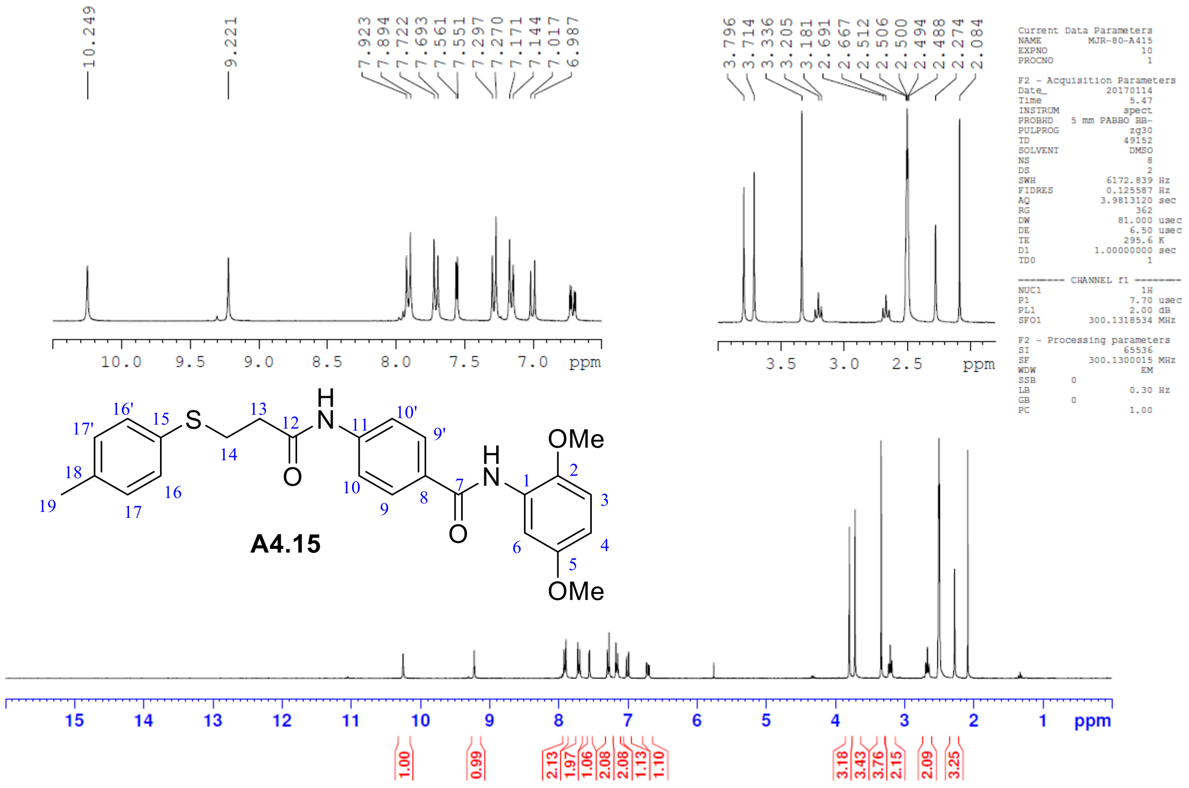

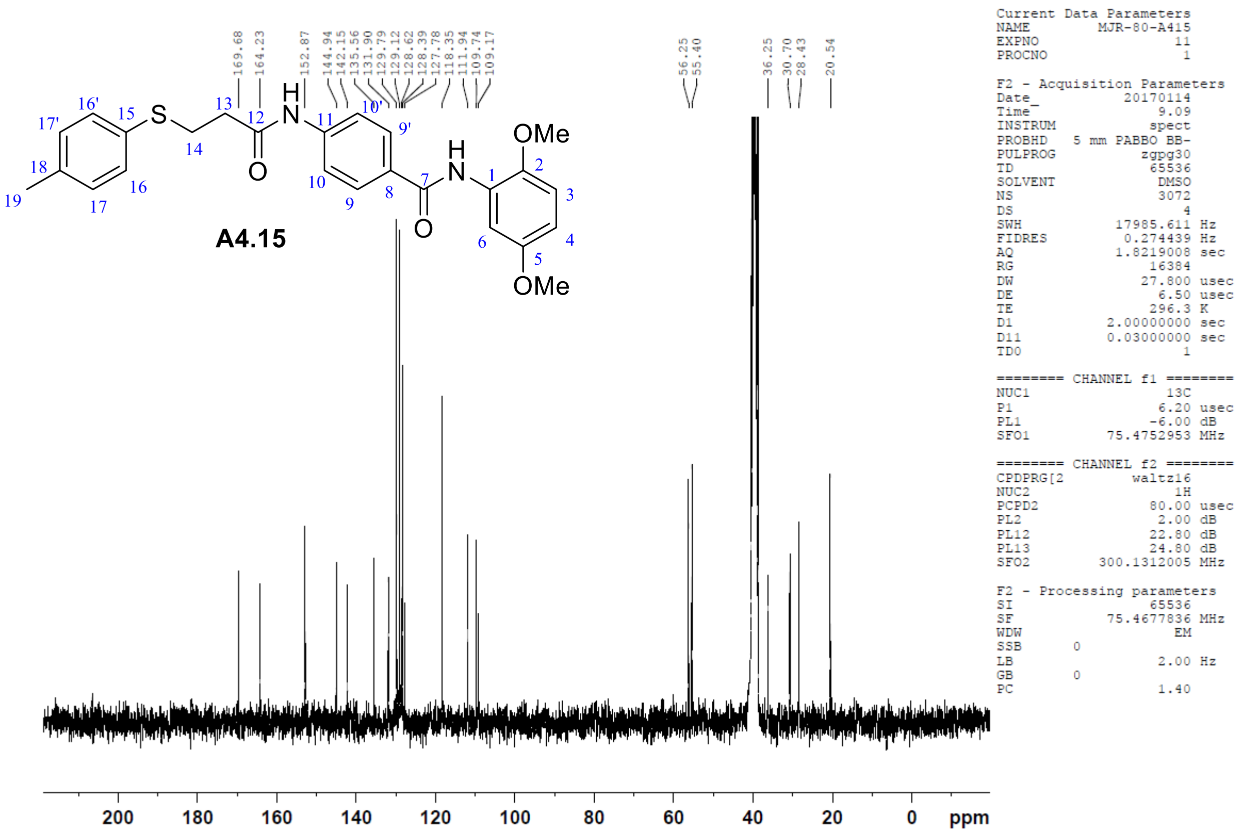

NMR spectra of the intermediates ***6****,* ***7****,* ***d₃-8****,* ***d₃-1****,* ***d₃-2****,* ***d₃-3*** *and final compounds* ***d₃-A4.1***

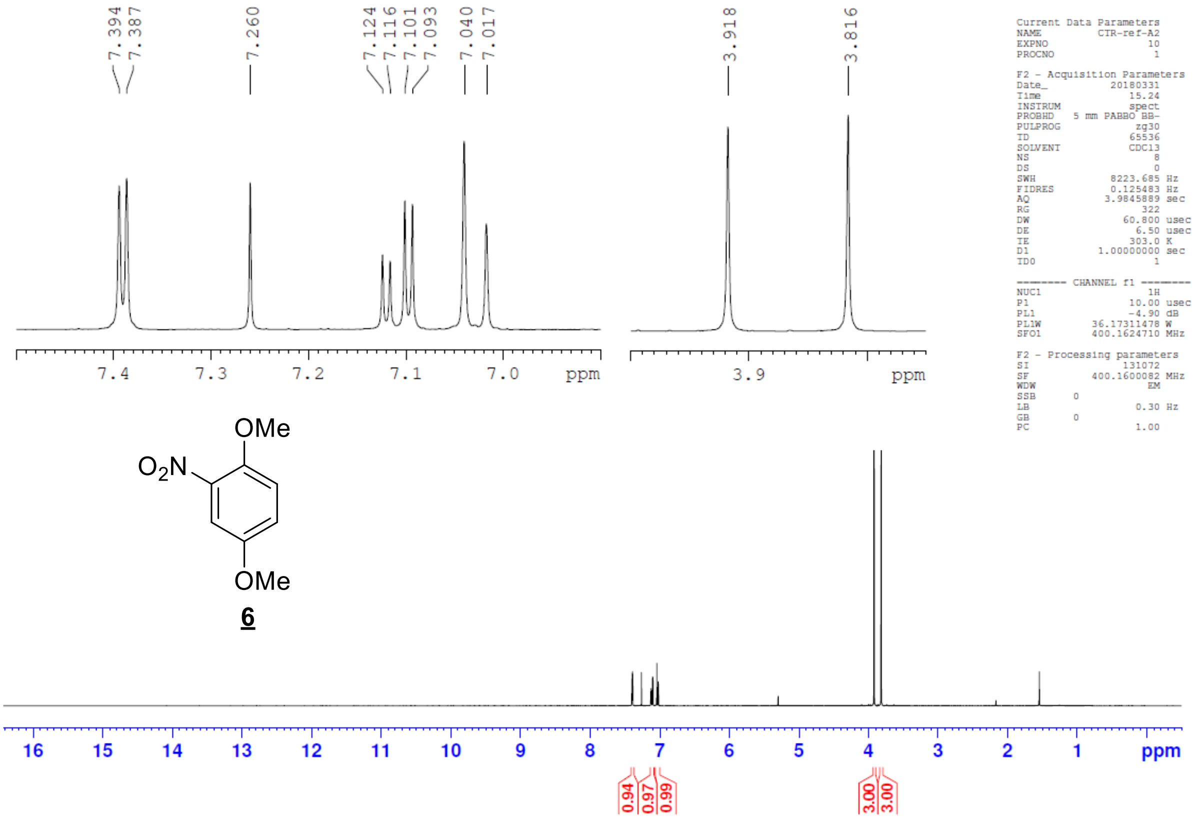

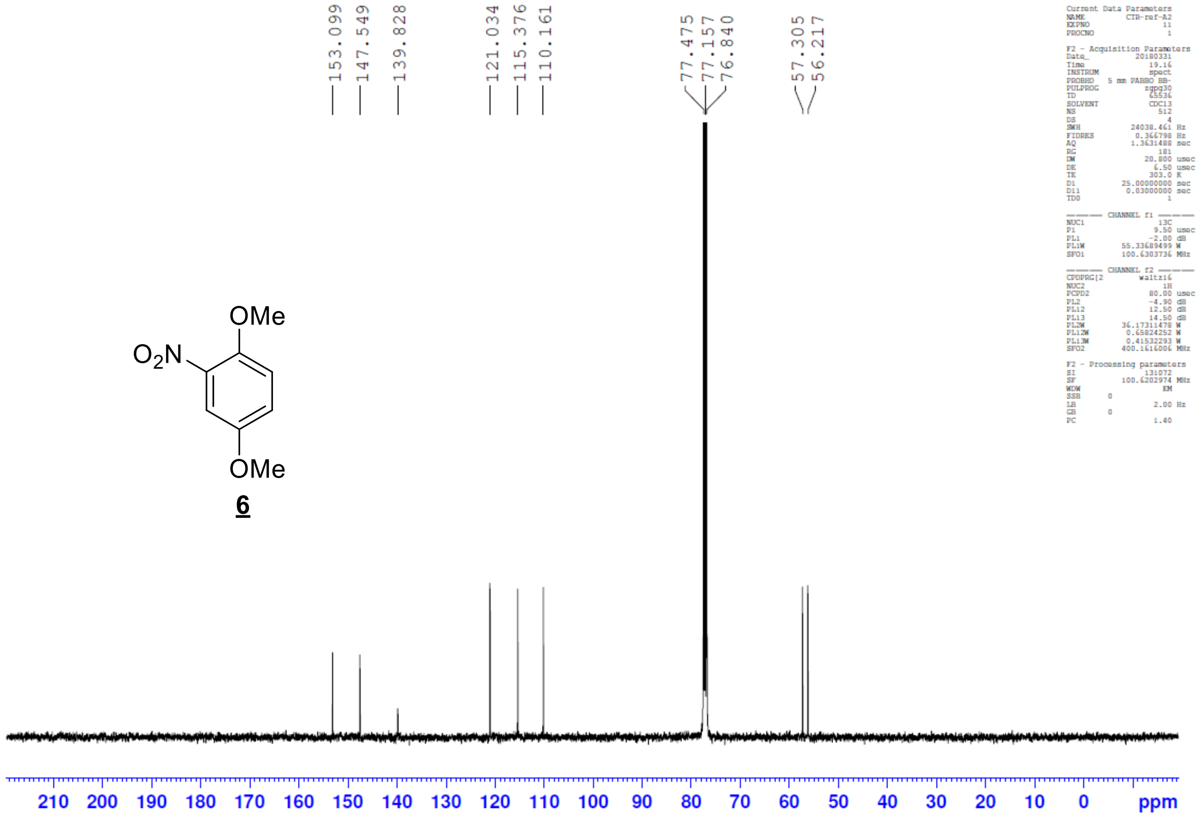

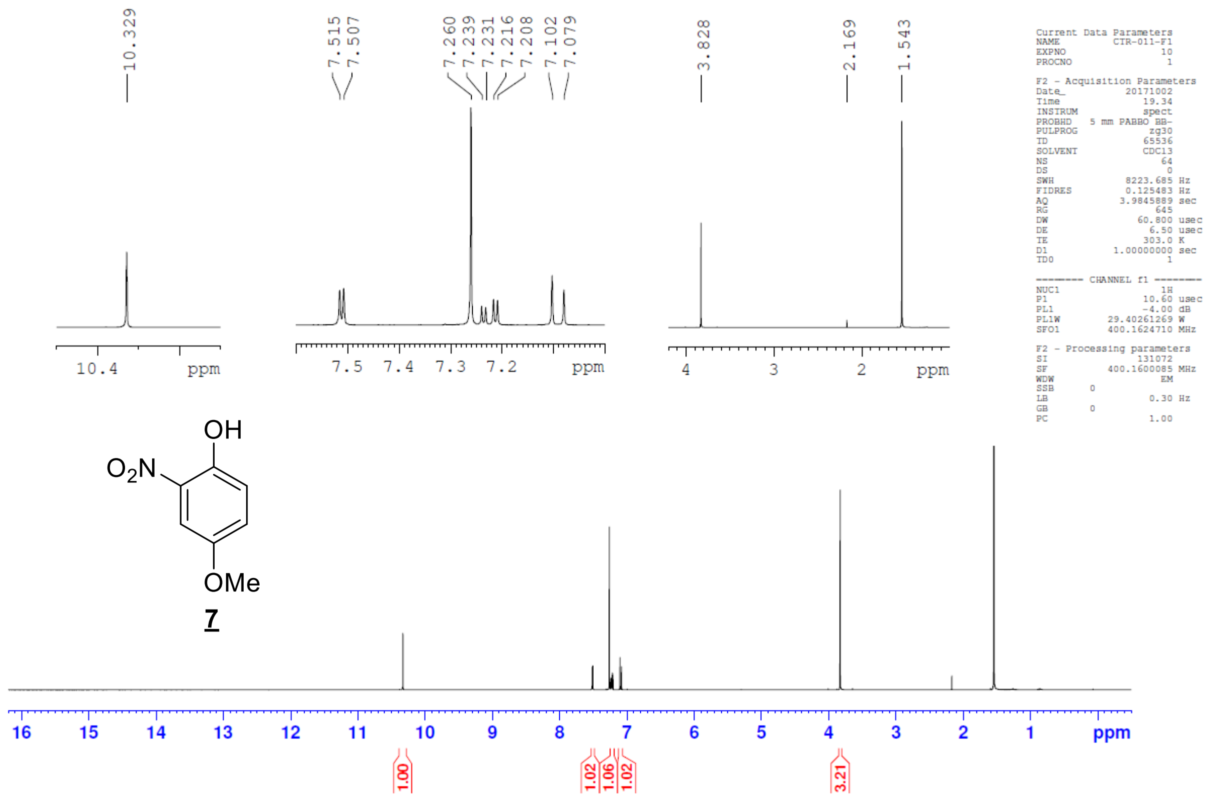

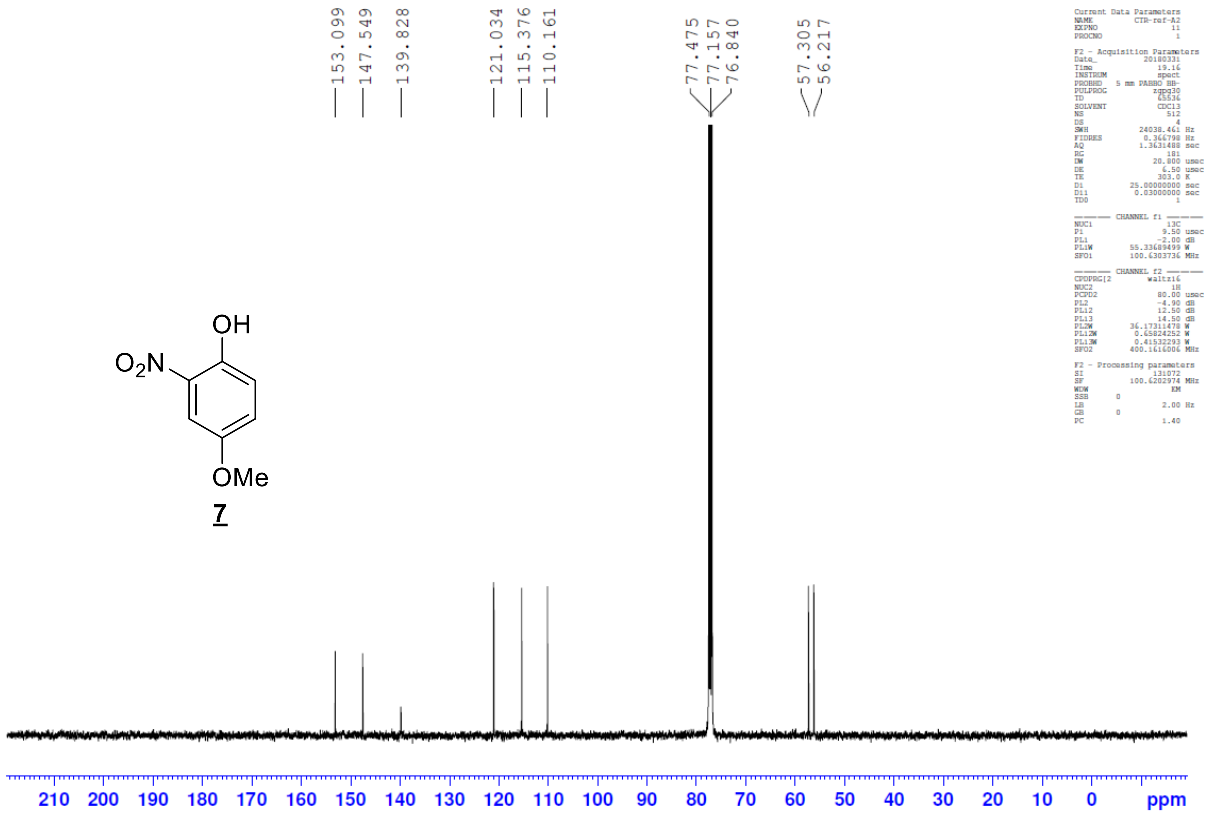

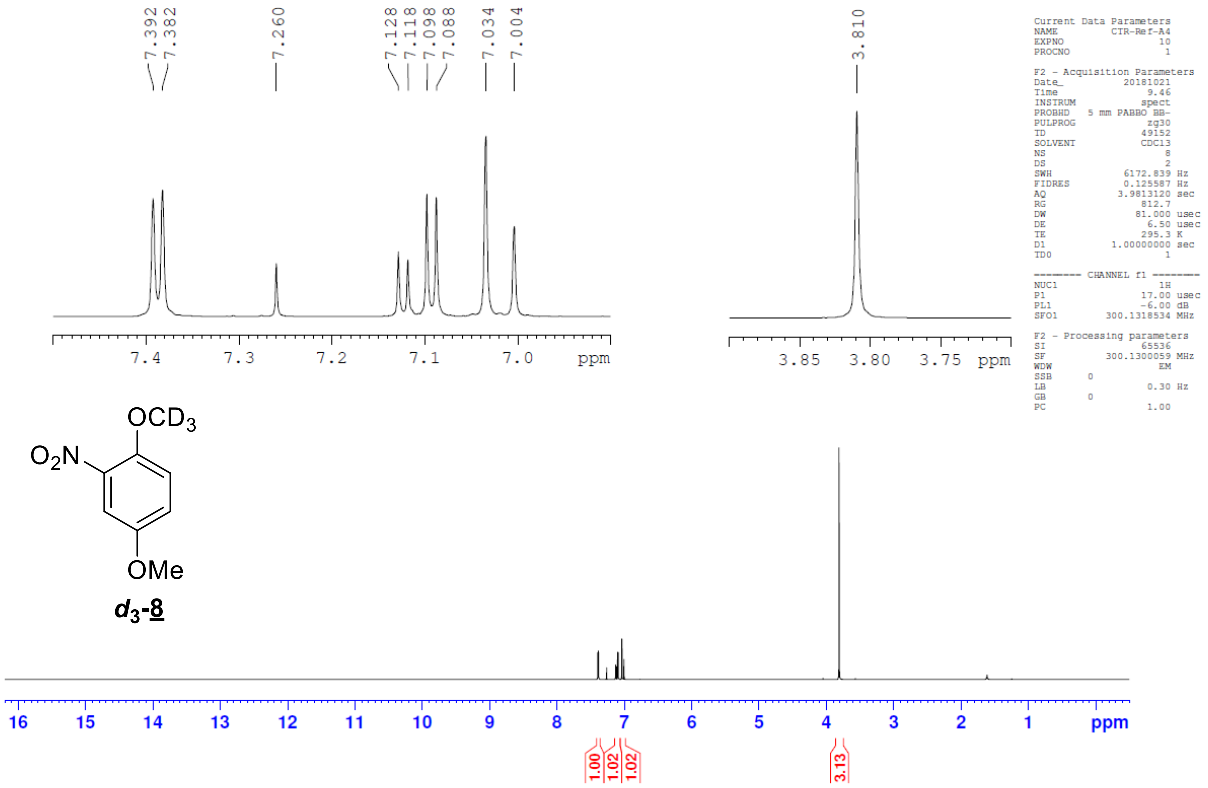
7.392
7.382
7.260
7.128
7.118
7.098
7.088
7.034
7.004
3.810
Current Data Parameters
NAME CTR-Ref-A4
EXPNO 10
PROCNO 1
F2 - Acquisition Parameters
Date_ 20181021
Time 9.46
INSTRUM spect
PROBHD 5 mm PABBO BB-
PULPROG zg30
TD 49152
SOLVENT CDCl3
NS 8
DS 2
SWH 6172.839 Hz
FIDRES 0.125587 Hz
AQ 3.9813120 sec
RG 812.7
DW 81.000 usec
DE 6.50 usec
TE 295.3 K
D1 1.00000000 sec
TD0 1
CHANNEL f1
NUC1 1H
P1 17.00 usec
PL1 -6.00 dB
SFO1 300.1318534 MHz
F2 - Processing parameters
SI 65536
SF 300.1300059 MHz
WDW EM
SSB 0
LB 0.30 Hz
GB 0
PC 1.00
OCD3
O2N
OMe
ppm
1.00
1.02
1.02
3.13

*d*3-8

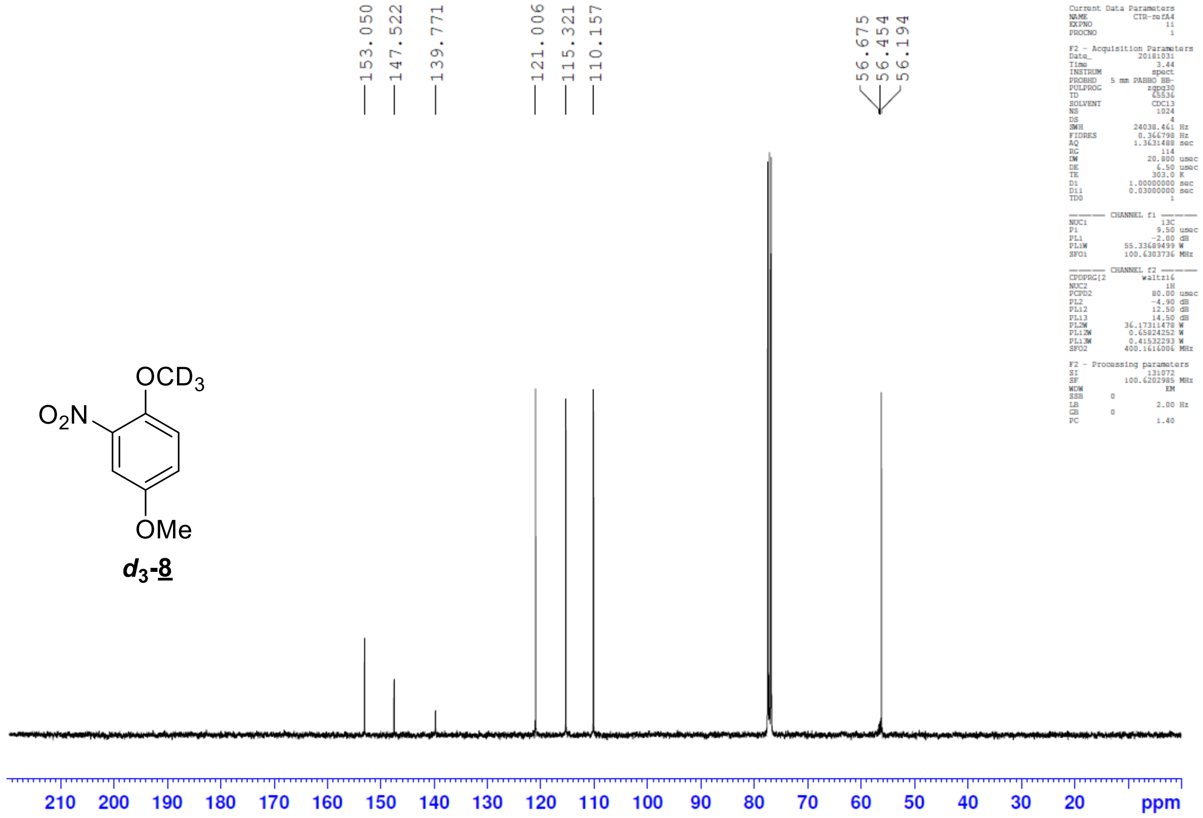
153.050
147.522
139.771
121.006
115.321
110.157
56.675
56.454
56.194
OCD3
O2N
OMe
ppm

*d*3-8

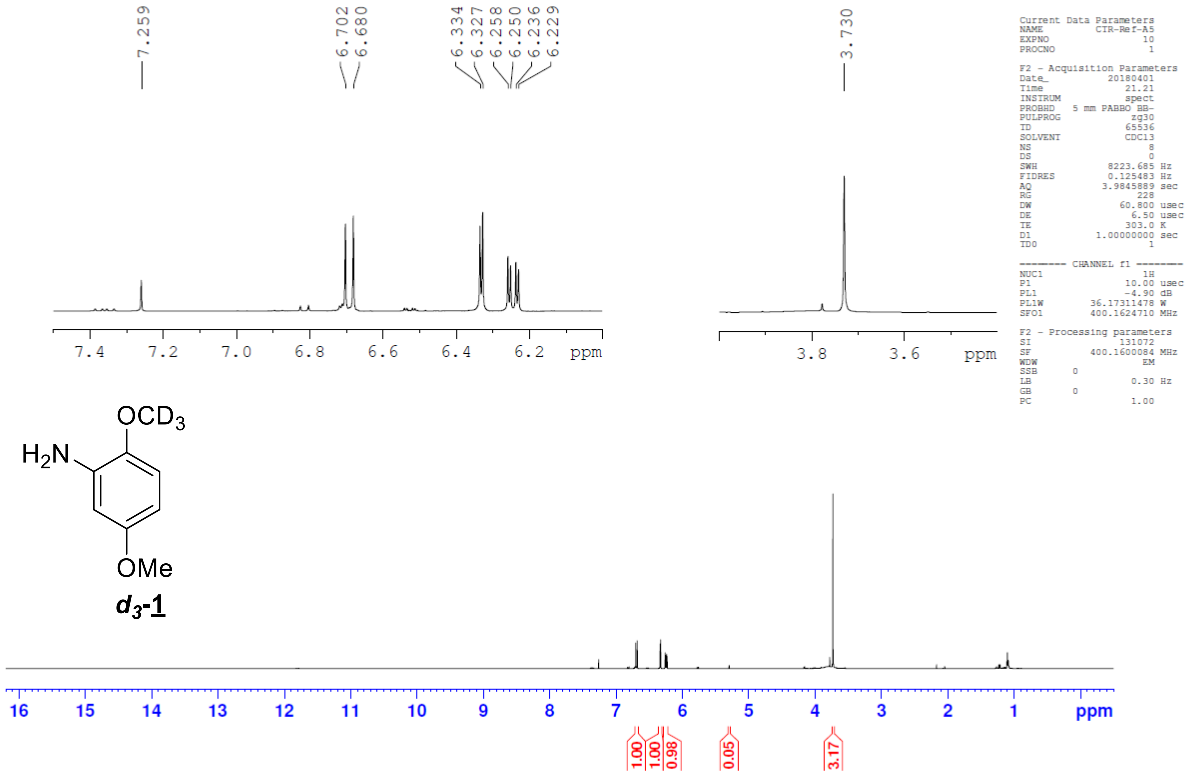
7.259
6.702
6.680
6.334
6.327
6.258
6.250
6.236
6.229
3.730
7.4 7.2 7.0 6.8 6.6 6.4 6.2 ppm
3.8 3.6 ppm
Current Data Parameters
NAME CTR-Ref-A5
EXPNO 10
PROCNO 1
F2 - Acquisition Parameters
Date_ 20180401
Time 21.21
INSTRUM spect
PROBHD 5 mm PABBO BB-
PULPROG zg30
TD 65536
SOLVENT CDCl3
NS 8
DS 0
SWH 8223.685 Hz
FIDRES 0.125483 Hz
AQ 3.9845889 sec
RG 228
DW 60.800 usec
DE 6.50 usec
TE 303.0 K
D1 1.00000000 sec
TD0 1
======== CHANNEL f1 ========
NUC1 1H
P1 10.00 usec
PL1 -4.90 dB
PL1W 36.17311478 W
SFO1 400.1624710 MHz
F2 - Processing parameters
SI 131072
SF 400.1600084 MHz
WDW EM
SSB 0
LB 0.30 Hz
GB 0
PC 1.00
OCD3
H2N
OMe
16 15 14 13 12 11 10 9 8 7 6 5 4 3 2 1 ppm
1.00
1.00
0.98
0.05
3.17

***d*$_3$-1**

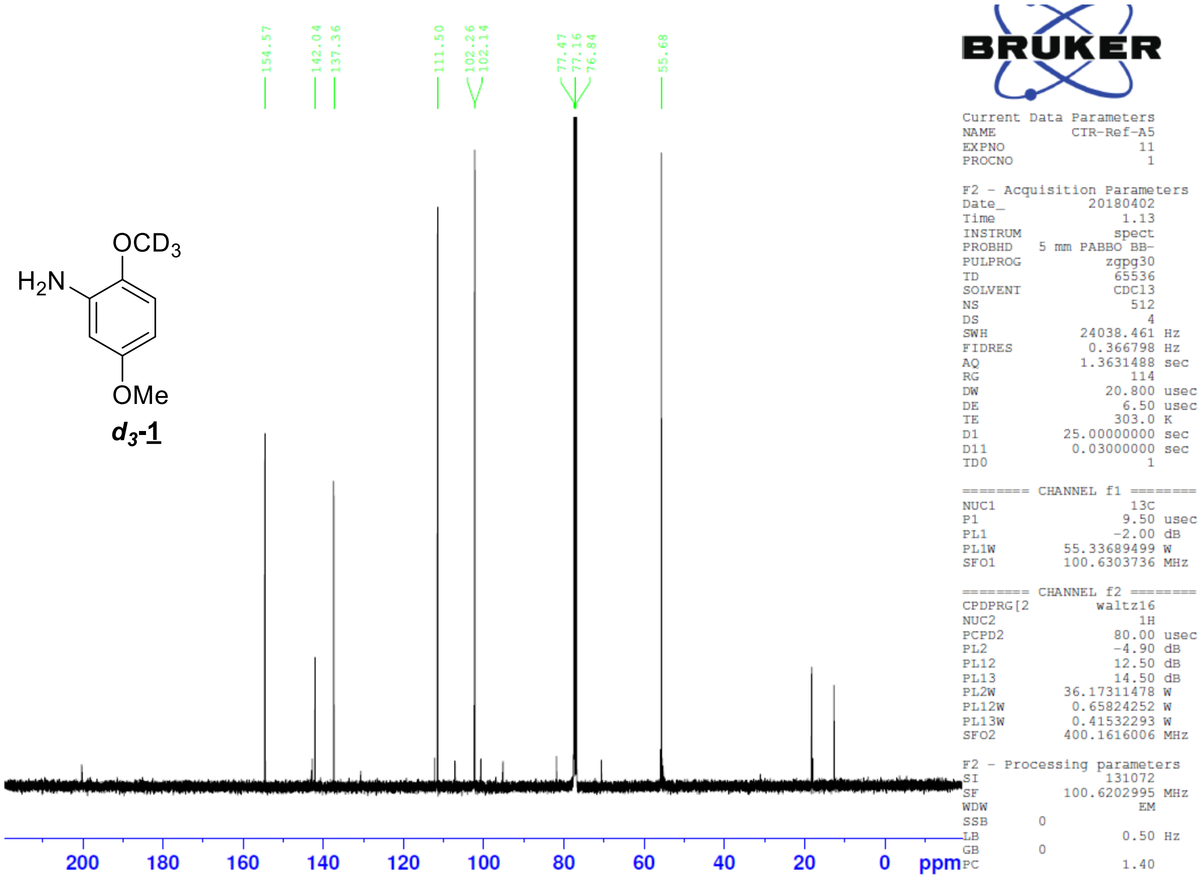
154.57
142.04
137.36
111.50
102.26
102.14
77.47
77.16
76.84
55.68
BRUKER
Current Data Parameters
NAME CTR-Ref-A5
EXPNO 11
PROCNO 1
F2 - Acquisition Parameters
Date_ 20180402
Time 1.13
INSTRUM spect
PROBHD 5 mm PABBO BB-
PULPROG zgpg30
TD 65536
SOLVENT CDCl3
NS 512
DS 4
SWH 24038.461 Hz
FIDRES 0.366798 Hz
AQ 1.3631488 sec
RG 114
DW 20.800 usec
DE 6.50 usec
TE 303.0 K
D1 25.00000000 sec
D11 0.03000000 sec
TD0 1
======== CHANNEL f1 ========
NUC1 13C
P1 9.50 usec
PL1 -2.00 dB
PL1W 55.33689499 W
SFO1 100.6303736 MHz
======== CHANNEL f2 ========
CPDPRG[2 waltz16
NUC2 1H
PCPD2 80.00 usec
PL2 -4.90 dB
PL12 12.50 dB
PL13 14.50 dB
PL2W 36.17311478 W
PL12W 0.65824252 W
PL13W 0.41532293 W
SFO2 400.1616006 MHz
F2 - Processing parameters
SI 131072
SF 100.6202995 MHz
WDW EM
SSB 0
LB 0.50 Hz
GB 0
PC 1.40
OCD3
H2N
OMe
200 180 160 140 120 100 80 60 40 20 0 ppm

***d*$_3$-1**

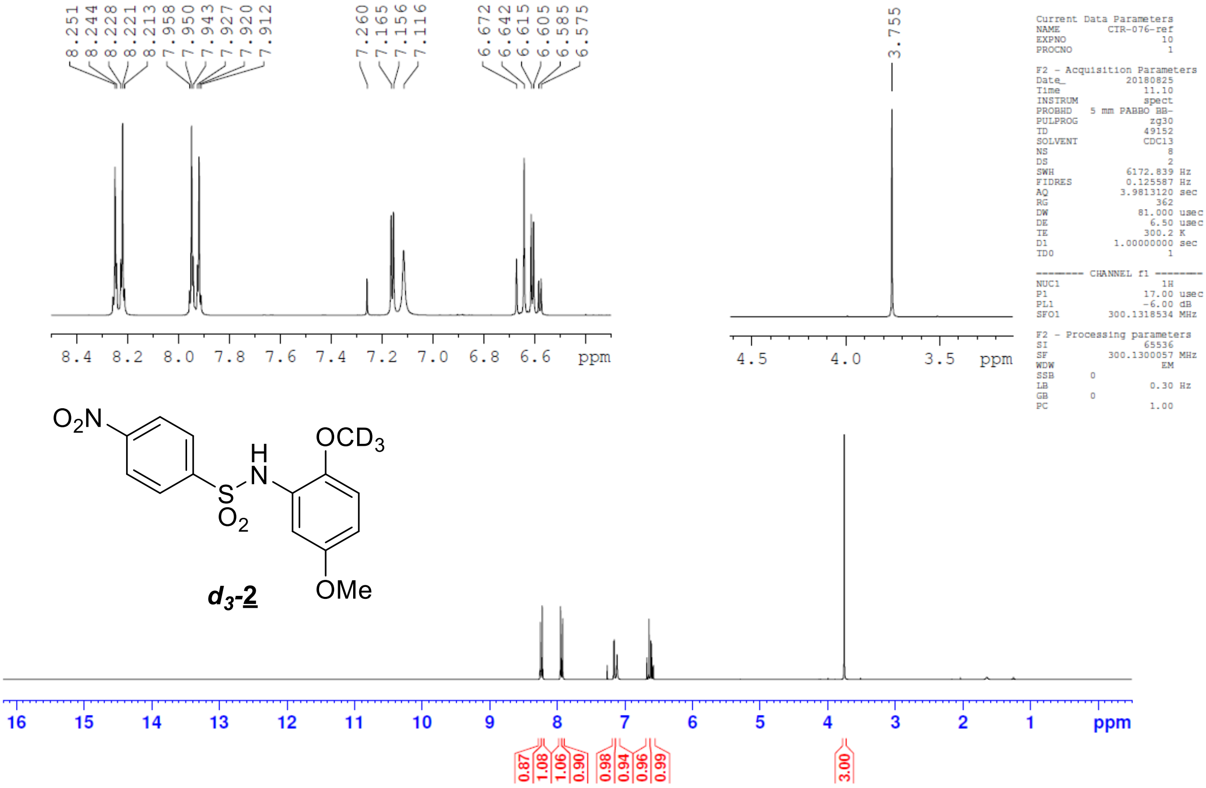
8.251
8.244
8.228
8.221
8.213
7.958
7.950
7.943
7.927
7.920
7.912
7.260
7.165
7.156
7.116
6.672
6.642
6.615
6.605
6.585
6.575
3.755
ppm
O2N
H
N
S
O2
OCD3
OMe
d3-2
0.87
1.08
1.06
0.90
0.98
0.94
0.96
0.99
3.00

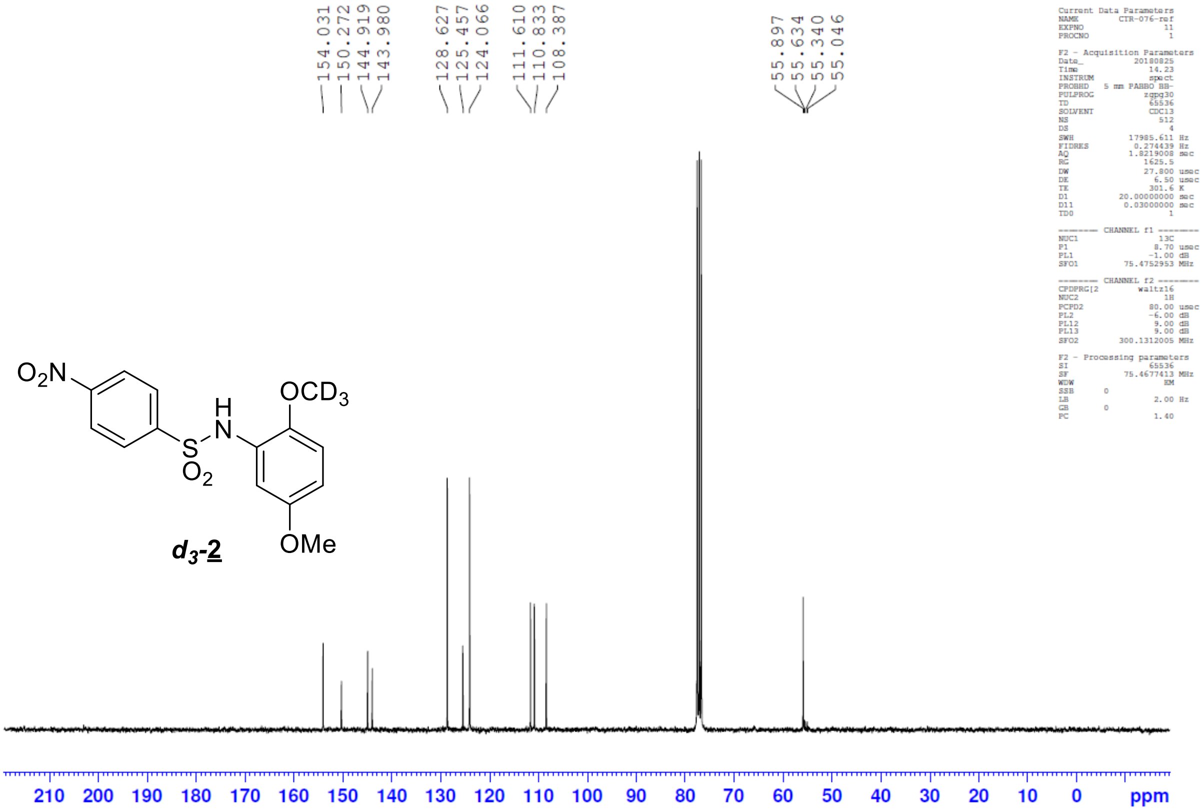
154.031
150.272
144.919
143.980
128.627
125.457
124.066
111.610
110.833
108.387
55.897
55.634
55.340
55.046
O2N
H
N
S
O2
OCD3
OMe
d3-2
ppm

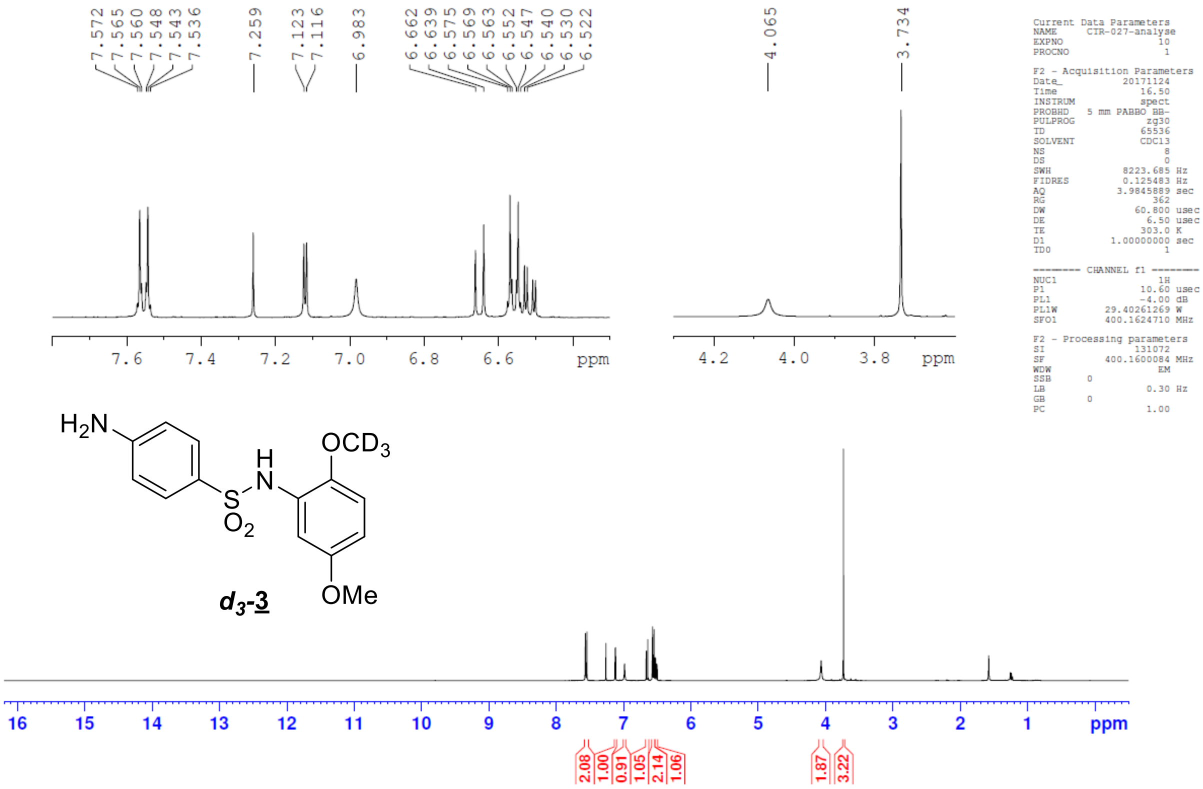
7.572
7.565
7.560
7.548
7.543
7.536
7.259
7.123
7.116
6.983
6.662
6.639
6.575
6.569
6.563
6.552
6.547
6.540
6.530
6.522
4.065
3.734
Current Data Parameters
NAME CTR-027-analyse
EXPNO 10
PROCNO 1
F2 - Acquisition Parameters
Date_ 20171124
Time 16.50
INSTRUM spect
PROBHD 5 mm PABBO BB-
PULPROG zg30
TD 65536
SOLVENT CDCl3
NS 8
DS 0
SWH 8223.685 Hz
FIDRES 0.125483 Hz
AQ 3.9845889 sec
RG 362
DW 60.800 usec
DE 6.50 usec
TE 303.0 K
D1 1.00000000 sec
TD0 1
======== CHANNEL f1 ========
NUC1 1H
P1 10.60 usec
PL1 -4.00 dB
PL1W 29.40261269 W
SFO1 400.1624710 MHz
F2 - Processing parameters
SI 131072
SF 400.1600084 MHz
WDW EM
SSB 0
LB 0.30 Hz
GB 0
PC 1.00
H2N
H
N
S
O2
OCD3
OMe
d3-3
ppm
2.08
1.00
0.91
1.05
2.14
1.06
1.87
3.22

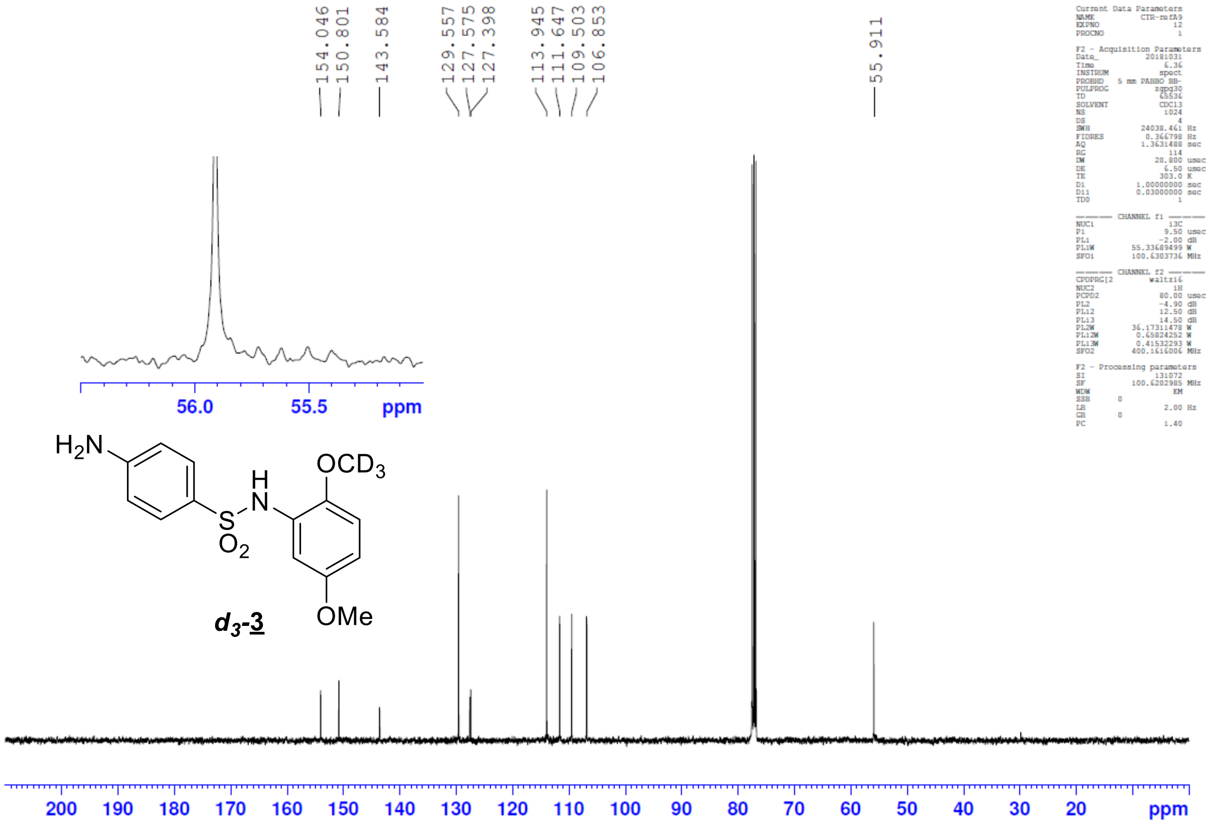
154.046
150.801
143.584
129.557
127.575
127.398
113.945
111.647
109.503
106.853
55.911
56.0
55.5
ppm
H2N
H
N
S
O2
OCD3
OMe
d3-3

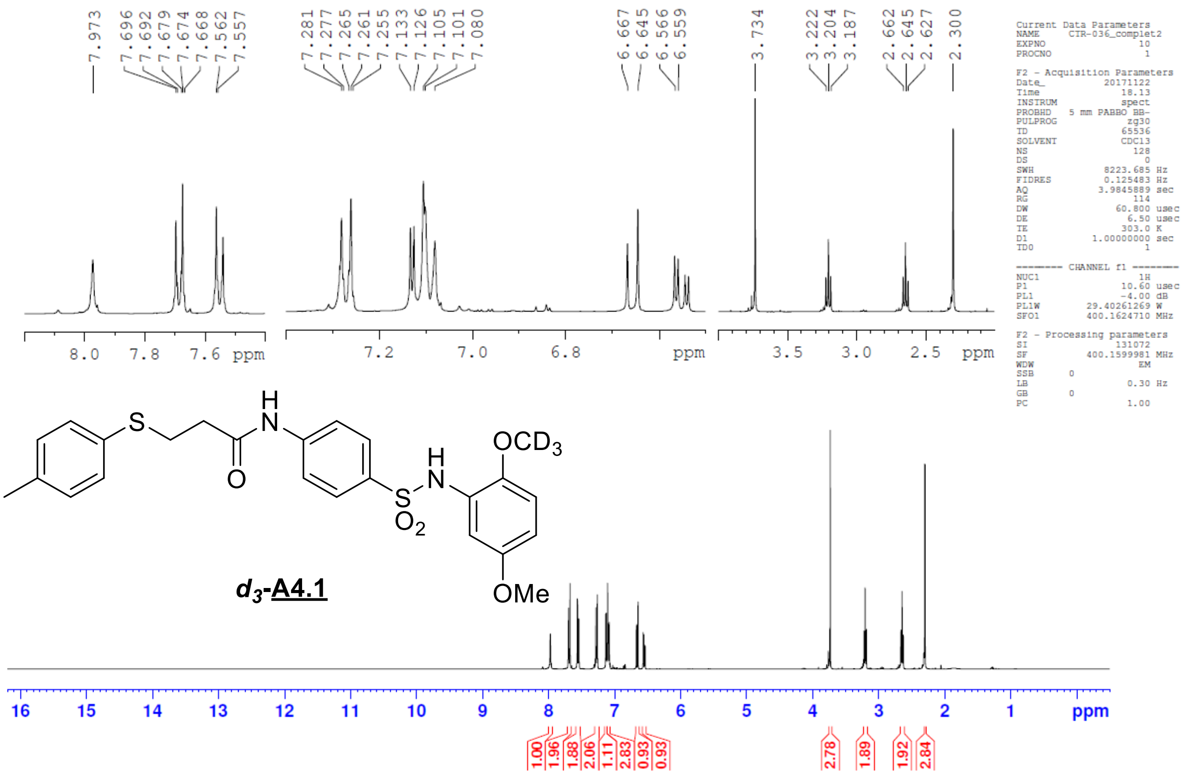
7.973
7.696 7.692 7.679 7.674 7.668 7.562 7.557
7.281 7.277 7.265 7.261 7.255 7.133 7.126 7.105 7.101 7.080
6.667 6.645 6.566 6.559
3.734
3.222 3.204 3.187
2.662 2.645 2.627
2.300
OCD3
OMe
d3-A4.1
1.00 1.96 1.88 2.06 1.11 2.83 0.93 0.93
2.78 1.89 1.92 2.84
ppm

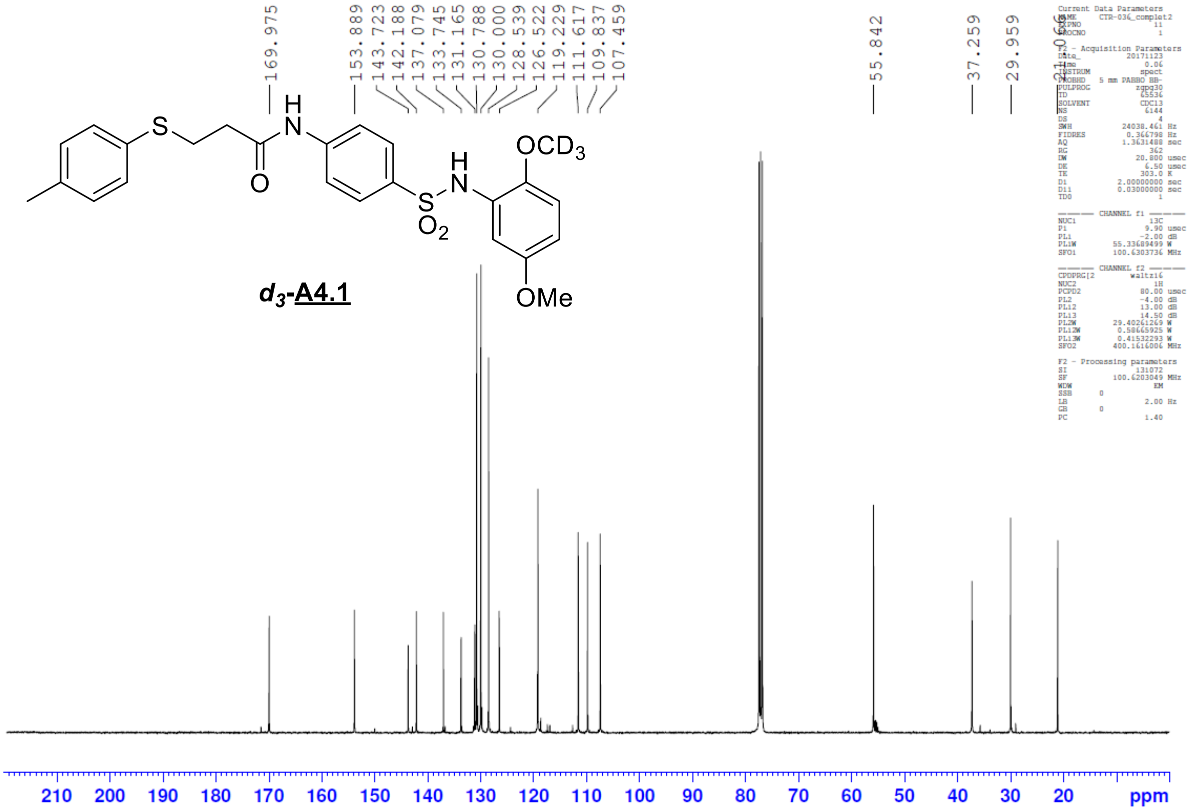
169.975
153.889 143.723 142.188 137.079 133.745 131.165 130.788 130.000 128.539 126.522 119.229 111.617 109.837 107.459
55.842
37.259
29.959
21.066
OCD3
OMe
d3-A4.1
ppm

HRMS spectra of the final compounds ***A4.1, A4.14, A4.15, A4.16, A4.20, d_3-A4.1***

Elemental Composition Report

Page 1

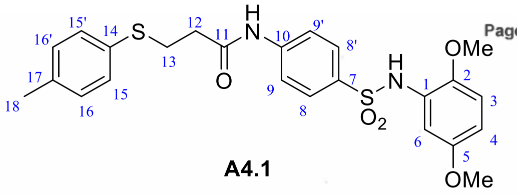

A4.1

Single Mass Analysis
Tolerance = 5.0 PPM / DBE: min = -1.5, max = 100.0
Element prediction: Off
Number of isotope peaks used for i-FIT = 4

Monoisotopic Mass, Even Electron Ions
135 formula(e) evaluated with 1 results within limits (up to 50 closest results for each mass)
Elements Used:
C: 0-30 H: 0-120 N: 0-2 O: 0-5 Na: 0-1 S: 2-2
LCT
MJR-59p2c2 / CH2Cl2+MeOH / ESI+
1: TOF MS ES+

20150413_MJR-59p2c2 59 (1.186) AM (Cen,13, 80.00, Ar,4100.0,556.28,0.00,LS 10); Cm (47:63)
13-Apr-2015

2.94e+004

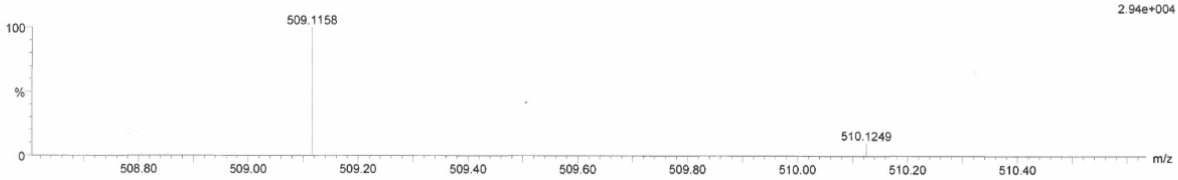

Mass	Calc. Mass	mDa	PPM	DBE	i-FIT	Formula
Minimum:				-1.5		
Maximum:		5.0	5.0	100.0		
509.1158	509.1181	-2.3	-4.5	12.5	n/a	C24 H26 N2 O5 Na S2

Elemental Composition Report

Page 1

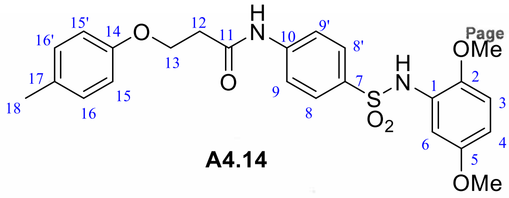

A4.14

Single Mass Analysis
Tolerance = 5.0 PPM / DBE: min = -1.5, max = 100.0
Element prediction: Off
Number of isotope peaks used for i-FIT = 4

Monoisotopic Mass, Even Electron Ions
105 formula(e) evaluated with 1 results within limits (up to 50 closest results for each mass)
Elements Used:
C: 0-100 H: 5-100 N: 0-2 O: 0-6 Na: 1-1 S: 1-1
LCT
MJR-77 / CH2Cl2+MeOH / ESI+
1: TOF MS ES+

20150602_MJR-77 13 (0.269) AM (Cen,13, 80.00, Ar,4000.0,556.28,0.00,LS 10); Cm (3:14)
02-Jun-2015

3.02e+004

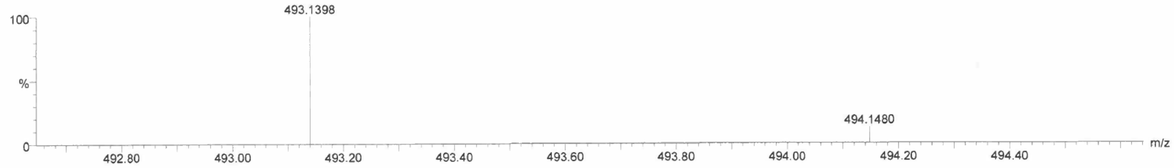

Mass	Calc. Mass	mDa	PPM	DBE	i-FIT	Formula
Minimum:				-1.5		
Maximum:		5.0	5.0	100.0		
493.1398	493.1409	-1.1	-2.2	12.5	n/a	C24 H26 N2 O6 Na S

Elemental Composition Report

Page 1

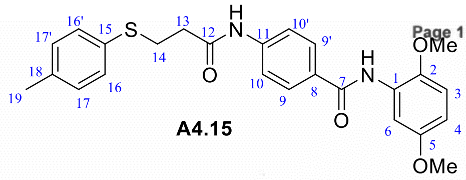

A4.15

Single Mass Analysis
Tolerance = 5.0 PPM / DBE: min = -1.5, max = 100.0
Element prediction: Off
Number of isotope peaks used for i-FIT = 4

Monoisotopic Mass, Even Electron Ions
622 formula(e) evaluated with 3 results within limits (up to 50 closest results for each mass)
Elements Used:
C: 0-100 H: 0-100 N: 0-3 O: 0-16 S: 0-1
LCT
MJR-80p2 / CH2Cl2+MeOH / ESI-
1: TOF MS ES-

20150528_MJR-80p2 3 (0.069) AM (Cen,13, 80.00, Ar,3000.0,554.26,0.00,LS 10); Cm (2:10)
28-May-2015

1.26e+004

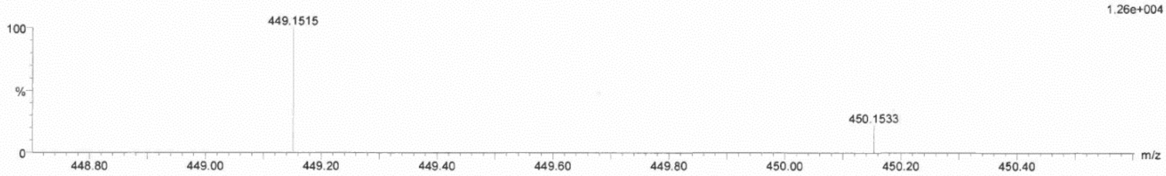

Mass	Calc. Mass	mDa	PPM	DBE	i-FIT	Formula
Minimum:				-1.5		
Maximum:		5.0	5.0	100.0		
449.1515	449.1506	0.9	2.0	1.5	n/a	C15 H29 O15
	449.1501	1.4	3.1	19.5	n/a	C28 H21 N2 O4
	449.1535	-2.0	-4.5	14.5	n/a	C25 H25 N2 O4 S

Elemental Composition Report A4.16

Page 1

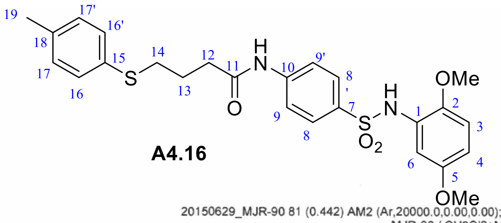

A4.16

Single Mass Analysis
Tolerance = 5.0 PPM / DBE: min = -1.5, max = 100.0
Element prediction: Off
Number of isotope peaks used for i-FIT = 4

Monoisotopic Mass, Even Electron Ions
700 formula(e) evaluated with 5 results within limits (up to 50 closest results for each mass)
Elements Used:
C: 0-100 H: 5-100 N: 0-6 O: 0-10 Na: 1-1 S: 1-2
SYNAPT-G2#NotSet
29-Jun-2015
1: TOF MS ES+

20150629_MJR-90 81 (0.442) AM2 (Ar,20000.0,0.00,0.00); Cm (69:96)
MJR-90 / CH2Cl2+MeOH / ESI+
9.81e+006

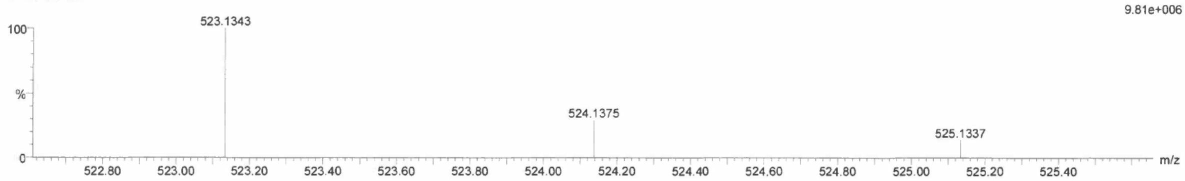

Minimum:				-1.5		
Maximum:		5.0	5.0	100.0		
Mass	Calc. Mass	mDa	PPM	DBE	i-FIT	Formula
523.1343	523.1344	-0.1	-0.2	21.5	n/a	C33 H24 O3 Na S
	523.1337	0.6	1.1	12.5	n/a	C25 H28 N2 O5 Na S2
	523.1351	-0.8	-1.5	17.5	n/a	C26 H24 N6 O Na S2
	523.1362	-1.9	-3.6	8.5	n/a	C21 H28 N2 O10 Na S
	523.1317	2.6	5.0	22.5	n/a	C29 H20 N6 O Na S

Elemental Composition Report A4.20

Page 1

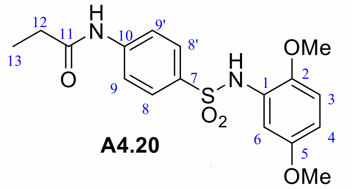

A4.20

Single Mass Analysis
Tolerance = 5.0 PPM / DBE: min = -1.5, max = 50.0
Element prediction: Off
Number of isotope peaks used for i-FIT = 4

Monoisotopic Mass, Even Electron Ions
3616 formula(e) evaluated with 20 results within limits (up to 50 closest results for each mass)
Elements Used:
C: 0-100 H: 0-100 N: 0-20 O: 0-20 Na: 0-1 S: 0-1
MJR101 / DCM - eau/ACN/AF (90/10/0.1%) / ESI+ XEVO G2-XS QTOF
20170116_MJR101 18 (0.381) AM2 (Ar,22000.0,0.00,0.00); Cm (17:20)

16-Jan-2017
1: TOF MS ES+
5.68e+007

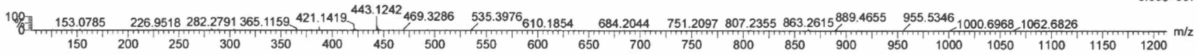

Minimum:				-1.5				
Maximum:		30.0	5.0	50.0				
Mass	Calc. Mass	mDa	PPM	DBE	i-FIT	Norm	Conf(%)	Formula
387.0977	387.0975	0.2	0.5	0.5	990.7	16.659	0.00	C8 H20 N4 O12 Na
	387.0975	0.2	0.5	11.5	989.3	15.255	0.00	C6 H8 N18 O2 Na
	387.0974	0.3	0.8	7.5	975.3	1.223	29.44	C14 H19 N4 O7 S
	387.0981	-0.4	-1.0	16.5	989.7	15.605	0.00	C22 H15 N2 O5
	387.0973	0.4	1.0	4.5	990.8	16.699	0.00	C6 H15 N10 O10
	387.0970	0.7	1.8	18.5	990.4	16.273	0.00	C21 H12 N6 O Na
	387.0986	-0.9	-2.3	9.5	990.0	15.951	0.00	C7 H11 N14 O6
	387.0986	-0.9	-2.3	-1.5	990.8	16.693	0.00	C9 H23 O16
	387.0988	-1.1	-2.8	12.5	976.3	2.205	11.02	C15 H15 N8 O3 S
	387.0989	-1.2	-3.1	5.5	990.2	16.116	0.00	C9 H16 N8 O8 Na
	387.0964	1.3	3.4	9.5	977.3	3.256	3.86	C13 H16 N8 O3 Na S
	387.0991	-1.4	-3.6	8.5	974.7	0.620	53.79	C17 H20 N2 O5 Na S
	387.0962	1.5	3.9	6.5	990.9	16.862	0.00	C5 H12 N14 O6 Na
	387.0961	1.6	4.1	13.5	978.8	4.716	0.90	C11 H11 N14 O S

..emental Composition Report

Page 1

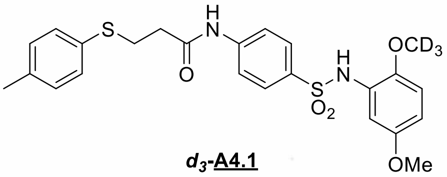

*d*3-**A4.1**

Single Mass Analysis
Tolerance = 5.0 PPM / DBE: min = -50.0, max = 500.0
Element prediction: Off
Number of isotope peaks used for i-FIT = 4

Monoisotopic Mass, Even Electron Ions
1116 formula(e) evaluated with 6 results within limits (all results (up to 1000) for each mass)
Elements Used:
C: 0-100 1H: 0-30 2H: 0-5 N: 0-5 O: 0-5 S: 2-2
CTR-036-F1 (DCM) - MeOH (100%) XEVO G2-XS QTOF
20171114_CTR-036-F1 17 (0.364) AM2 (Ar,22000.0,0.00,0.00); ABS; Cm (17:18)

14-Nov-2017
1: TOF MS ES+
3.31e+007

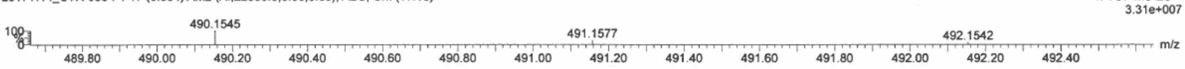

Minimum:				-50.0				
Maximum:		30.0	5.0	500.0				
Mass	Calc. Mass	mDa	PPM	DBE	i-FIT	Norm	Conf(%)	Formula
490.1545	490.1550	-0.5	-1.0	12.5	60.8	0.763	46.64	C24 1H24 2H3 N2 O5 S2
	490.1552	-0.7	-1.4	12.5	61.4	1.385	25.02	C22 1H24 2H2 N5 O4 S2
	490.1559	-1.4	-2.9	17.5	62.1	2.102	12.23	C29 1H20 2H5 O3 S2
	490.1561	-1.6	-3.3	17.5	62.1	2.066	12.66	C27 1H20 2H4 N3 O2 S2
	490.1522	2.3	4.7	20.5	63.7	3.728	2.40	C31 1H24 2H N2 S2
	490.1521	2.4	4.9	13.5	64.6	4.564	1.04	C22 1H20 2H4 N5 O4 S2

LCMS analysis of the final compound ***d_3-A4.1*** *for pharmacokinetic studies*

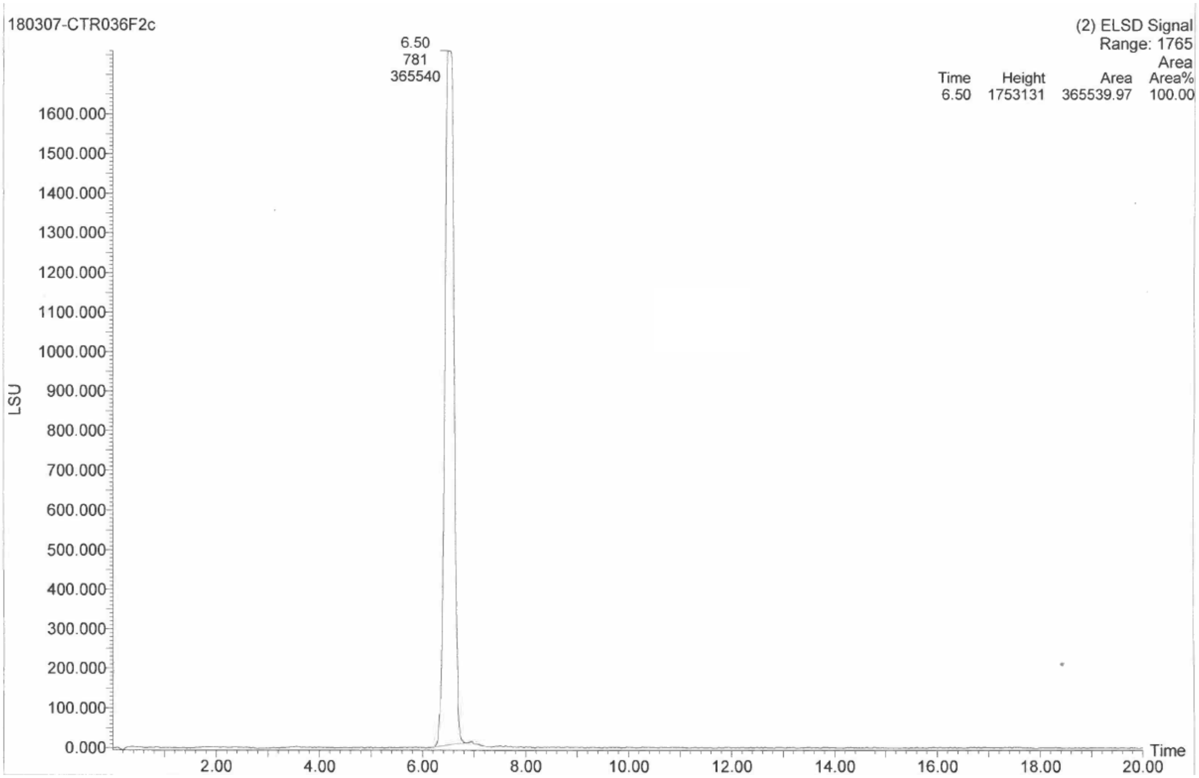

Data S8. Synthesis of inhibitors A41, A414, A416

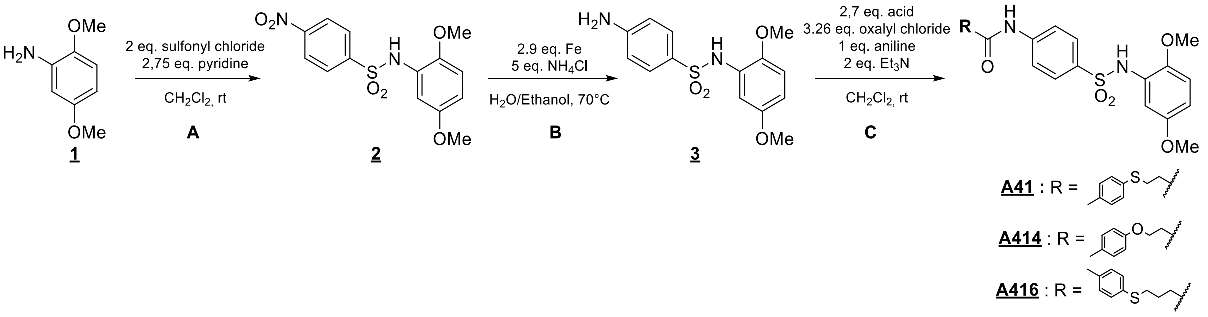

Synthesis of A41:

2 : *N*-(2,5-dimethoxyphenyl)-4-nitrobenzenesulfonamide :

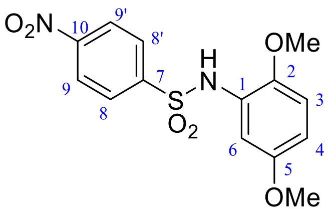

General procedure A :

To a suspension of commercial 4-nitrobenzenesulfonyl chloride (3g, 13.54 mmol) in CH_2Cl_2 (35 mL) were added dropwise commercial 2,5-dimethoxyaniline **1** (1.037g, 6.77 mmol) and pyridine (1.53 mL, 18.62 mmol) in CH_2Cl_2 (20 mL) at rt. After 3 hours of stirring at rt, the reaction mixture was quenched with water. The aqueous layer was extracted three times with CH_2Cl_2. The combined organic layers were washed with a saturated aqueous $NaHCO_3$ solution and brine, dried over $MgSO_4$, filtered and concentrated *in vacuo*. The crude was purified by column chromatography over silica gel (PE/AcOEt : 7/3) affording the expected compound **2** as a yellow solid (4.15g, 90%).

R_f : 0.42 (PE/ AcOEt : 7/3) **m.p.** : 160°C **^{1}H NMR** : (400 MHz, $CDCl_3$, 25 °C) δ : 8.23 (m, 2H, H_9 et $H_{9'}$), 7.93 (m, 2H, H_8 et $H_{8'}$), 7.16 (d, ^4J = 2.86 Hz, 1H, H_6), 7.10 (s, 1H, NH), 6.66 (d, ^3J = 8.97 Hz, 1H, H_3), 6.60 (dd, ^4J = 2.86 Hz, ^3J = 8.97 Hz, 1H, H_4), 3.76 (s, 1H, $O\underline{CH_{3(5)}}$), 3.59 (s, 1H, $O\underline{CH_{3(2)}}$), **^{13}C NMR** : (100 MHz, $CDCl_3$, 25 °C) δ : 154.1 (C_5), 150.3 (C_{10}), 144.9 (C_7), 144.0 (C_2), 128.6 (C_8 et $C_{8'}$), 125.5 (C_1), 124.1 (C_9 et $C_{9'}$), 111.7 (C_3), 110.9 (C_4), 108.4 (C_6), 56.2 ($O\underline{C}H_{3(2)}$), 55.9 ($O\underline{C}H_{3(5)}$) **HRMS** : (ES-) m/z calculated for $C_{14}H_{13}N_2O_6$ $[M-H]^-$, 337.0494; found 337.0505 **FT-IR** : $\nu(NH_{sulfonamide})$: 3309, $\nu(CH_{ar})$: 3107, $\nu(CH_{Me})$: 2945, 2839, $\nu_{as}(NO_2)$:1533, $\nu_s(NO_2)$: 1346, $\nu_s(SO)$: 1173, $\nu(CN)$: 858.

3 : 4-amino-N-(2,5-dimethoxyphenyl)benzenesulfonamide :

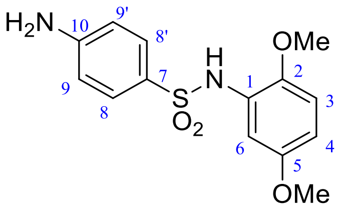

General procedure B :

To a solution of *N*-(2,5-dimethoxyphenyl)-4-nitrobenzenesulfonamide **2** (2 g, 6.48 mmol) in EtOH (100 mL) were successively added iron (1.06 g, 19 mmol) and an aqueous solution of NH_4Cl (1.72 g, 32.46 mmol in 20 mL of water). After stirring over 6 hours at 70 °C, the reaction mixture was filtered through a pad of celite on sintered funnel. After successive washings with acetone, CH_2Cl_2 and AcOEt, the biphasic mixture was separated. The aqueous layer was extracted twice with CH_2Cl_2. The combined organic layers were dried over $MgSO_4$ and the solvents were concentrated under vacuum. The crude was purified by chromatography over silica gel (PE/AcOEt : 1/1) affording the expected compound **3** as a light brown solid (1.37 g, 68%).

R*f* : 0.22 (PE/ AcOEt : 1/1) **m.p.** : 115°C **^{1}H NMR** : (300 MHz, *d6*-DMSO, 25 °C) δ : 8.81 (s, 1H, NH), 7.38 (m, 2H, H_9-$H_{9'}$), 6.82 (d, 1H, H_3, ^3J = 8.9 Hz), 6.78 (d; 1H, H_6, ^4J = 2.9 Hz), 6.56 (dd, 1H, H_4, ^3J = 8.8 Hz, ^4J = 2.9 Hz), 6.52 (m, 2H, H_8-$H_{8'}$), 5.96 (s, 2H, N$\underline{H_2}$), 3.63 (s, 3H, OC$\underline{H}_{3(5)}$), 3.56 (s, 3H, OC$\underline{H}_{3(2)}$) **^{13}C NMR** : (75 MHz, *d6*-DMSO, 25 °C) δ : 152.8 (C_5 and C_{10}), 144.9 (C_2), 128.7 (C_8-$C_{8'}$), 127.3 (C_7), 124.8 (C_1), 112.5 (C_3), 112.3 (C_9-$C_{9'}$), 108.7 (C_4), 108.5 (C_6), 56.2 (O$\underline{C}H_{3(5)}$), 55.2 (O$\underline{C}H_{3(2)}$) **HRMS :** (ES+) m/z calculated for $C_{14}H_{16}N_2O_4SNa$ $[M-Na]^+$, 331.0728; found 331.0727 **FT-IR :** ν(NH_{amine}) : 3461, 3366 ; ν($NH_{sulfonamide}$): 3218 ; ν(CH_{ar}) : 3110 ; ν(CH_{Me}) : 2939, 2839 ; $ν_{as}$(SO) : 1304 ; ν (CN) : 1257; $ν_s$(SO): 1144.

A41: N-(4-(N-(2,5-dimethoxyphenyl)sulfamoyl)phenyl)-3-(p-tolylthio)propanamide

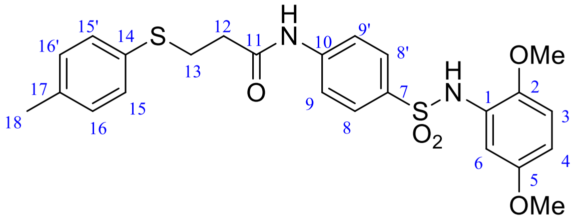

General procedure C:

In a 50 mL flask, commercial 3-(*p*-tolylthio)propanoic acid (0.38 g, 1.95 mmol) was dissolved in dry CH_2Cl_2 (10 mL) under argon atmosphere. Oxalyl chloride (0.17 mL, 1.95 mmol) and DMF (0.03 mL) were successively added to the reaction mixture at 0°C. After 15 minutes of stirring, bubbling stopped and an NMR analysis confirmed total conversion to the acyl chloride. Oxalyl chloride and CH_2Cl_2 were

removed under reduced pressure. To a solution of the resulting 3-(*p*-tolylthio)propanoyl chloride in dry CH_2Cl_2 (10 mL) were added dropwise at 0°C 4-amino-*N*-(2,5-dimethoxyphenyl)benzenesulfonamide **3** (0.6 g, 1.95 mmol) and Et_3N (0.15 mL, 1.95 mmol) dissolved in dry CH_2Cl_2 (10 mL). After completion, the reaction mixture was quenched with an aqueous 5% $NaHCO_3$ solution. The aqueous layer was extracted three times with CH_2Cl_2. The combined organic layers were washed successively with a molar solution of HCl and brine, dried over $MgSO_4$, filtered and concentrated *in vacuo*. The crude was purified by chromatography over silica gel (PE/AcOEt/CH_2Cl_2 : 5/3/2) then affording the expected compound **A41** as a white solid (0.745 g, 78%).

R_f : 0.15 (PE/ AcOEt/ CH_2Cl_2 : 5/3/2) **m.p.** : 129°C **^{1}H NMR** : (300 MHz, $CDCl_3$, 25 °C) δ ppm: 7.70 (m, 2H, H_8-$H_{8'}$), 7.60 (s, 1H, CON<u>H</u>), 7.53 (m, 2H, H_9-$H_{9'}$), 7.29 (m, 2H, H_{15}-$H_{15'}$), 7,13 (d, 1H, H_6, ^4J = 2,8 Hz), 7.10 (m, 2H, H_{16}-$H_{16'}$), 7.03 (s, 1H, SO_2N<u>H</u>), 6,65 (d, 1H, H_3, ^3J = 8,8 Hz), 6.53 (dd, 1H, H_4, ^3J = 8,9 Hz,^4J = 2,9 Hz), 3.74 (s, 3H, $OC\underline{H}_{3(5)}$), 3.61 (s, 3H, $OC\underline{H}_{3(2)}$), 3.21 (t, 2H, H_{12}, ^3J = 6,9 Hz), 2.62 (t, 2H, H_{13}, ^3J = 6,9 Hz), 2,30 (s, 3H, H_{18}) **^{13}C NMR** : (75 MHz, $CDCl_3$, 25 °C) δ ppm: 169.6 (C_{11}), 153.9 (C_2), 143.49 (C_{10}), 141.8 (C_5), 137.2 (C_{17}), 133.9 (C_7), 130.9 (C_{15}-$C_{15'}$), 130.7 (C_{14}), 130.0 ($C_{8-8'}$), 128.6 (C_{16}-$C_{16'}$), 126.5 (C_1), 119.1 (C_9-$C_{9'}$), 111.5 (C_3), 109.7 (C_4), 107.1 (C_6), 56.2 ($O\underline{C}H_{3(5)}$), 55.8 ($O\underline{C}H_{3(2)}$), 37.2 (C_{12}), 29.9 (C_{13}), 21.0 (C_{18}) **HRMS** : (ES+) m/z calculated for $C_{24}H_{27}N_2O_5S_2$ $[M-H]^+$, 487.1361 ; found 487.1362 **FT-IR :** ν(NH_{amide}) : 3310 ; ν($NH_{sulfonamide}$): 3242 ; ν(CH_{ar}) :3125, 3066 ; ν(CH_{OMe}) : 2953, 2833 ; ν(C=O) : 1689 ; $ν_{as}$(SO) : 1330; $ν_s$(SO): 1149.

<u>A414: N-(4-(N-(2,5-dimethoxyphenyl)sulfamoyl)phenyl)-3-(ptolyloxy) propanamide</u>

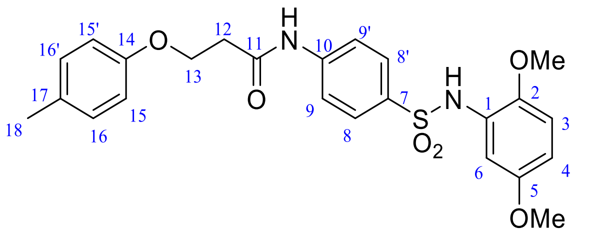

A414 was obtained following general procedure **C**, using commercial 3-(*p*-tolyloxy)propanoic acid (0.36 g, 2 mmol), oxalyl chloride (0.17 mL, 2 mmol), 4-amino-N-(2,5-dimethoxyphenyl)benzenesulfonamide **3** (0.308 g, 1 mmol) and Et_3N (0.21 mL, 1.5 mmol). After purification (PE/AcOEt : 1/1), it afford the expected compound **A4.14** as a white solid (0.26 g, 55%). **R_f** : 0.46 (CH_2Cl_2/AcOEt: 9/1) **m.p.** : 145°C **^{1}H NMR** : (300 MHz, $CDCl_3$, 25 °C) δ ppm: 8.15 (s, 1H, CON<u>H</u>), 7.71 (m, 2H, H_8-$H_{8'}$), 7.55 (m, 2H, H_9-$H_{9'}$), 7.14 (d, 1H, H_6, ^4J = 2.9 Hz), 7.10 (m, 2H, H_{15}-$H_{15'}$), 7.03 (s, 1H, SO_2N<u>H</u>), 6.83 (m, 2H, H_{16}-$H_{16'}$) 6.65 (d, 1H, H_3, ^3J = 8.9 Hz), 6.52 (dd, 1H, H_4, ^3J = 8.9 Hz,^4J = 2.9 Hz), 4.28 (t, 2H, H_{13}, ^3J = 5.7 Hz), 3.74 (s, 3H, $OC\underline{H}_{3(5)}$), 3.61 (s, 3H, $OC\underline{H}_{3(2)}$), 2.82 (t, 2H, H_{12}, ^3J = 5.7 Hz), 2.31 (s, 3H, H_{18}) **^{13}C NMR** : (75 MHz, $CDCl_3$,

25 °C) δ ppm: 169.5 (C_{11}), 155.8 (C_{14}), 154.1 (C_2), 143.7 (C_{10}), 142.1 (C_5), 134.1 (C_7), 131.4 (C_{17}), 130.3 (C_{16}-$C_{16'}$), 128.7 (C_8-$C_{8'}$), 126.7 (C_1), 119.3 (C_9-$C_{9'}$), 114.7 (C_{15}-$C_{15'}$), 111.7 (C_3), 110.0 (C_4), 107.3 (C_6), 64.3 (C_{13}), 56.4 (O<u>C</u>$H_{3(5)}$), 55.9 (O<u>C</u>$H_{3(2)}$), 37.9 (C_{12}), 20.6 (C_{18}) **HRMS** : (ES+) m/z calculated for $C_{24}H_{26}N_2O_6SNa$ $[M-Na]^+$, 493.1409; found 493.1410 **FT-IR :** ν(NH) : 3242 ; ν(CH_{ar}) : 3065 ; ν(CH_{OMe}) : 2837 ; $ν_{as}$(SO) : 1321 ; ν (CN): 1283 ; $ν_s$(SO): 1152.

<u>A416: N-(2,5-dimethoxyphenyl)-4-(3-(p-tolylthio)butanamido)benzamide</u>

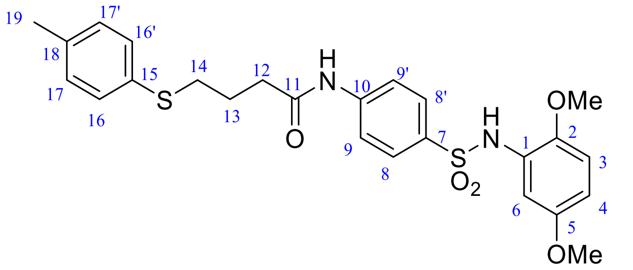

4-methyl-thiophenol (1.77 g, 14.3 mmol) was dissolved in DMF (18 mL). To the stirred solution at 0°C was added carefully in four times sodium hydride (0.57 g, 23.8 mmol, 60 %). The reaction mixture was kept stirring at 0°C for 30 min. γ-lactone was then added dropwise. After stirring at 0°C for 15 min, the reaction was warmed to rt, and finally to 90°C. After cooling down at rt, the reaction mixture was diluted with AcOEt and quenched with HCl (1M). The two layers were separated and the organic phase was washed with water and brine, dried over Na_2SO_4, filtered and concentrated off under vacuo. A purification of the crude (CH_2Cl_2/EtOAc : 9/1) was performed to obtain 4-(p-tolylthio)butanoic acid as a light yellow solid (0.9 g, 30%) **^{1}H NMR** (300 MHz, $CDCl_3$) : 7.27 (m, 2H, H_6-$H_{6'}$), 7.10 (m, 2H, H_7-$H_{7'}$), 2.93 (t, 2H, ^3J = 7.2 Hz, H_4), 2.51 (t, 2H, ^3J = 7.2 Hz, H_2), 2.32(s, 3H, H_9), 1.92 (quint, 2H, ^3J = 7.2 Hz, H_3) **^{13}C NMR** (75 MHz, $CDCl_3$) : 178.8 (C_1), 136.5 (C_8), 131.9 (C_5), 130.4 (C_7-$C_{7'}$), 129.8 (C_6-$C_{6'}$), 33.6 (C_2), 32.5 (C_4), 24.0 (C_3), 21.0 (C_9).

A416 was obtained following general procedure **C**, using the corresponding 4-(p-tolylthio)butanoyl chloride obtained from 4-(p-tolylthio)butanoic acid (0.42 g, 2 mmol), oxalyl chloride (0.17 mL, 2 mmol), 4-amino-N-(2,5dimethoxyphenyl)benzenesulfonamide **<u>3</u>** (0.2 g, 0.65 mmol) and Et_3N (0.11 mL, 0.78 mmol). After purification by precipitation in iPrOH, the expected compound **A416** is isolated as a white solid (0.1 g, 20%). **R_f** : 0.12 (CH_2Cl_2) **m.p.** : 153°C **^{1}H NMR (300 MHz, CDCl3) :** 7.70 (m, 2H, H8-H8'), 7.52 (m, 2H, H9-H9'), 7.40 (s, 1H, CON<u>H</u>), 7.24 (m, 2H, H_{16}-$H_{16'}$), 7.13 (d, 1H, H6, ^4J = 2.8 Hz), 7.07 (m, 2H, H_{17}-$H_{17'}$), 7.04 (s, 1H, SO_2N<u>H</u>), 6.64 (d, 1H, H3, ^3J = 8.9 Hz), 6.53 (dd, 1H, H_4, ^4J = 2.8 Hz, ^3J = 8.9 Hz), 3.74 (s, 3H, OC<u>$H_{3(5)}$</u>), 3.61 (s, 3H, OC<u>$H_{3(2)}$</u>), 2.96 (t, 2H, H14, ^3J = 6.8Hz), 2.51 (t, 2H, H12, ^3J = 7.2 Hz), 2.96 (s, 3H, H19), 2.01 (q, 2H, H7, ^3J = 7.0 Hz) **^{13}C NMR** (75 MHz, $CDCl_3$) : 170.9 (C_{11}), 154.0 (C_5), 143.7 (C_{10}), 142.1 (C_2), 136.7 (C_{18}), 133.9 (C_7), 131.9 (C_{15}), 130.5 (C_{16}-$C_{16'}$), 130.0 (C_{17}-$C_{17'}$), 128.7 (C_8-$C_{8'}$), 126.7 (C_1), 119.1 (C_9-$C_{9'}$), 111.6 (C_6), 109.9 (C_3), 107.2 (C_4), 56.4 ($OCH_{3(5)}$), 55.9 ($OCH_{3(2)}$), 35.8 (C_{12}), 33.8 (C_{14}), 24.5

(C_{13}), 21.1 (C_{19}) **HRMS** : (ES+) m/z calculated for $C_{26}H_{28}N_2O_4SNa$ $[M-Na]^+$, 523.1337; found 523.1340 **FT-IR :** ν(NH) : 3312 ; ν(CH_{ar}) : 2917 ; ν(CH_{OMe}) : 2832 ; $ν_{as}$(SO) : 1325 ; ν (CN) : 1304 ; $ν_s$(SO): 1157.

Synthesis of inhibitor A415

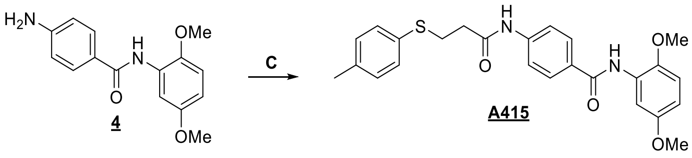

A415 : N-(2,5-dimethoxyphenyl)-4-(3-(p-tolylthio)propanamido)benzamide

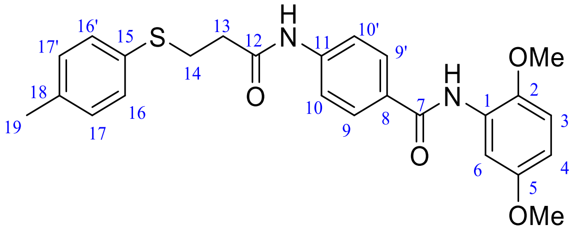

A415 was obtained following general procedure **C**, using commercial 3-(p-tolyloxy)propanoic acid (0.588 g, 3 mmol), oxalyl chloride (0.26 mL, 3 mmol), commercial 4-amino-N-(2,5-dimethoxyphenyl)benzamide **4** (0.5 g, 1.6 mmol) and Et3N (0.17 mL, 1.2 mmol). After purification (CH_2Cl_2/EtOAc: 100/0 to 9/1), it afford the expected compound **A415** as a white solid (0.05 g, 10%) **R*f*** : 0.35 (CH_2Cl_2/AcOEt: 9/1) **m.p.** : 153°C **^{1}H NMR** : (300 MHz, *d6*-DMSO, 25 °C) δ ppm: 10.25 (s, 1H, $C_{(12)}ON\underline{H}$), 9.22 (s, 1H, $C_{(7)}ON\underline{H}$), 7.91 (m, 2H, H_{10}-$H_{10'}$), 7.71 (m, 2H, H_9-$H_{9'}$), 7.56 (d, 1H, H_6, ^4J = 3.1 Hz), 7.29 (m, 2H, H_{16}-$H_{16'}$), 7.16 (m, 2H, H_{17}-$H_{17'}$), 7.01 (d, 1H, H_3, ^3J = 9.0 Hz), 6.72 (dd, 1H, H_4, ^4J = 3.1 Hz , ^3J = 9.0 Hz), 3.80 (s, 3H, $OC\underline{H}_{3(5)}$), 3.71 (s, 3H, $OC\underline{H}_{3(2)}$), 3.21(t, 2H, H_{14}, ^3J = 7.2 Hz), 2.67 (t, 2H, H_{13}, ^3J = 7.2 Hz), 2.27 (s, 3H, H_{19}) **^{13}C NMR** : (75 MHz, *d6*-DMSO, 25 °C) δ ppm: 169.7 (C_{12}), 164.2 (C_7), 152.9 (C_5), 144.9 (C_{11}), 142.2 (C_2), 135.6 (C_{18}), 131.9 (C_{15}), 129.8 (C_{17}-$C_{17'}$), 129.1 (C_{16}-$C_{16'}$), 128.6 (C_8), 128.4 (C_{10}-$C_{10'}$), 127.8 (C_1), 118.4 (C_9-$C_{9'}$), 111.9 (C_4), 109.7 (C_6), 109.2 (C_3), 56.3 ($O\underline{C}H_{3(5)}$), 55.4 ($O\underline{C}H_{3(2)}$), 36.3(C_{13}), 28.5 (C_{14}), 20.5 (C_{19}) **HRMS** : (ES+) m/z calculated for $C_{25}H_{26}N_2O_4SNa$ $[M-Na]^+$, 473.1511; found 473.1510 **FT-IR :** ν(NH) : 3316 ; ν(CH_{ar}) : 3005 ; ν(CH_{OMe}) : 2838 ; $ν_{as}$(SO) : 1366 ; ν (CN) : 1302 ; $ν_s$(SO): 1163.

[N_3]-A41:3-((4-azidophenyl)thio)-N-(4-(N-(2,5-dimethoxyphenyl)sulfamoyl)phenyl)propanamide

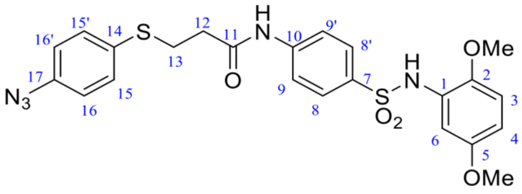

[N_3]-A41 was obtained following general procedure **C**, using 3-(p-azido-thiophenoxy)propanoic acid (0.179 g, 0.8 mmol), oxalyl chloride (0.07 mL, 0.8 mmol), commercial 4-amino-N-(2,5-dimethoxyphenyl)benzamide **4** (0.247 g, 0.8 mmol) and pyridine (1.7 mL). After purification (CH_2Cl_2/EtOAc: 100/0 to 9/1), it afford the expected compound **[N_3]-A41** as a pale yellow solid (0.3 g, 73%) **R_f** 0.23 (4% EtOAc/ DCM) ; **m.p.** 136-137°C ; **^{1}H NMR** : (300 MHz, $CDCl_3$) : δ 7.68 (m, 2H, H^{Ar}), 7.63 (brs, 1H, NH), 7.52 (m, 2H, H^{Ar}), 7.34 (m, 2H, H^{Ar}), 7.10 (d, J_4 = 3.0 Hz, 1H, H^6), 7.04 (brs, 1H, NH), 6.93 (m, 2H, H^{Ar}), 6.64 (d, J_3 = 8.9 Hz, 1H, H^3), 6.53 (dd, J_4 = 3.0 Hz, J_3 = 8.9 Hz, 1H, H^4), 3.72 (s, 3H, OMe), 3.61 (s, 3H, OMe), 3.21 (t, J_3 = 7.3 Hz, 2H, H^{13}), 2.63 (t, J_3 = 6.8 Hz, 2H, H^{12}) ; **^{13}C NMR** : (75 MHz, $CDCl_3$) : δ 169.5 C^{11}, 154.0 C^2, 143.6 C^5, 142.0 C^{Ar}, 139.2 C^{Ar}, 134.0 C^{Ar}, 132.3 C^{Ar}, 128.7 C^{Ar}, 126.6 C^1, 119.9 C^{Ar}, 119.2 C^{Ar}, 111.6 C^3, 109.8 C^4, 107.3 C^6, 56.3 C^{OMe}, 55.9 C^{OMe}, 37.3 C^{12}, 30.1 C^{13} ; **HRMS** (ES-) m/Z calc for $C_{23}H_{22}N_5O_5S_2$ $[M-H]^-$ 512.1062; found 512.1055. **FT-IR :** 3245.0, 2087.63, 1674.2, 1505.9, 1218.5, 826.7.

Synthesis of inhibitor d_3-A41

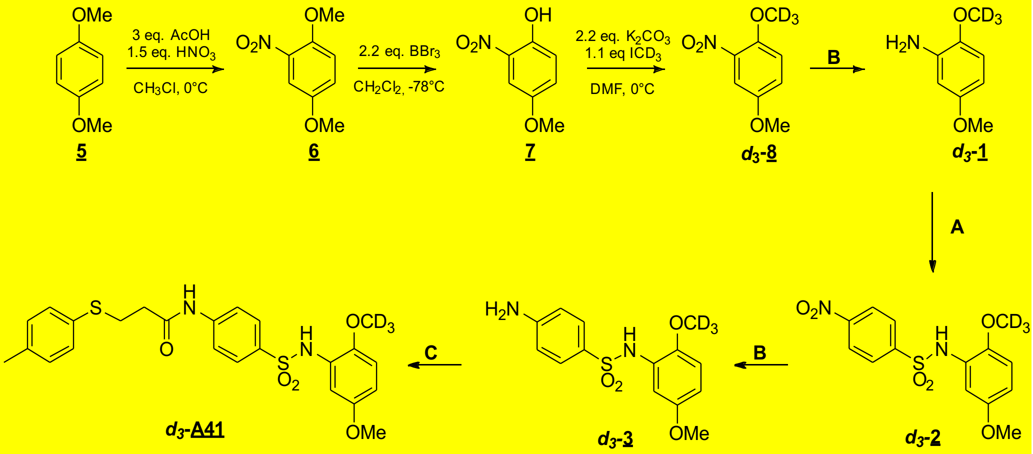

6 : 1,4-dimethoxy-2-nitrobenzene

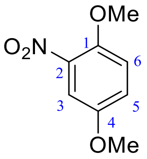

To commercial 1,4-dimethoxybenzene **5** (2 g, 14.47 mmol) solution in $CHCl_3$ (37 mL) and AcOH (2.48 mL, 43.41 mmol) cooled in an ice-bath, was added nitric acid (1.37mL, 21.7 mmol, 70% in water) dropwise. The reaction was stirred for 15 min and then poured onto an ice/water mixture (100 mL). The organic layer was washed with an aqueous $NaHCO_3$ solution (0.5N, 2 x 20 mL), water, and brine, dried over $MgSO_4$, filtered, concentrate *in vacuo*. The crude product was purified by column chromatography on silica gel (PE/ AcOEt 9:1) affording the expected product **6** as a yellow solid (2.15g, 81% yield) **R_f** : 0.30 (EP/AcOEt) **m.p.** : 72°C **^{1}H NMR** : (400 MHz, $CDCl_3$, 25 °C) δ : 7.39 (d, ^4J = 3.04 Hz, 1H, H_3), 7.12 (dd, ^3J = 9.18 Hz, ^4J = 3.04 Hz, 1H, H_5), 7.03 (d, ^3J = 9.18 Hz, 1H, H_6), 3.92 (s, 3H, $OC\underline{H}_{3(1)}$), 3.81 (s, 3H, $OC\underline{H}_{3(4)}$ **^{13}C NMR** : (100 MHz, $CDCl_3$, 25 °C) δ : 153.1 (C_4), 147.5 (C_1), 139.8 (C_2), 121.0 (C_5), 115.4 (C_6), 110.2 (C_3), 57.3 ($O\underline{C}H_{3(1)}$), 56.2 ($O\underline{C}H_{3(4)}$) **HRMS** : (ES+) m/z calculated for $C_8H_{10}NO_4$ $[M+H]^+$, 184.0610; found 184.0601 **FT-IR** : $\nu(CH_{Me})$: 2950, 2848, $\nu_{as}(NO_2)$:1522, $\nu_s(NO_2)$: 1351, $\nu(CN_{NO2})$: 873.

7 : 4-methoxy-2-nitrophenol

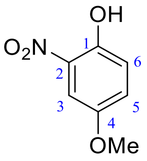

To a solution of 1,4-dimethoxy-2-nitrobenzene **6** (2.15g, 11.7 mmol) in CH_2Cl_2 (600mL) was added BBr_3 (25.8 mL, 1M in CH_2Cl_2, 25.8 mmol) at -78°C under inert atmosphere. The mixture was then stirred for 30 min and poured onto ice and aqueous saturated sodium bicarbonate solution. After being shaken vigorously, the mixture was extracted with CH_2Cl_2. The combined organic layers were then washed with water, brine, dried over $MgSO_4$, filtered and concentrated off. The crude product was purified by column chromatography on silica gel (PE/ CH_2Cl_2: 9/1) affording expected product **7** as an orange solid (1.85g, 94% yield).

R_f : 0.55 (PE/ AcOEt : 9/1+1% acetic acid) **m.p.:** 81 °C **^{1}H NMR** : (400 MHz, $CDCl_3$, 25 °C) δ : 10.33 (s, 1H, OH), 7.51 (d, ^4J = 3.12 Hz, 1H, H_3), 7.22 (dd, ^3J = 9.24 Hz, ^4J = 3 Hz, 1H, H_5), 7.09 (d, ^3J = 9.24 Hz, 1H, H_6), 3.83 (s, 3H, OMe) **^{13}C NMR** : (100 MHz, $CDCl_3$, 25 °C) δ : 152.8 (C_4), 150.2 (C_1), 133.2 (C_2), 127.4 (C_5), 121.0 (C_6), 105.9 (C_3), 56.2 (OMe) **HRMS :** (ES-) m/z calculated for $C_7H_6NO_4$ $[M-H]^-$, 168.0297; found 168.0289 **FT-IR :** ν(OH) : 3222 ; ν(CH_{ar}) : 3052 ; ν(CH_{Me}) : 2844 ; $ν_{as}(NO_2)$: 1527 ; $ν_s(NO_2)$: 1305 ; ν(CN) : 862, 761.

***d*₃-8 : 4-methoxy-1-(methoxy-*d3*)-2-nitrobenzene**

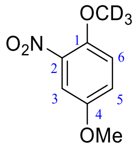

To a solution of 4-methoxy-2-nitrophenol **7** (860mg, 5.08 mmol) in DMF (7mL) was added K_2CO_3 (1.55g, 11.19 mmol) portionwise. After the solution became red, D_3-iodomethane (345 µL, 5.59 mmol) was added and was stirred overnight at rt and filtered. The filtrate was diluted with AcOEt (25mL) and washed with an aqueous saturated sodium bicarbonate solution (5 x 30 mL). The organic layer was dried over $MgSO_4$, filtered and concentrate. The expected product ***d*₃-8** was obtained without further purification needed as a yellow solid (815 mg, 86% yield). **R_f** : 0.32 in petroleum ether/ Ethyl acetate 9:1 **m.p.**: 74 °C **^{1}H NMR :** (400 MHz, $CDCl_3$, 25 °C) δ : 7.39 (d, ^4J = 3.10 Hz, 1H, H_3), 7.11 (dd, ^3J = 9.04 Hz, ^4J = 3.10 Hz, 1H, H_5), 7.02 (d, ^3J = 9.04 Hz, 1H, H_6), 3.81 (s, 3H, OMe) **^{13}C NMR :** (100 MHz, $CDCl_3$, 25 °C) δ : 153.0 (C_4), 147.5 (C_1), 139.8 (C_2), 121.0 (C_5), 115.3 (C_6), 110.2 (C_3), 56.4 (OCD_3), 56.2 (OMe) **HRMS** : (ES+) m/z calculated for $C_8H_7D_3NO_4$ $[M+H]^+$, 187.0798; found 187.0796 **FT-IR :** ν(CH_{Me}) : 2851 ; ν(CD_{CD3}) : 2077, $ν_{as}(NO_2)$: 1521 ; $ν_s(NO_2)$: 1351 ; ν(CN) : 873.

***d*₃-1 : 5-methoxy-2-(methoxy-*d3*)aniline**

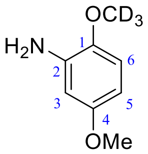

***d*$_3$-1** was obtained following general procedure **B**, using 4-methoxy-1-(methoxy-*d3*)-2-nitrobenzene ***d*$_3$-8** (0.6g, 3.22 mmol), iron (1.07g, 19.32 mmol) and NH_4Cl (1.7g in 15 mL of water, 32.2 mmol). No further purification needed to afford the expected compound ***d*$_3$-1** as a green solid (0.48 g, 96%). **R_f** : 0.42 (PE/AcOEt : 9/1) **m.p.**: 81 °C **^{1}H NMR** : (400 MHz, $CDCl_3$, 25 °C) δ : 6.69 (d, ^3J = 8.71 Hz, 1H, H_3), 6.33 (d, ^4J = 2.86 Hz, 1H, H_6), 6.24 (dd, ^3J = 8.71 Hz, ^4J = 2.86 Hz, 1H, H_4), 3.73 (s, 3H, OMe_5). **^{13}C NMR** : (100 MHz, $CDCl_3$, 25 °C) δ : 154.6 (C_5), 142.0 (C_2), 137.4 (C_1), 111.5 (C_3), 102.3 (C_6), 102.1 (C_4), 55.7 (OMe_5), 55.7 (m, ^1J ≈ 22 Hz , OCD_3). **HRMS** : (ES+) m/z calculated for $C_8H_9D_3NO_2$ $[M+H]^+$, 157.1056; found 157.1053 **FT-IR :** ν(NH) : 3457, 3366 ; ν(CD_{CD3}) : 2071 ; ν(CN) : 1227.

<u>*d*$_3$-2 :N-(5-methoxy-2-(methoxy-*d3*)phenyl)-4-nitrobenzenesulfonamide</u>

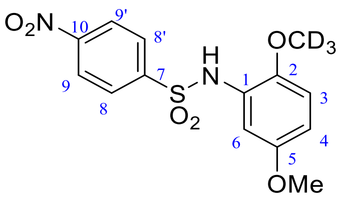

***d*$_3$-2** was obtained following general procedure A, using 5-methoxy-2-(methoxy-d3)aniline ***d*$_3$-1** (483 mg, 3,09 mmol), pyridine (432 µL, 5.37 mmol) and 4-nitrobenzenesulfonile chloride (0.85g, 3.84 mmol). After purification (PE/EtOAc: 7/3), it afford the expected compound ***d*$_3$-2** as a yellow solid (0.95 g, 90%). **R_f** : 0.42 (PE/AcOEt : 7/3) **m.p.**: 160 °C **^{1}H NMR** : (300 MHz, $CDCl_3$, 25 °C) δ : 8.25-8.21 (m, ^3J = 8.83 Hz, ^4J = 2.38 Hz, ^5J = 1.96 Hz, 2H, H_9 et $H_{9'}$), 7.96-7.91 (m, ^3J = 8.83 Hz, ^4J = 2.38 Hz, ^5J = 1.96 Hz, 2H, H_8 et $H_{8'}$), 7.16 (d, ^4J = 2.80 Hz, 1H, H_6), 7.12 (s, 1H, NH), 6.66 (d, ^3J = 8.97 Hz, 1H, H_3), 6.59 (dd, ^3J = 8.97 Hz, ^4J = 2.80 Hz 1H, H_4), 3.75 (s, 3H, OMe) **^{13}C NMR** : (75 MHz, $CDCl_3$, 25 °C) δ : 154.0, 150.3, 144.9, 144.0 (C^{IV}), 128.6 (2 C^{IV}), 125.4 (C^{IV}), 124.1 (2C^{IV}), 111.6 (C_3), 110.8 (C_4), 108.4 (C_6), 55.9 (OCH_3), 55.6 (m, J = 22.18 Hz,OCD_3) **HRMS :** (ES-) m/z calculated for $C_{14}H_{10}D_3N_2O_6S$ $[M-H]^-$, 340.0683; found 340.0683 **FT-IR** : ν($NH_{sulfonamide}$) : 3311, ν(CH_{ar}) : 3107, ν(CD_{CD3}) : 2079, $\nu_{as}(NO_2)$:1533, $\nu_s(NO_2)$: 1346, $\nu_s(SO)$: 1173, ν(CN) : 855.

***d*$_3$-3 : 4-amino-N-(5-methoxy-2-(methoxy-d3)phenyl)benzenesulfonamide**

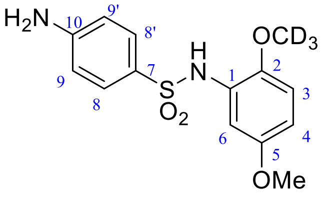

***d*$_3$-3** was obtained following general procedure B, using 4-methoxy-1-(methoxy-*d3*)-2-nitrobenzenesulfonamide ***d*$_3$-2** (1.08g, 3.16 mmol), iron (1.06g, 18.96 mmol) and NH_4Cl (1.67g in 15 mL of water, 31.6 mmol). No purification needed to afford the expected compound ***d*$_3$-3** as a beige solid

(910mg, 92%) **R_f** : 0.079 (PE/AcOEt : 7/3) **m.p.**: 120 °C **^{1}H NMR** : (300 MHz, $CDCl_3$, 25 °C) δ : 7.57-7.54 (m, 2H, H_9-$H_{9'}$), 7.12 (d; 1H, H_6, ^4J = 2.9 Hz), 6.98 (s, 1H, NH), 6.65 (d, 1H, H_3, ^3J = 8.9 Hz), 6.59-6.54 (m, 2H, H_8-$H_{8'}$), 6.51 (dd, 1H, H_4, ^3J = 8.8 Hz, ^4J = 2.9 Hz), 4.06 (s, 2H, NH_2), 3.73 (s, 3H, OMe_5) **^{13}C NMR** : (75 MHz, $CDCl_3$, 25 °C) δ : 154.0 (C_5) , 150.8 (C_{10}), 143.6 (C_2), 129.6 (C_8-$C_{8'}$), 127.6 (C_1), 127.4 (C_7), 113.9 (C_9-$C_{9'}$), 111.6 (C_3), 109.5 (C_4), 106.9 (C_6), 55.9 (OCH_3), 55.6 (m, *J* = 22.18 Hz,OCD_3) **HRMS** : (ES-) m/z calculated for $C_{14}H_{12}D_3N_2O_4S$ $[M-H]^-$, 310.0941; found 310.0939 **FT-IR :** ν(NH_{amine}) : 3483,3391 ; ν($NH_{sulfonamide}$): 3259 ; ν(CH_{Me}) : 2939 ; ν(CD_{CD3}) : 2073 ; ν_{as}(SO) : 1308 ; ν (CN) : 1217 ; ν_s(SO): 1152.

d_3-A41: N-(4-(N-(5-methoxy-2-(methoxy-d3)phenyl)sulfamoyl)phenyl)-3-(p-tolylthio) propanamide

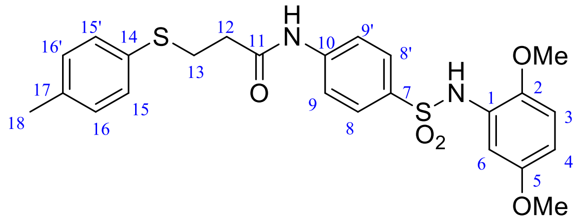

***d_3*-3** was obtained following general procedure C, using commercial 3-(*p*-tolylthio)propanoic acid (1.58 g, 2.75 mmol), oxalyl chloride (0.69 mL, 8.1 mmol), 4-amino-N-(5-methoxy-2-(methoxy-d3)phenyl)benzenesulfonamide ***d_3*-3** (0.5 g, 1.6 mmol) and Et_3N (0.24 mL, 1.76 mmol). After purification (PE/EtOAc/CH_2Cl_2 : 6/2/2), it afford the expected compound ***d_3*-A4.1** as a white solid (0.59 g, 69%) **R_f** : 0.15 (PE/ AcOEt/ CH_2Cl_2 : 5/3/2) **m.p.** : 129°C **^{1}H NMR** : (300 MHz, $CDCl_3$, 25 °C) δ ppm: 7.97 (s, 1H, CON<u>H</u>), 7.69-7.66 (m, 2H, H_8-$H_{8'}$), 7.56-7.55 (m, 2H, H_9-$H_{9'}$), 7.28-7.25 (m, 2H, H_{14}-$H_{14'}$), 7,13 (d, 1H, H_6, ^4J = 2,8 Hz), 7.11-7.08 (m, 3H, H_{15}-$H_{15'}$ and SO_2N<u>H</u>), 6,65 (d, 1H, H_3, ^3J = 8,8 Hz), 6.55 (dd, 1H, H_4, ^3J = 8,9 Hz,^4J = 2,9 Hz), 3.73 (s, 3H, OMe_5), 3.21 (t, 2H, H_{12}, ^3J = 6,9 Hz), 2.64 (t, 2H, H_{13}, ^3J = 6,9 Hz), 2,30 (s, 3H, H_{17}) **^{13}C NMR** : (100 MHz, $CDCl_3$, 25 °C) δ : 169.9 (C_{11}), 153.9 (C_2), 143.72 (C_{10}) , 142.18 (C_5) , 137.1 (C_{17}) , 133.7 (C_7), 131.2 (C_{15}-$C_{15'}$), 130.7 (C_{14}), 130.0 (C_8-$C_{8'}$), 128.6 (C_{16}-$C_{16'}$), 126.5 (C_1), 119.2 (C_9-$C_{9'}$), 111.6 (C_3), 109.8 (C_4), 107.5 (C_6), 55.8 (O<u>C</u>H_3), 55.6 (OCD_3), 37.3 (C_{12}), 29.9 (C_{13}), 21.0 (C_{18}) **HRMS** : (ES+) m/z calculated for $C_{24}H_{24}D_3N_2O_5S_2$ $[M-H]^+$, 490.1550; found 490.1545 **FT-IR :** ν(NH_{amide}) : 3325 ; ν($NH_{sulfonamide}$): 3270; ν(CH_{Me}) : 2924 ; ν(CD_{CD3}) : 2071 ; ν(C=O) : 1695 ; ν_{as}(SO) : 1330; ν_s(SO): 1153.
